# High-throughput *ex vivo* drug testing identifies potential drugs and drug combinations for NRAS-positive malignant melanoma

**DOI:** 10.1016/j.tranon.2021.101290

**Published:** 2021-11-24

**Authors:** Laura Kohtamäki, Mariliina Arjama, Siru Mäkelä, Philipp Ianevski, Katja Välimäki, Susanna Juteau, Suvi Ilmonen, Daniela Ungureanu, Olli Kallioniemi, Astrid Murumägi, Micaela Hernberg

**Affiliations:** a Helsinki University Hospital, Comprehensive Cancer Center, Department of Oncology, Helsinki and University of Helsinki, Finland; b Institute for Molecular Medicine Finland (FIMM), Helsinki Institute of Life Science (HiLIFE), Helsinki, Finland and University of Helsinki, Finland; c Department of Pathology, University of Helsinki and Helsinki University Hospital, Helsinki, Finland; d Helsinki University Hospital, Department of Surgery, Helsinki and University of Helsinki, Finland; e Applied Tumor Genomics Research Program, Research Programs Unit, University of Helsinki, Helsinki, Finland; f Science for Life Laboratory (SciLifeLab), Department of Oncology and Pathology, Karolinska Institutet, Sweden

**Keywords:** Malignant melanoma, Drug testing, Kinase inhibitors, Targeted therapy, NRAS, Personalized therapy

## Abstract

Therapy options for patients with metastatic melanoma (MM) have considerably improved over the past decade. However, many patients still need effective therapy after unsuccessful immunotherapy, especially patients with BRAF-negative tumors who lack the option of targeted treatment second line. Therefore, the elucidation of efficient and personalized therapy options for these patients is required. In this study, three patient-derived cancer cells (PDCs) were established from NRAS Q61-positive MM patients. The response of PDCs and five established melanoma cell lines (two NRAS-positive, one wild type, and two BRAF V600-positive) was evaluated toward a panel of 527 oncology drugs using high-throughput drug sensitivity and resistance testing. The PDCs and cell lines displayed strong responses to MAPK inhibitors, as expected. Additionally, the PDCs and cell lines were responsive to PI3K/mTOR, mTOR, and PLK1 inhibitors among other effective drugs currently undergoing clinical trials. Combinations with a MEK inhibitor were tested with other targeted agents to identify effective synergies. MEK inhibitor showed synergy with multikinase inhibitor ponatinib, ABL inhibitor nilotinib, PI3K/mTOR inhibitor pictilisib, and pan-RAF inhibitor LY3009120. The application of the patients’ cancer cells for functional drug testing *ex vivo* is one step further in the process of identifying potential agents and agent combinations to personalize treatment for patients with MM. Our preliminary study results suggest that this approach has the potential for larger-scale drug testing and personalized treatment applications in our expansion trial. Our results show that drug sensitivity and resistance testing may be implementable in the treatment planning of patients with MM.

## Highlights

• We established patient-derived cancer cell (PDC) lines from NRAS-positive MM tumors.
• We carried out high-throughput drug testing with PDCs against 527 oncology drugs.
• PDCs were sensitive to PI3K, mTOR, PLK1, MEK, ERK, and RAF inhibitors.
• Drug combination synergies were identified.
• Our results support application of PDCs for functional drug testing.

## Introduction

The treatment of metastatic melanoma (MM) has profoundly changed over the last decade by the introduction of checkpoint inhibitors and targeted therapies. Checkpoint inhibitors deliver durable responses in approximately 10–40% of patients with MM [1,2]. In *BRAF*-mutated melanomas, targeted combination treatment with BRAF (BRAFi) and MEK inhibitors (MEKi) is effective, leading to responses in 60–70% of patients. However, the majority of patients treated with targeted agents eventually progress due to acquired resistance [3], [4], [5].

Approximately half of all melanomas are *BRAF* mutants, and 20% are *NRAS* mutant [6, 7]. *NRAS*-mutant MM typically has an aggressive clinical course and a poor prognosis [8, 9]. Evidence suggests that these melanomas may respond to MEKi [10]. *KIT* mutations are present in 15–25% of mucosal or acral melanomas, which may be sensitive to tyrosine kinase inhibition [11], [12], [13]. The vast amount of genetic alternations found in melanoma account for the challenges and complexity of treatment planning [7, 14]. The absence of reliable predictive markers underlines the need for new personalized treatment strategies.

Traditionally, drug testing and other functional experiments have been performed using xenograft or genetically engineered mouse models. At present, mural models are irreplaceable as they produce significant information on gene function and drug efficacy *in vivo*. However, they are time-consuming, their application for routine clinical work is challenging and are unsuitable for simultaneous testing of a wide selection of drugs. Therefore, the development of other drug screening platforms is needed. The application of patients’ tumor cells to a functional precision-medicine setting has recently gained popularity due to improvements in cell culturing protocols and fast turnaround of the drug testing platform. *Ex vivo* drug testing platforms have successfully been applied in selecting effective clinical therapeutic options for patients with hematological malignancies [15,16], with promising results shown in solid cancers [17], [18], [19]. This platform enables the testing of patient-derived cancer cells (PDCs) with over 500 experimental and approved cancer-associated drugs.

In this study, we established MM PDCs, which together with melanoma cell lines were applied to a functional drug testing platform covering 527 approved and investigational oncologic drugs to identify patient-specific treatment options for patients with BRAF wild-type MM. Promising drugs were also tested in combinations.

## Materials and methods

### Patient samples and establishment of patient-derived primary melanoma cultures

This study was approved by the local ethics committee and conducted in accordance with the Declaration of Helsinki. All patients provided written informed consent prior to any procedures related to the study. Patients were eligible if they had a metastatic BRAF wild-type melanoma, superficial accessible metastasis, progressive disease, and failed standard therapy. Altogether, six patients with unresectable MM treated at the Comprehensive Cancer Center of Helsinki University Central Hospital were included. The patient’ clinical characteristics are summarized in Table 1. The mutational status of patients’ melanoma tissue samples was determined by next-generation sequencing as part of the routine analysis covering ten cancer genes, including BRAF, NRAS, and KIT, at the HUS Diagnostic Center (HUSLAB) at Helsinki University Hospital. Fresh melanoma samples were obtained for this study from easily accessible subcutaneous or lymph node metastases under local anesthesia. Tissue sample examination was performed by a dermatopathologist to confirm that the biopsy contained melanoma cells before proceeding with the establishment of the primary cancer model. The tissue samples were transported to the laboratory on ice in Hank's balanced salt solution (HBSS) and processed with a tumor dissociation kit (Miltenyi Biotec) to obtain a single cell suspension according to the manufacturer's protocol. Different media previously applied by others to culture MM primary patient-derived cancer cells were tested to establish PDCs, from which RPMI-1640 medium (Gibco) supplemented with 0.5 g/mL hydrocortisone (Sigma-Aldrich), 2% FBS, and primocin (InvivoGen) was chosen to maintain and propagate FM-MEL-2 PDCs [20]. Another media optimized by professor Meenhard Herlyn and colleagues at the Wistar Institute, Philadelphia, US is based on MCDB153 medium (Sigma-Aldrich, USA) containing 20% Leibovitz L-15 medium (Life Technologies), 5 μg/mL insulin, 15 μg/mL bovine pituitary extract, 5 ng/mL epidermal growth factor, 1.68 mM calcium chloride_,_ 2% FBS, and primocin (InvivoGen). Using this media, we were able to establish FM-MEL-3 and FM-MEL-6 PDCs [21]. Due to poor viability of the melanoma cells, and overgrowth by fibroblasts, PDCs from the other three MM biopsies could not be established.

**Table 1:** Clinical characteristics and main oncogenic aberrations of the patients.

ID	Gender	Age	Breslow	Ulceration	Histology	Mutation	Previous treatments	Metastatic Stage	Sample Location	From Dg to sampling (months)
FM-MEL-1	Female	69	2,5	0	SS	NRAS (NM_002524.4): c.181C>A,p.(Gln61Lys)	1st line: pembrolizumab	M1a	Lower extremity, left	32
FM-MEL-2	Male	56	NA	NA	NA	NRAS (NM_002524.4): c.181C>A,p.(Gln61Lys)	1st line: pembrolizumab	M1a	Abdomen, left	3
FM-MEL-3	Female	52	1,6	0	SS	NRAS (NM_002524.4): c.181C>A,p.(Gln61Lys)	Surgery, no systemic treatments	M1a	Lower extremity, left	1
FM-MEL-4	Female	66	4,9	1	Acral	WT	1st line: ipilimumab, 2nd line: pembrolizumab	M1a	Lower extremity, right	26
FM-MEL-5	Male	76	3,3	0	Acral	WT	1st line: pembrolizumab, 2nd line: TOL 3rd line: combination immunotherapy	M1c	Lower extremity, left	31
FM-MEL-6	Male	71	NA	NA	NA	NRAS (NM_002524.4): c. 182A>G, p.(Gln61Arg) / p.(Q61R) TP53 (NM_000546.5): c.329G>C, p. (Arg110Pro EGFR-gene amplification	1st line: pembrolizumab	M1c	Right axilla	10

Dg = Diagnosis, SS = Superficially spreading, NA= Not available, TOL = Combination chemotherapy consisting of Temotzolomide, Oncovin and Lomustine, WT = wild type.

### Immunohistochemistry

Tumor tissue samples were fixed in formalin and embedded in paraffin wax in the Pathology department following standard procedures. Hematoxylin and eosin staining were examined by a dermatopathologist to confirm the quality of the samples. Sections of the tumor tissue and PDCs were stained with a melanoma-specific antibody cocktail containing HMB-45, MART-1 (Melan A), and tyrosinase (#904H, Cell Marque). Images were captured using a high-resolution whole-slide scanner (Pannoramic 250 Flash III, 3DHISTECH).

### DNA extraction and cancer panel sequencing

In order to ascertain that the cancer cells that we were using for functional assays such as drug sensitivity testing is representative of the original tissue, we did cancer panel sequencing with the original tumor and DNA of the PDCs to compare the somatic mutation and copy number profiles. Genomic DNA was isolated from MM tissue biopsies and early passage PDCs using the DNeasy blood and tissue kit (Qiagen), and from the patient´s peripheral blood using the Gentra Puregene Blood kit (Qiagen) according to the manufacturer's protocols. DNA was quantified using a Qubit fluorometer (Thermo-Fisher). Detection of somatic alterations in coding sequences (289 genes) and genome‐wide copy number variants was performed using targeted sequencing. For the library preparation, in‐solution hybridization-based capture and sequencing were performed as previously described [20]. Briefly, the library was prepared using a ThruPLEX DNA-seq kit (Rubicon Genomics) and targets were enriched using a custom pan-cancer panel (Roche NimbleGen). The samples were sequenced in rapid mode on a HiSeq 2500 sequencing instrument (Illumina). Details of bioinformatic analysis, including basic quality control and identification of somatic mutations and copy number variants, was performed as previously described [22].

### Anchorage-independent growth assay

The anchorage-independent growth assay was performed as described previously [23]. Briefly, in a 6-well plate, PDCs or WM165 cell line (5 × 10^3^) were resuspended in sample-specific complete medium (1 mL, with 2% FBS and 0.3% Noble agar (#A5431, Merck)) and plated over a layer of solidified 2 × complete medium (3 mL, with 2% FBS and 0.5% Noble agar). The cultures were incubated at 37 °C, in 5% CO_2_ for 3–4 weeks, and fresh medium was added twice a week. Images of the cell growth were captured once a week.

### Cell lines

The human melanoma cell line Bowes and WM852 were kindly provided by Dr. Kaisa Lehti, University of Helsinki; and cell lines SK-MEL-28 and WM165 by Prof. Satu Mustjoki, University of Helsinki. The SK-MEL-2 cell line was purchased from ATCC. The Bowes cell line was cultured in MEM, WM852 in DMEM, SK-MEL-28, and WM165 in RPMI-1640 and SK-MEL-2 in EMEM medium. All the above cell line media were supplemented with 10% FBS, 100 U/mL penicillin, and streptomycin and cultured at 37 °C in 5% CO_2_. All cell lines were authenticated by short-tandem repeat analysis (GenePrint24 System, Promega) and screened for mycoplasma contamination using the PCR Mycoplasma Detection Set test kit (TaKaRa).

### Drug sensitivity and resistance testing (DSRT)

DSRT was performed with melanoma PDCs and cell lines as described previously [15] using the FIMM oncology drug library FO5, which includes 527 approved and investigational oncology compounds (Supplemental Table S1A). Additionally, available drug testing data from two healthy bone marrow samples were used as controls [24]. Bone marrow aspirates from healthy donors were obtained after an informed consent and were collected at the Helsinki University Hospital following protocols approved by a local ethics committee and in accordance with the Declaration of Helsinki. Drugs were dissolved in DMSO or water and plated in 384-well plates in five increasing concentrations over a 10,000-fold concentration range. The PDCs were resuspended in media and 1000–1500 cells (5000 cells for BM controls) were dispensed into wells of the pre-drugged plates with the Multidrop dispenser (Thermo Fisher Scientific) and incubated for 72 h at 37 °C. As a readout for drug efficacy, the luminescence-based measurement of cell viability (CellTiter-Glo, Promega) was performed using a PHERAstar FS plate reader (BMG Labtech). The assay was carried out similarly for measuring the drug responses in 3D spheroid culture conditions except that the PDCs were seeded on ultra-low attachment 384-well round bottom cell culture plates (Corning) pre-plated with drugs. Drug efficacy was quantified using a modified area-under-the-curve measurement called the drug sensitivity score (DSS), which was calculated as previously described [25]. In short, DSS was calculated for each drug by taking into account multiple dose-response parameters such as the IC50 value, slope, and the area under the curve (AUC) and by comparing the drug response curve in patient cells to the drug response curve of healthy cells. We used healthy bone marrow samples as controls. An in-house drug testing data analysis software called Breeze was used to calculate IC50 and perform curve fitting [26]. Drug synergy testing was performed using 8 × 8 drug concentration matrix and cell viability was assessed with CellTiter-Glo 2.0 assay (Promega). For synergy assessment, the Zero Interaction Potency (ZIP) model was applied, using the R-package SynergyFinder [27].

### Western blotting

FM-MEL-3 and FM-MEL-6 PDCs were plated onto a 6-well plate and the next day treated with indicated drug or drug-combination for 24 h. Cells were then lysed in ice-cold Triton-X lysis buffer (50 mM Tris-HCL pH 7.4, 10% glycerol, 50 mM NaCl, 1% Triton-X, 20 mM NaF) supplemented with protease and phosphatase inhibitor cocktails (Bimake). Cell lysates were incubated 15 min on ice, clarified by centrifugation (+4 °C, 20 min, 16,000 × g), resuspended in 2XSDS sample buffer and boiled at 95 °C for 5 min. Samples were then run onto an SDS-PAGE gel and Western blotting was done using the following primary antibodies: phospho-ERK1/2 Thr202/Tyr204 (#9101), ERK1 (#4696), and tubulin (#2146) from Cell Signaling Technology were used at 1:1000 dilution. IRDye 800CW Donkey anti-Mouse IgG or IRDye 680RD Donkey anti-Rabbit IgG (LI-COR) was used as a secondary antibody at 1:10,000 dilution. Blots were scanned with Odyssey CLx Imaging System (LI-COR) and images were analyzed with Image Studio Lite (LI-COR).

## Results

### Patient cases and characterization of MM patient-derived cancer cells (PDCs)

PDCs from six patients with unresectable MM were included in this pilot study. Four PDCs were NRAS p. Q61 mutant, and the two remaining ones were BRAF, NRAS, and KIT wild type. We successfully established PDCs from three patient cases with NRAS-positive MM, designated as FM-MEL-2, FM-MEL-3, and FM-MEL-6, respectively. One of these patients had no prior systemic therapy, whilst the other two had only one line of anti-PD1 therapy before sampling. The establishment of cell lines for the remaining three patients was unsuccessful. One of these patients (FM-MEL-1) responded to anti-PD1 therapy directly after sampling; thus, the cells did not grow in culture. The melanoma cells from FM-MEL-4 did not grow due to stromal cell contamination. Cells from patient FM-MEL-5 failed to grow *ex vivo*. Immunohistochemical analysis of the original tumor sample and the PDCs confirmed the expression of melanoma markers (HMB-45, MART-1 (Melan A), and Tyrosinase) in the PDCs (Supplemental Fig. S1A). All three PDCs exhibited triangular dendritic or elongated dendritic morphology, characteristic of cutaneous melanoma cultures (Supplemental Fig. S1B). Molecular profiling of the tumor sample and PDCs using the cancer panel sequencing further confirmed that all three PDCs retained the oncogenic driver mutation, specifically FM-MEL-2 and FM-MEL-3, which exhibited p. Q61K; and FM-MEL-6 exhibited p. Q61R *NRAS* mutation (Table 1). Somatic mutation and copy number variation analysis revealed that FM-MEL-2 PDCs had an *MSH3* mutation, and FM-MEL-3 PDCs had a *PIK3R1* mutation and homozygous deletion of *PTEN* and *CDKN2A*. FM-MEL-6 PDCs carried an aberrant TP53 and an amplification of *TOP1* in chromosome 20, which is associated with aggressive clinical behavior and poor prognosis [28]. Additionally, these cells exhibited amplification of the transcription factor *NFATC2*, which is a recently-discovered gene controlling the EMT-like/invasive melanoma program by regulating downstream targets such as *c-Myc, FOM1,* and *EZH2* [29] (Supplemental Fig. S2). To assess the anchorage-independent growth of FM-MEL-2, FM-MEL-3, and FM-MEL-6 PDCs in semisolid media, the primary cells were subjected to the colony forming assay in which all three samples and control melanoma cell line WM165 formed distinct colonies over four-weeks (Supplemental Fig. S3). The assay confirmed the malignant transformation potential of the primary cells.

### Drug efficacies of 527 agents to melanoma PDCs and cell lines

**Fig. 1:**
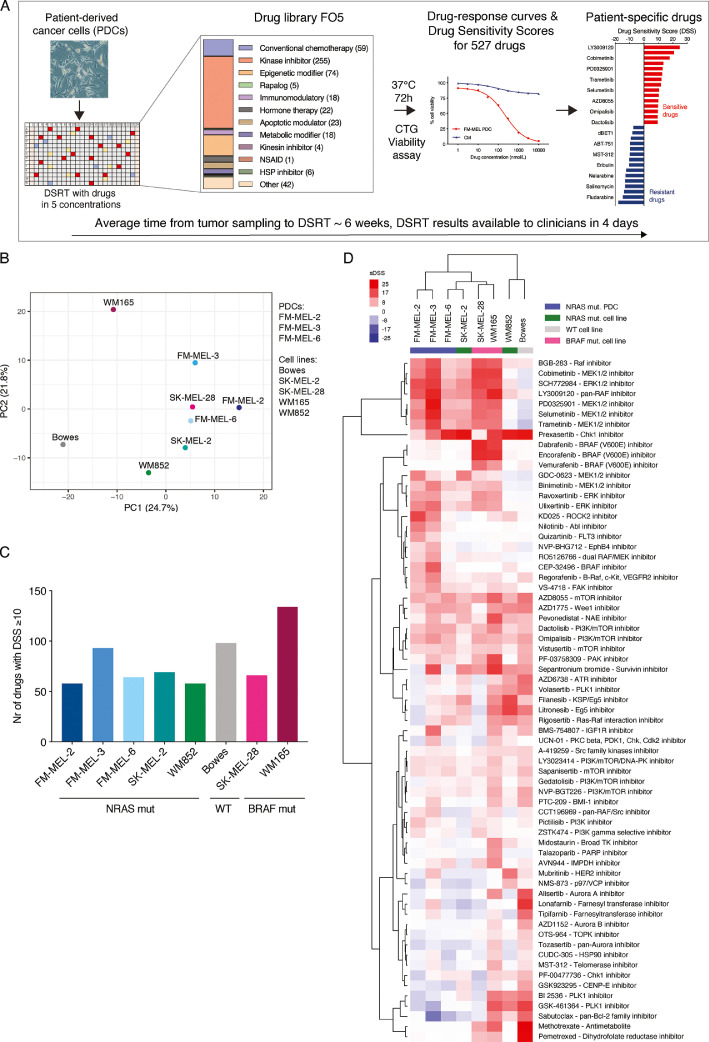
Drug response profiles of NRAS-positive FM-MEL patient-derived cancer cells (PDCs) and melanoma cell lines to 527 compounds. **A)** The schematic overview of the drug testing platform where MM PDCs and cell lines are added to pre-drugged 384-well plates and incubated for 72 h followed by cell viability analysis. Results were quantified as a selective drug sensitivity score (sDSS) to identify selective cancer cell killing compared to healthy, normal bone marrow and fibroblast cells. The median time from the sampling to the drug sensitivity and resistance testing (DSRT) experiment was approximately 6 weeks, depending on the sample. The results from the DSRT were available for the clinicians within 4 d. **B)** Principal component analysis (PCA) of drug responses from MM PDCs and cell lines to 527 drugs. **C)** Number of drugs with DSS ε 10 in each sample. **D)** Drug response heatmap for FM-MEL PDCs and melanoma cell lines in comparison to the healthy bone marrow samples. Unsupervised clustering of overlapping drugs from FIMM drug panel with a DSS of 10 or higher, in at least one sample. Columns represent samples and rows represent drugs. Red indicates a positive sDSS, while blue indicates negative sDSS in relation to the average of two healthy bone marrow samples.

To identify melanoma-specific drug responses, the three PDCs and five established cell lines were subjected to high-throughput DSRT with a panel of 527 investigational and clinically approved oncologic compounds (Fig. 1A). Among other anticancer drugs, the library includes all conventional chemotherapeutic drugs (*N* = 59), kinase inhibitors (*N* = 255), and apoptotic modulators (*N* = 23) (Supplemental Table S1A and S1B). Based on the overall drug responses to 527 drugs, three MM PDCs and three cell lines, including two NRAS-positive cell lines, grouped together in the principal component analysis, whereas the Bowes (wild type) and WM165 (BRAF V600-positive) cell lines displayed outlier profiles (Fig. 1B). Spearman's correlation analysis indicated a strong correlation among melanoma PDCs (FM-MEL-2 vs. FM-MEL-3, *R* = 0.806; FM-MEL-2 vs. FM-MEL-6, *R* = 0.811; and FM-MEL-3 vs. FM-MEL-6, *R* = 0.779) (Supplemental Fig. S4). We defined the drug sensitivity score (DSS) ≥ 10 as a threshold to classify a drug response as moderate to strong.  In general, a DSS value of 10 is considered to define a moderate-to-strong drug response sensitivity, and thus it was chosen in our studies. DSS values lower than 10 would define a low drug response sensitivity and is usually not taken into consideration. According to this, up to 134 drugs exhibited an effect in at least one sample (Fig. 1C). The BRAF V600E positive cell line WM165 exhibited the highest response to the drugs (134 drugs with DSS ≥ 10) followed by the wild-type cell line, Bowes (98 drugs with DSS ≥ 10). FM-MEL-3 had the most sensitive profile among the PDCs as the number of effective drugs with DSS ≥ 10 was 93. Across all tested samples, the median number of sensitive drugs was 69 (range, 58–134). To identify drugs that were selectively sensitive, we next compared DSSs of MM PDCs and cell lines to the DSSs of white blood cells derived from healthy donor bone marrow samples and excluded drugs that showed high efficacy also in healthy bone marrow (Fig. 1D, Supplemental Table S1C). Unsupervised clustering of the samples and drugs revealed several drugs that showed selective efficacy in MM PDCs and cell lines.

### NRAS mutated melanoma PDCs displayed similar drug response profiles

**Fig. 2:**
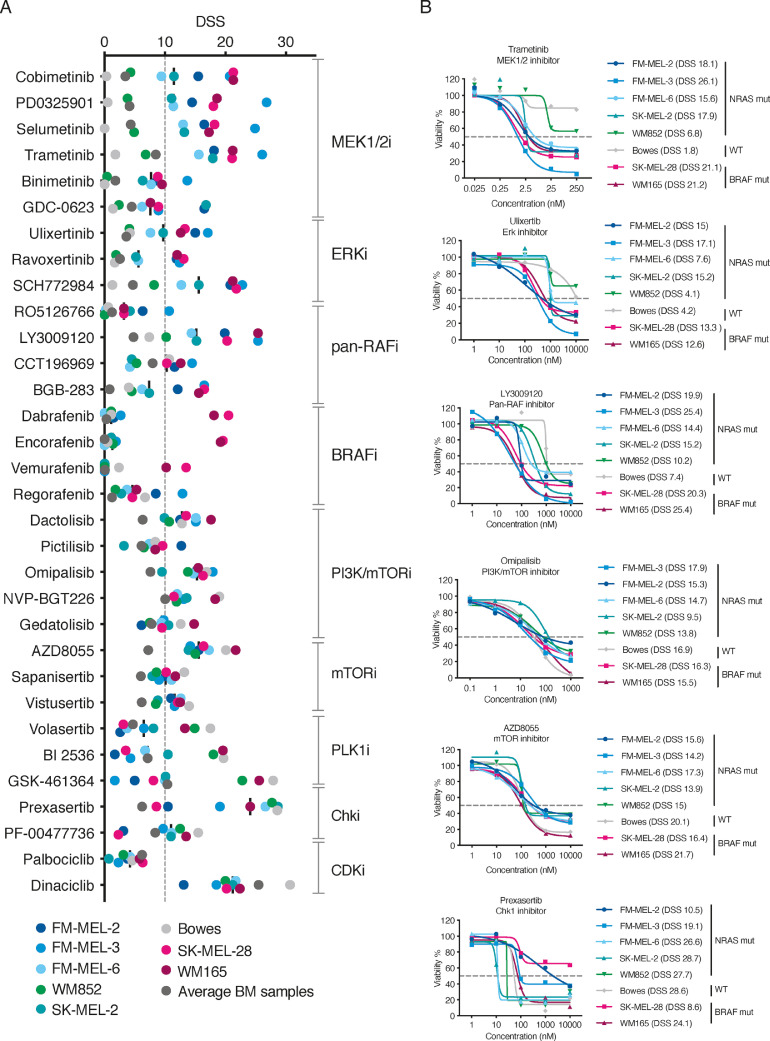
Drug efficacies of NRAS-positive FM-MEL PDCs and melanoma cell lines across different drug classes. **A)** DSSs for individual drugs across samples to MEK, ERK, pan-RAF, BRAF, PI3K/mTOR, mTOR, PLK1, Chki, and CDK inhibitors. The average DSS for melanoma-associated fibroblasts and healthy bone marrow controls is also shown. **B)** Dose response curves for selected drugs across all samples. The DSS value for each sample is indicated in parenthesis.

We identified response profiles, its specificity, and possible toxicity to healthy bone marrow samples of individual drugs belonging to the same drug class in parallel (Fig. 1D). The drug library contains 17 drugs that target different components of MEK-Ras-Raf (MAPK) signaling, a key pathway that plays a role in melanoma progression. A total of six MEKis are represented in the panel, including three clinically approved drugs used as part of BRAF V600-positive MM treatment (trametinib, cobimetinib, and binimetinib). Four MEKis (cobimetinib, PD0325901, trametinib, and selumetinib) had similar response profiles across samples, whereas binimetinib and GDC-0623 displayed lower efficacies (Fig. 2A). From these MEKis, cobimetinib was chosen for combination testing due to its efficacy shown in our PDC DSRT. All PDCs showed a high efficacy for MEKis, whereas NRAS Q61-positive melanoma cell lines, SK-MEL-2 and WM852, exhibited low responses between DSSs. ERK inhibitor SCH772984 showed the most potent efficacy (median DSS of 15) in the majority of samples compared to that of the other ERK inhibitors in the library, all of which are in the investigational phase (Fig. 2A-B). The pan-RAF inhibitor LY3009120 exhibited the highest sensitivity in NRAS-mutated PDCs and BRAF V600E mutant cell lines. Dabrafenib and encorafenib showed specific responses in BRAF-positive melanoma cell lines. Regorafenib, a multi-kinase inhibitor, had a moderate effect on melanoma cell lines.

### Drug responses to other drug classes and individual drugs

In addition to the constitutive activation of the RAS-RAF-MAPK pathway, *NRAS* mutations trigger other key pathways such as PI3K-AKT [8]. The drug library includes PI3K/mTOR inhibitors, of which dactolisib, omipalisib, and NVP-BGT226 resulted in similar responses across samples with a median DSS of 13 (Fig. 2A-B). However, the results were not FM-PDC-specific as the control samples had similar responses. AZD8055, an mTOR inhibitor, showed a selective response in all samples, apart from the bone marrow control samples, with a median DSS of 15. Polo kinase inhibitors showed varying responses. Prexasertib, a checkpoint kinase inhibitor in the developmental phase, exhibited a high response in several samples (including FM-MEL-6 and FM-MEL-3) with the highest DSS of approximately 30. The CDK inhibitor palbociclib elicited in a weak response in all tested samples, whereas dinaciclib had a median DSS of 20. However, control samples were equally sensitive to both drugs; thus, the results were unspecific for PDCs (Fig. 2A).

### Patient-specific drug responses

**Fig. 3:**
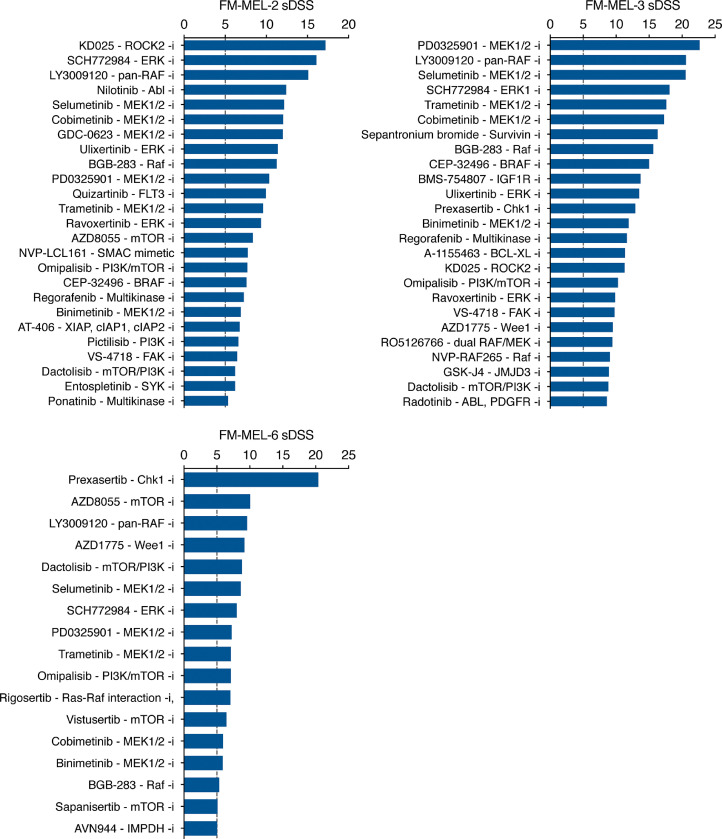
The most effective drugs for FM-MEL-2, FM-MEL-3, and FM-MEL-6 PDCs based on selective DSS in comparison to bone marrow control samples, presented in descending order.

Thirteen of the tested targeted and chemotherapeutic agents are in clinical use as treatment for MM either as single agents or in combination. Of the three MEKi, trametinib, cobimetinib, and binimetinib showed significant selective responses in all three FM-MEL PDCs, confirming the dependency of these models on RAS signaling (Fig. 3). Moreover, we identified other targeted drugs showing selective responses in PDCs, such as ABL inhibitor nilotinib, and SMAC mimetic NVP-LCL161 in FM-MEL-2 PDCs, IGF1R inhibitor BMS-754807 and CHK1 inhibitor prexasertib in FM-MEL-3 PDCs, mTOR inhibitor AZD8055, prexasertib, and Wee1 inhibitor AZD1775 in FM-MEL-6 PDCs.

In order to substantiate our findings, we chose fifteen drugs from different classes for further validation in 2D and 3D conditions. We focused on MEK inhibitors trametinib, cobimetinib, binimetinib, and selumetinib; ERK inhibitors SCH772984, and ulixertinib; pan-Raf inhibitor LY3009120; pan-tyrosine kinase and BCR-ABL inhibitor nilotinib; FLT3 inhibitor quizartinib; mTOR/PI3K inhibitors dactolisib, omipalisib, and AZD8055; Wee1 inhibitor AZD1775, Chk1 inhibitor prexasertib and ROCK2 inhibitor KD025.  The validation experiments were performed with all three PDCs in 2D and 3D conditions in triplicates for each drug concentration (Supplemental Table S2). As shown in Supplemental Fig. S5, most of the 15 drugs showed PDC-specific drug sensitivities that were similar to those observed during initial drug sensitivity testing, and the expected drug responses were observed in addition to 2D also in 3D spheroid models.  Some of the tested hits showed sensitivity only in one PDC model, like for example FLT3 inhibitor quizartinib - the validation experiments confirmed a FM-MEL-2 specific response for this drug. ROCK2 inhibitor KD025 exhibited sensitivity in FM-MEL-2 and FM-MEL-3 PDCs but validation screen confirmed the response of this drug only for FM-MEL-2 PDCs. Chk1 inhibitor prexasertib showed high sensitivity in the initial drug screen for FM-MEL-3 and FM-MEL-6 PDCs, however, the validation of 2D and 3D screens were able to confirm this finding only for FM-MEL-3 PDCs.

### Cobimetinib in combination with other targeted drugs

As monotherapy regimens lead faster to the development of acquired resistance, combination regime testing was a reasonable way to proceed. We sought to identify potentially effective drug combinations that would synergize with MEKi by combining a single concentration of MEKi (cobimetinib) to FO5 drug panel. Cobimetinib was chosen as the MEKi backbone because of superior efficacy in our PDCs and due to other published preclinical and clinical combination results from cobimetinib together with other targeted agents [30]^,^ [31].

**Fig. 4:**
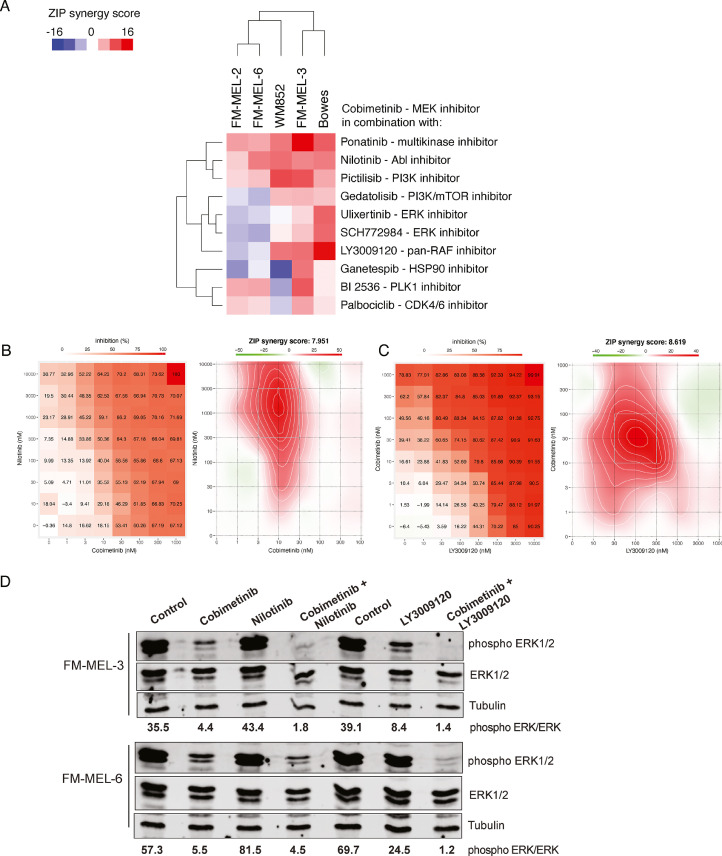
MEK inhibitor cobimetinib in combination with other targeted drugs synergistically inhibits growth of FM-MEL PDCs and cell lines. **A)** The ZIP synergy scores for each cobimetinib combination are shown as a heatmap. The data is shown from one representative experiment of at least two replicates. As an examples the individual synergy plots for FM-MEL-6 PDCs presenting cobimetinib and nilotinib combination **(B)** and FM-MEL-3 PDCs presenting cobimetinib and LY3009120 combination **(C)** are shown. Synergy plots for all the other combinations can be found in the Supplemental Fig. S6. **D)** Western blots for phospho-ERK1/2 and total ERK1/2 levels in lysates of FM-MEL-3 and FM-MEL-6 PDCs treated for 24 h with DMSO, cobimetinib (100 nM), nilotinib (1000 nM), LY3009120 (100 nM) or combination of cobimetinib either with nilotinib or LY3009120 as indicated. Tubulin was used as a loading control. The values indicate the quantification of pERK levels in ratio to the total ERK levels.

To validate these findings, we selected ten drugs to be tested in drug combination matrices where seven different concentrations of two drugs were combined in an 8 × 8 matrix. The selected ten drugs included Abl, PI3K, PI3K/mTOR, ERK, pan-RAF, HSP90, PLK1 and CDK4/6, and multi kinase inhibitors (Fig. 4A, Supplemental Table S3).

Ponatinib is a multi-tyrosine kinase inhibitor and is known to have efficacy in chronic myeloid leukemia [32]. Combining it to BRAFi was found beneficial in a preclinical model for anaplastic thyroid cancer [33]. Nilotinib is an ABL inhibitor, which seems to have potency to reverse and even prevent resistance from developing to BRAF and MEK inhibitor therapy in a xenograft model [34]. The combination of cobimetinib and ponatinib or nilotinib showed synergistic responses across all tested samples, with highest effect in FM-MEL-3 PDCs (Fig. 4A and B, Supplemental Table 2). Moreover, our Western blot results showed a strong inhibition of pERK levels in FM-MEL-3 and FM-MEL-6 PDC samples treated with a combination of cobimetinib and nilotinib compared to single treatment (Fig. 4D) confirming our drug-synergy data and offering a molecular basis of the identified combinatorial drug effect.

Pictilisib is a selective pan-inhibitor of class I PI3K and a weak inhibitor of class II, III, and IV PI3K and has shown efficacy preclinically in BRAF and KRAS mutant cell lines combined to MEKi [35]. The inhibition of MAPK signaling can lead to PI3K/AKT route activation creating a rationale for the combination of MEKi and PI3Ki [36]. Pictilisib showed synergy with cobimetinib across all our samples, whereas the combination of Pi3K/mTOR inhibitor gedatolisib and cobimetinib had less effect in FM-MEL-2 and FM-MEL-6 PDCs. Similarly, the combination of cobimetinib with pan-RAF inhibitor LY3009120 resulted in a synergistic effect and a total inhibition of pERK levels as shown by Western blot analysis in FM-MEL-3 and FM-MEL-6 PDCs (Fig. 4D), whereas single treatments had lesser effect on pERK inhibition. Combination of HSP inhibitor ganetespib and cobimetinib showed the highest synergy in FM-MEL-3 PDCs. PLK1 inhibitor BI2536 and CDK4/6 inhibitor palbociclib together with cobimetinib resulted in synergy in FM-MEL-3, FM-MEL-2, and FM-MEL-6 PDCs (Fig. 4A and B, Supplemental Table 2). Similarly, in our PDCs, PLK1i BI2536 together with cobimetinib resulted in synergy (Fig. 4A and B, Supplemental Table 2, Supplemental Fig. S6).

## Discussion

Melanoma is a heterogeneous disease with an extensive genetic variety and occasionally unpredictable course. Approximately 40% of patients treated with immunotherapy are long-term survivors [37,38]. In the majority of BRAF-positive melanoma patients, combination therapy with BRAFi and MEKi eventually leads to the development of acquired resistance [39]. For BRAF wild-type patients in whom immunotherapy is unsuccessful, no 2nd line therapy options with survival benefit exist. Consequently, there is still a substantial unmet need to develop effective treatment options particularly for BRAF wild-type patients.

Approximately 20% of melanoma patients have NRAS-mutant tumors [7]. Due to difficulties in direct targeting of RAS [40], efforts have been made to target its downstream pathways including MAPK and PI3K/AKT/mTOR. Monotherapy with MEKi has shown some efficacy in NRAS mutant melanoma in preclinical and phase I setting [41,42]. However, the NEMO trial assessing binimetinib efficacy resulted in only minor improvement of progression-free survival and showed no survival benefit compared to dacarbazine [10]. In clinical practice, drug resistance is a common problem occurring during single-agent targeted therapy. In BRAF-mutated melanoma, the combination of MEKi and BRAFi has increased treatment efficacy significantly [43,44]. Combinations of targeted agents may increase efficacy in NRAS-mutant melanoma cell lines [45,46], but despite promising preclinical testing other combinations than BRAFi and MEKi have led to disappointments due to dose-limiting toxicity in phase I trials limiting their use in the clinic [31,47].

As expected, we found that high-throughput drug testing with three NRAS-positive PDCs revealed strong responses of PDCs to MAPK pathway inhibitors, allowing to explore various MEK specific drug combinations. In addition, also a new observation was that the NRAS mutant PDCs were sensitive to the ABLi nilotinib alone as well as in combination with cobimetinib. In order to enhance treatment efficacy and to prevent development of resistance, combination regimens need to be explored [5,48]. Our results with drug combinations show significant synergy improving efficacy. Ponatinib, nilotinib, and pictilisib were each combined with cobimetinib. All of these duplet combinations resulted in substantial synergy in two of our PDCs (FM-MEL-2 and FM-MEL-6). These combinations would be interesting to evaluate further in a clinical trial setting.

Studies on PDCs from glioblastoma, ovarian, and hematological cancer patients indicate that DSRT is a promising treatment stratification platform [18,49,50]. In our study, the cultured PDCs were ready for drug testing approximately six weeks after excision, and then the DSRT results were available to the clinicians within a median of 4 days. Since melanoma patients often have cutaneous, subcutaneous, or lymph node metastases that are easily excised, DSRT may be implementable in the individual treatment planning of patients with metastatic melanoma for whom standard therapies have failed.

The number of patients was small in our study; hence, drawing conclusions must be made with caution and in accordance with other similar findings [19]. The PDCs from FM-MEL-5 with several lines of anticancer therapy did not grow *ex vivo.* Considering that previous treatments may have a negative effect on *ex vivo* cell growth, sampling in clinical practice should preferably be done as soon as possible after the diagnosis of metastatic disease, or at the latest after unsuccessful first-line immunotherapy. Additionally, early sampling would provide more time for cell culture establishment and DSRT conduction.

We acknowledge the limitations of the DSRT approach. The availability of experimental drugs can be challenging. At present, the tumor microenvironment is not cloneable; hence, DSRT is likely to portray more optimistic results than *in vivo*. Monotherapy with targeted agents typically leads to drug resistance, resulting in the need for testing combinations. Toxicity resulting from targeted therapy combinations is a challenge and can only be evaluated in an early phase clinical trial setting by testing sequential or intermittent drug administration. Since the toxicity of targeted therapy combinations has limited their use, DSRT could facilitate the selection of potential combination strategies for clinical trials. In the case where DSRT would indicate multiple effective therapy options for a patient in need of therapy, the selection of treatment would probably be made taking into account both the drug availability and the toxicity profile of the selected drug.

To substantiate and expand on our findings, we are recruiting new patients. As melanoma cells are consistently chemotherapy-resistant, we plan to modify the drug panel incorporating only targeted therapy agents. In this study, we used a broader panel of drugs to elucidate the most effective potential drugs. Fewer drugs on the testing panel will reduce costs and reduce the need for viable tumor cells, shortening the incubation period of the PDCs. Furthermore, within this study we aim to offer participating patients without standard treatment options in need of new therapy a single agent treatment based on the patient's DSRT panel test results. Testing of combinations could only be done within an early phase dose escalation trial in order to safely evaluate the toxicity profile.

For patients with BRAF wild-type MM whose first-line immunotherapy was unsuccessful, the need for further effective treatment options is imminent. According to our preliminary results, *ex vivo* drug testing with PDCs may provide a step closer in the process for identification of personalized treatment options for these patients*.* The results of our approach indicate potential for larger-scale drug testing and personalized treatment applications within our expansion trial.

## Author contribution

LK, SM, AM, and MH designed the study. SM and MH recruited patients, collected patient consent, and collected clinical information. SI did the surgery. MA, KM, AM, and DU conducted the experiments and analyzed the data. LK, MA, SM, AM, DU and MH wrote the manuscript with input and scientific advice from KV, SI, SJ, and OK. Funding acquisition was performed by OK and MH.

## Funding

This study was supported by grants from the Academy of Finland Centre of Excellence for Translational Cancer Biology (grant 307366), Academy of Finland (grant 333583), Cancer Society of Finland, FICAN South, Finnish Melanoma Group Grant, Helsinki University Cancer Center Grant (DNRO 160/13/030216), and Sigrid Jusélius Foundation. OK received support from the 10.13039/501100004359VR Research Environment grant, KAW, SFS, and Vinnova. Open access funded by Helsinki University Library.

## Availability of data and materials

Data from the drug screening are included in this published article and its supplementary files. All other data used in the current study are available from the corresponding author on reasonable request.

## Declaration of Competing Interest

OK is a co-founder and a board member of Medisapiens and Sartar Therapeutics and has received royalty on patents licensed by Vysis-Abbot. His research group has a Vinnova-funded collaborative program with Astra-Zeneca, Labcyte, Takara Biosciences and Pelago, which are not related to this work. LK has received consulting or advisory honoraria form Roche and Amgen; speakers’ bureau honoraria from BMS, and Roche; travel and accommodations expenses from Merck. MH has received consulting or advisory honoraria from Merck, Bristol-Myers Squibb, Incyte, Novartis, and Roche; speakers’ bureau honoraria from Merck, Novartis, and Bristol-Myers Squibb. SM has received consulting or advisory honoraria from Amgen, Bristol-Myers Squibb, Merck Sharp & Dohme, Merck Group, Novartis, Roche, and Sanofi; speakers’ bureau honoraria from Bristol-Myers Squibb, Merck Sharp & Dohme, and Sanofi; travel and accommodations expenses from Amgen, Novartis, and Roche. All remaining authors have declared no conflicts of interest.

## Appendix Supplementary materials

Image, application 1

Image, application 2

Image, application 3

Image, application 4

Image, application 5
