# Supplementary material for: High-throughput ex vivo drug testing identifies potential drugs and drug combinations for NRAS-positive malignant melanoma
Source: Transl Oncol. 2021 Nov 24;15(1):101290. doi: 10.1016/j.tranon.2021.101290 (PMC8633005; doi:10.1016/j.tranon.2021.101290)

Supplemental Figure S6

FM-MEL-2

Ponatinib (nM) & Cobimetinib (nM)

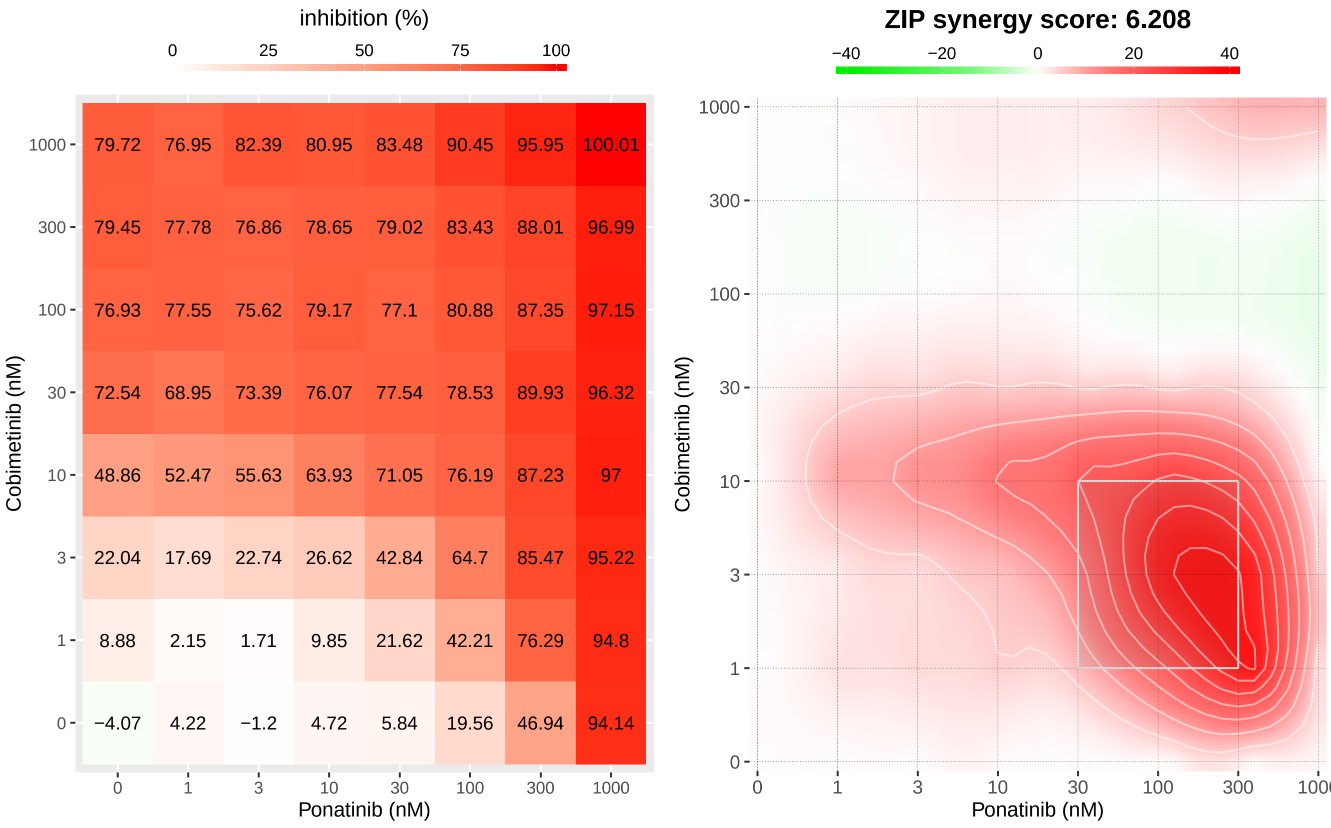

FM-MEL-2

Cobimetinib (nM) & Nilotinib (nM)

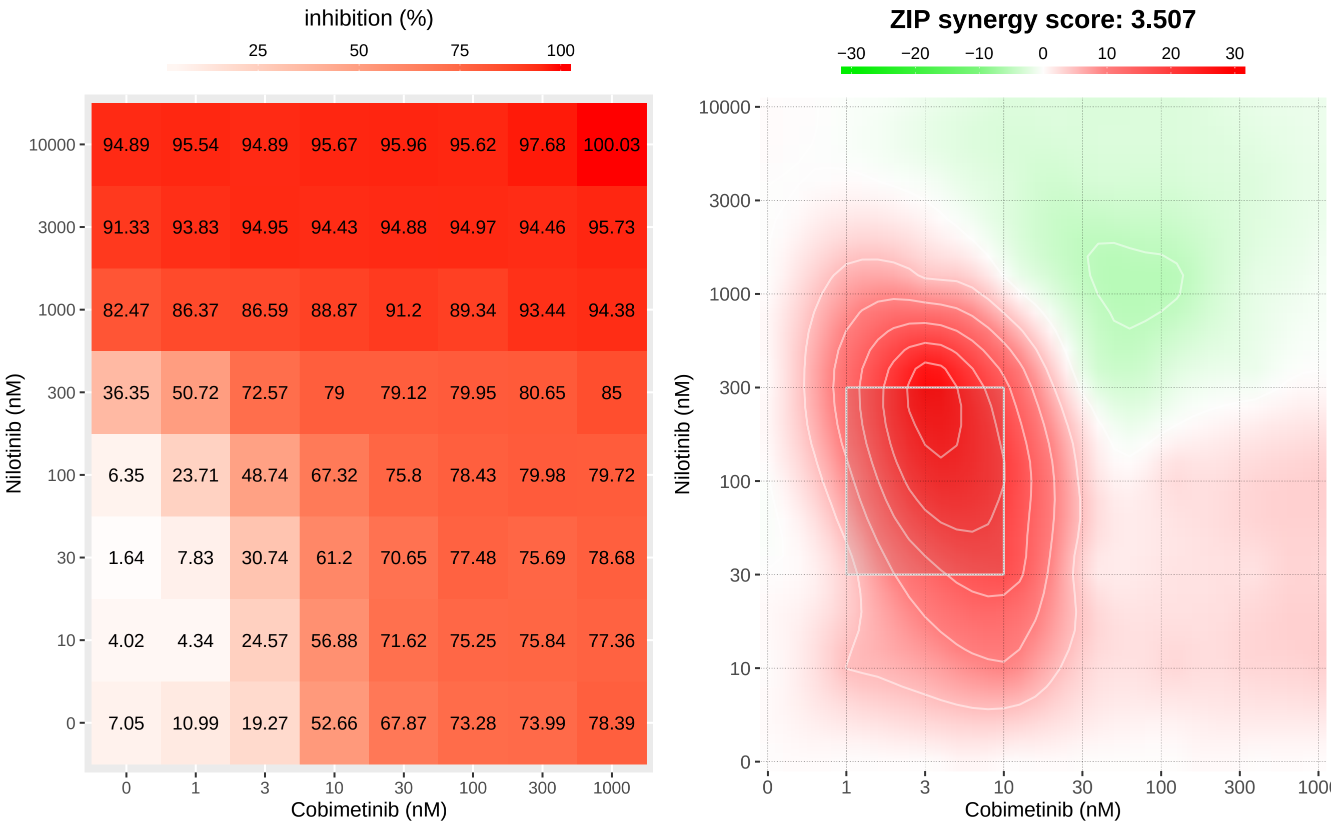

FM-MEL-2

Pictilisib (nM) & Cobimetinib (nM)

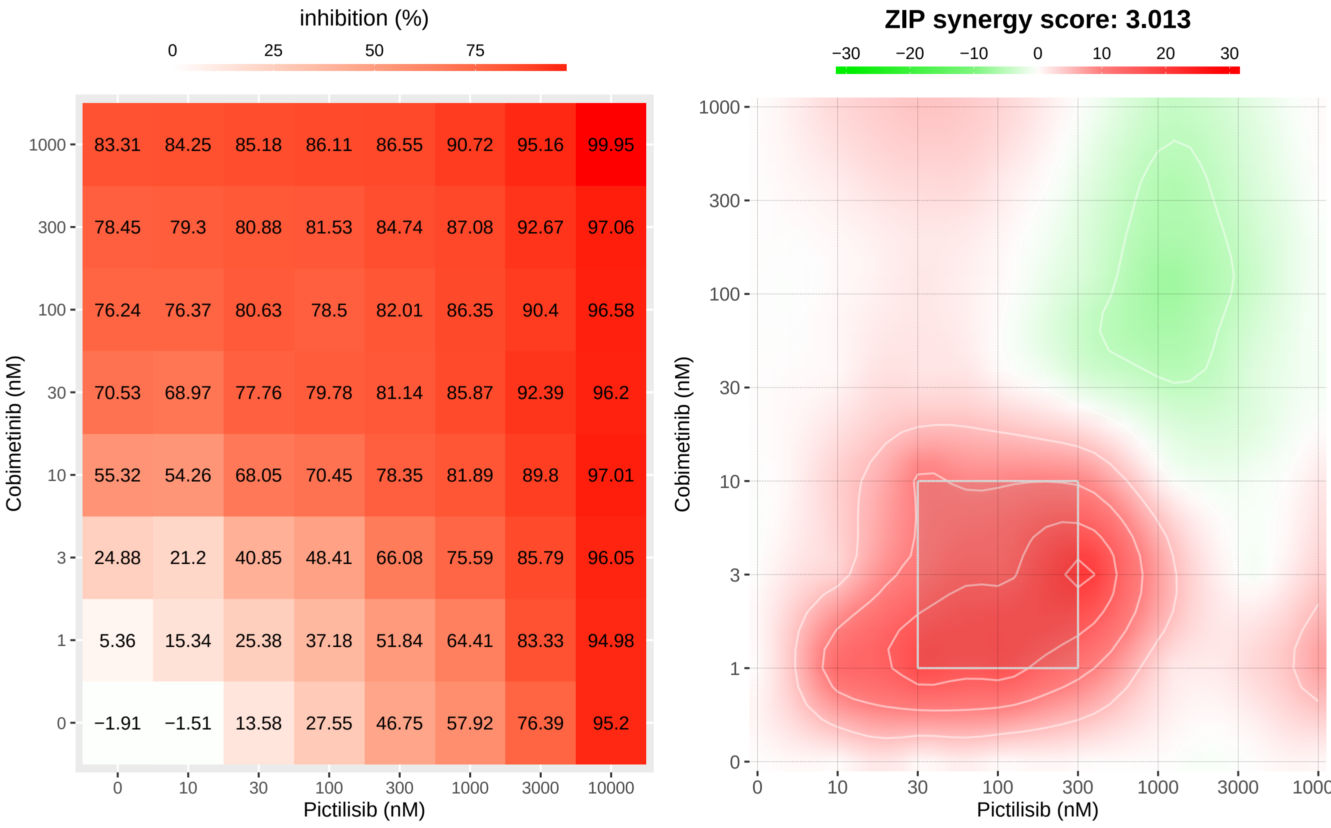

FM-MEL-2

Cobimetinib (nM) & Gedatolisib (nM)

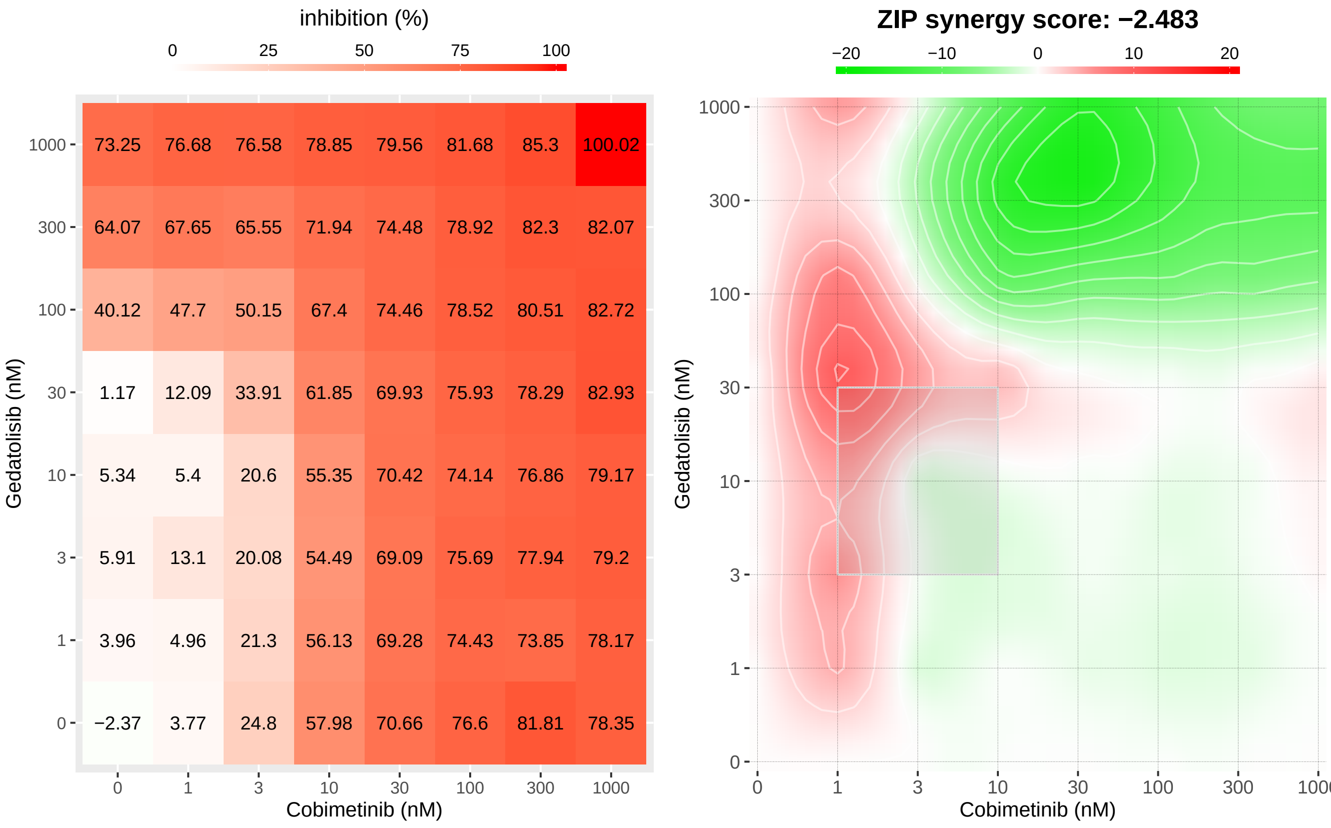

FM-MEL-2

Ganetespib (nM) & Cobimetinib (nM)

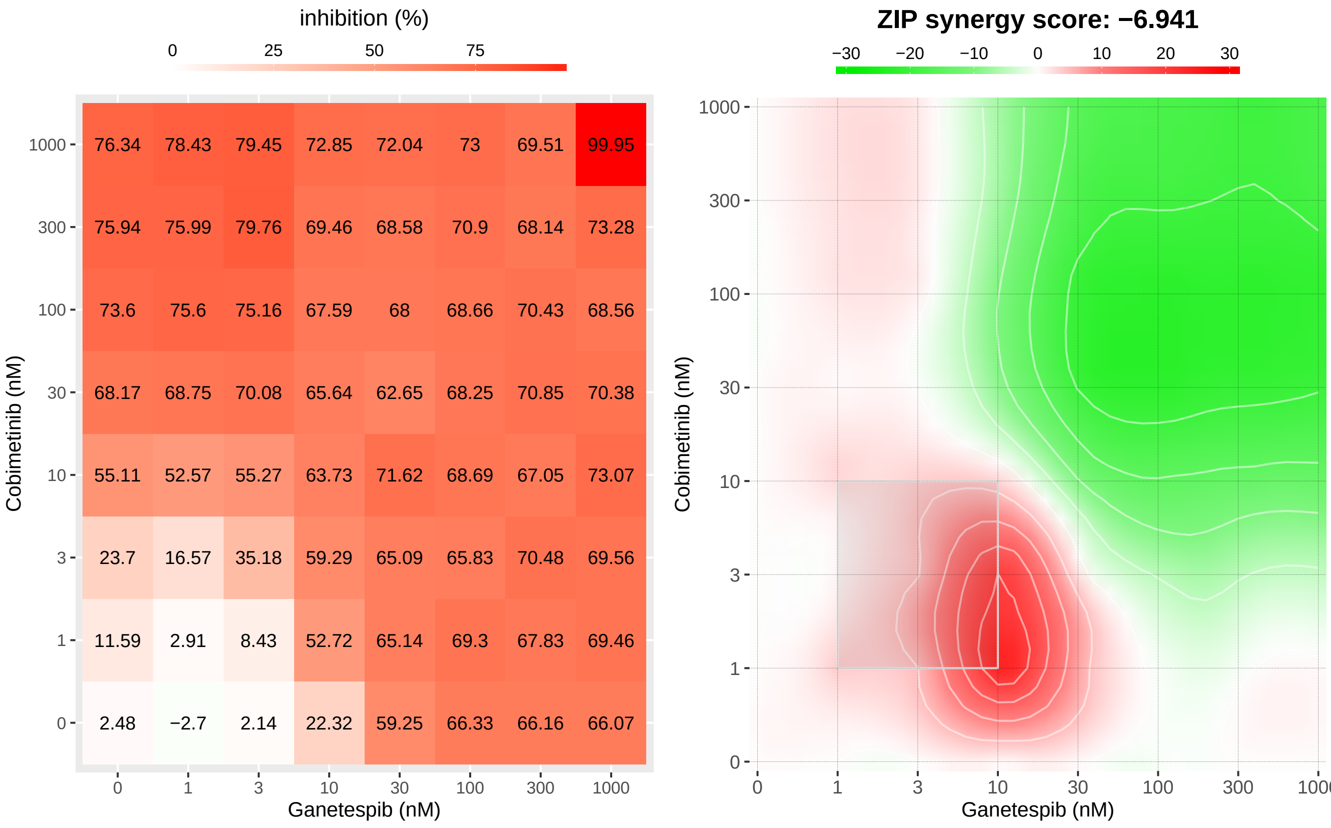

FM-MEL-2

Cobimetinib (nM) & Ulixertinib (nM)

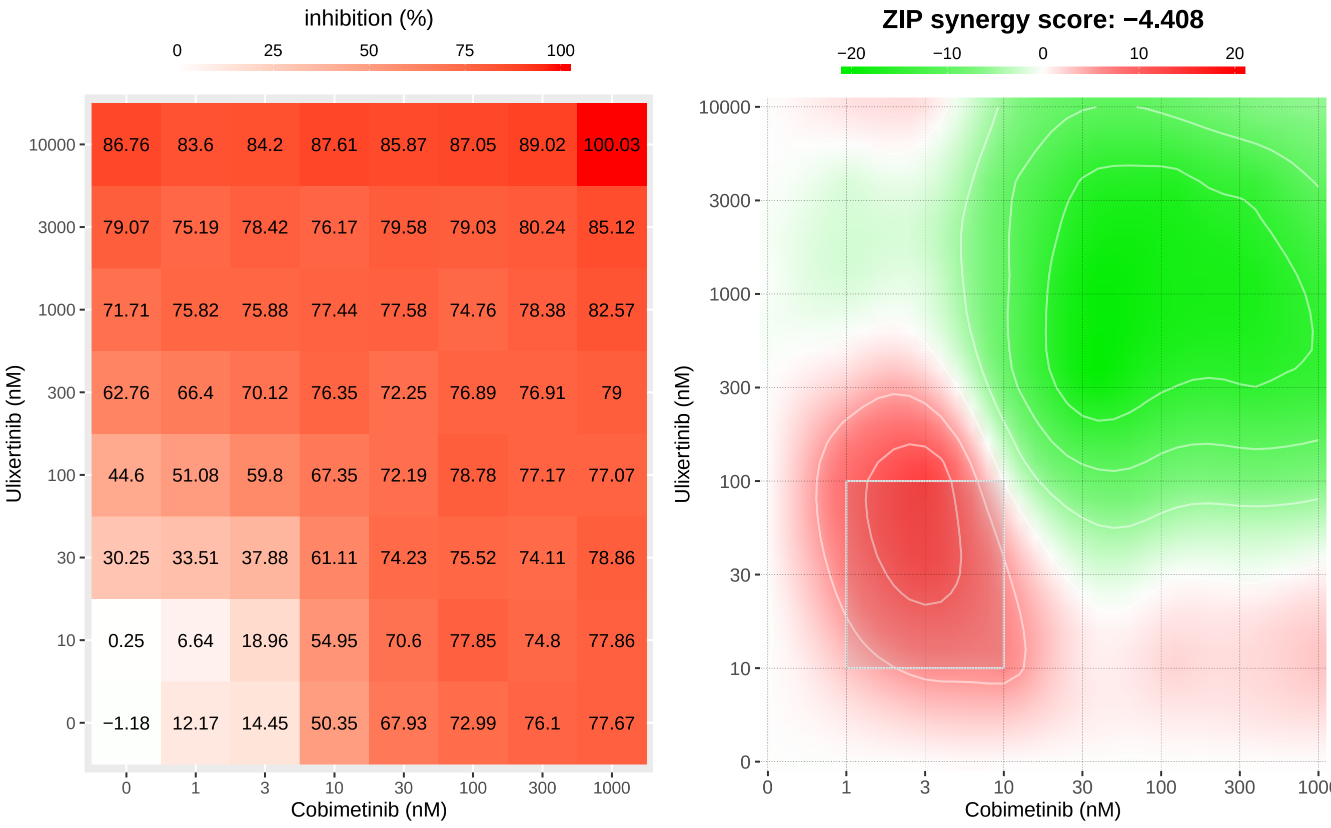

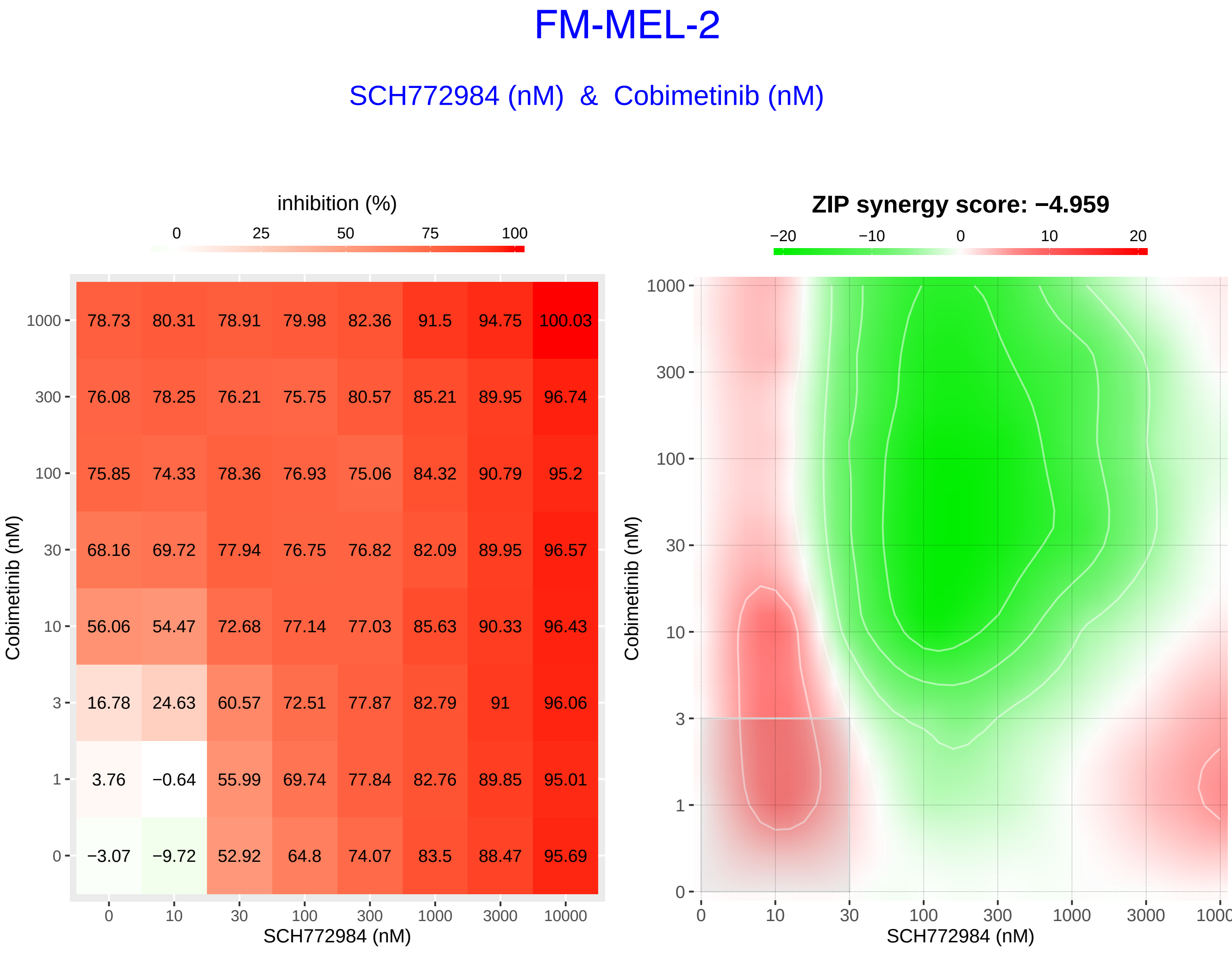

FM-MEL-2
SCH772984 (nM) & Cobimetinib (nM)
inhibition (%)
0 25 50 75 100
Cobimetinib (nM)
1000: 78.73 80.31 78.91 79.98 82.36 91.5 94.75 100.03
300: 76.08 78.25 76.21 75.75 80.57 85.21 89.95 96.74
100: 75.85 74.33 78.36 76.93 75.06 84.32 90.79 95.2
30: 68.16 69.72 77.94 76.75 76.82 82.09 89.95 96.57
10: 56.06 54.47 72.68 77.14 77.03 85.63 90.33 96.43
3: 16.78 24.63 60.57 72.51 77.87 82.79 91 96.06
1: 3.76 −0.64 55.99 69.74 77.84 82.76 89.85 95.01
0: −3.07 −9.72 52.92 64.8 74.07 83.5 88.47 95.69
0 10 30 100 300 1000 3000 10000
SCH772984 (nM)
ZIP synergy score: −4.959
−20 −10 0 10 20
1000 300 100 30 10 3 1 0
Cobimetinib (nM)
0 10 30 100 300 1000 3000 1000
SCH772984 (nM)

FM-MEL-2

Cobimetinib (nM) & LY3009120 (nM)

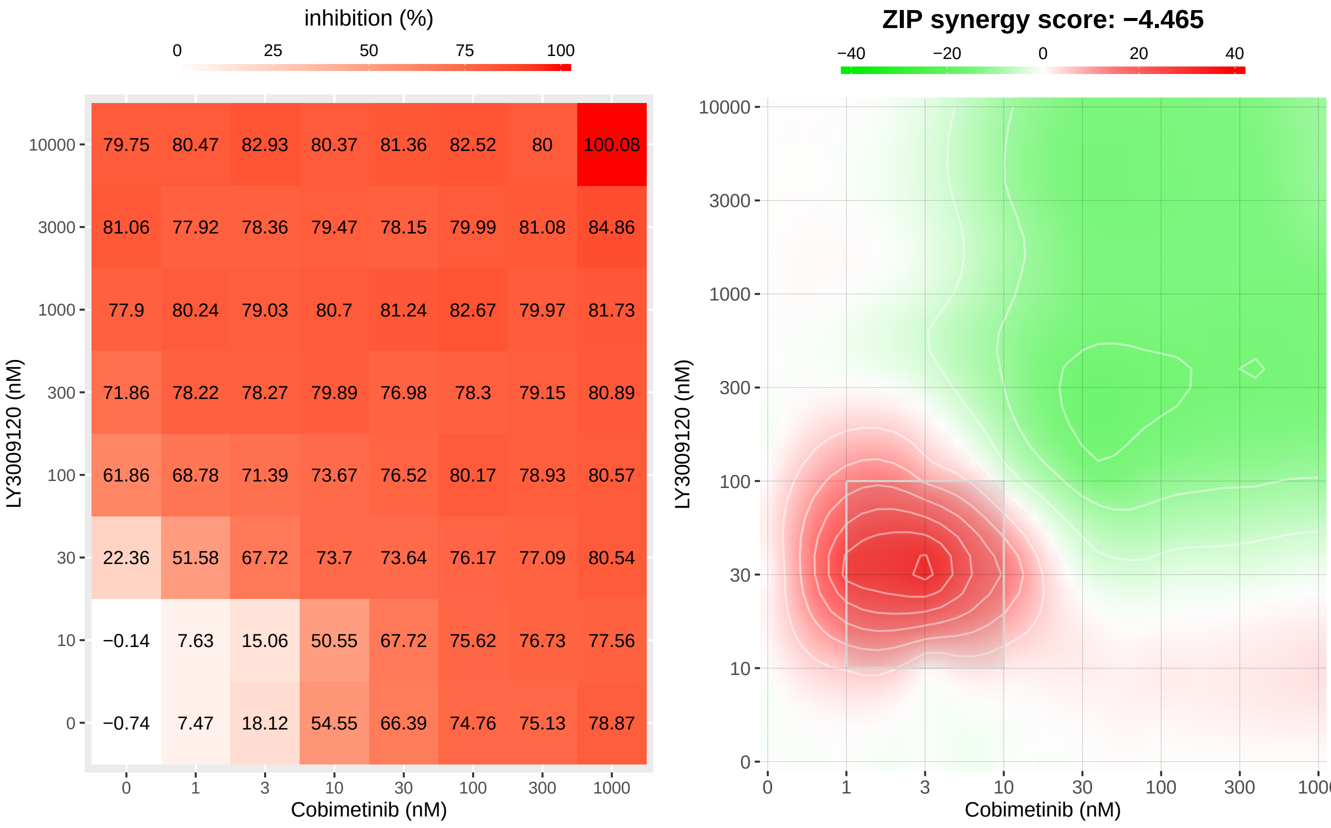

FM-MEL-2

Cobimetinib (nM) & BI 2536 (nM)

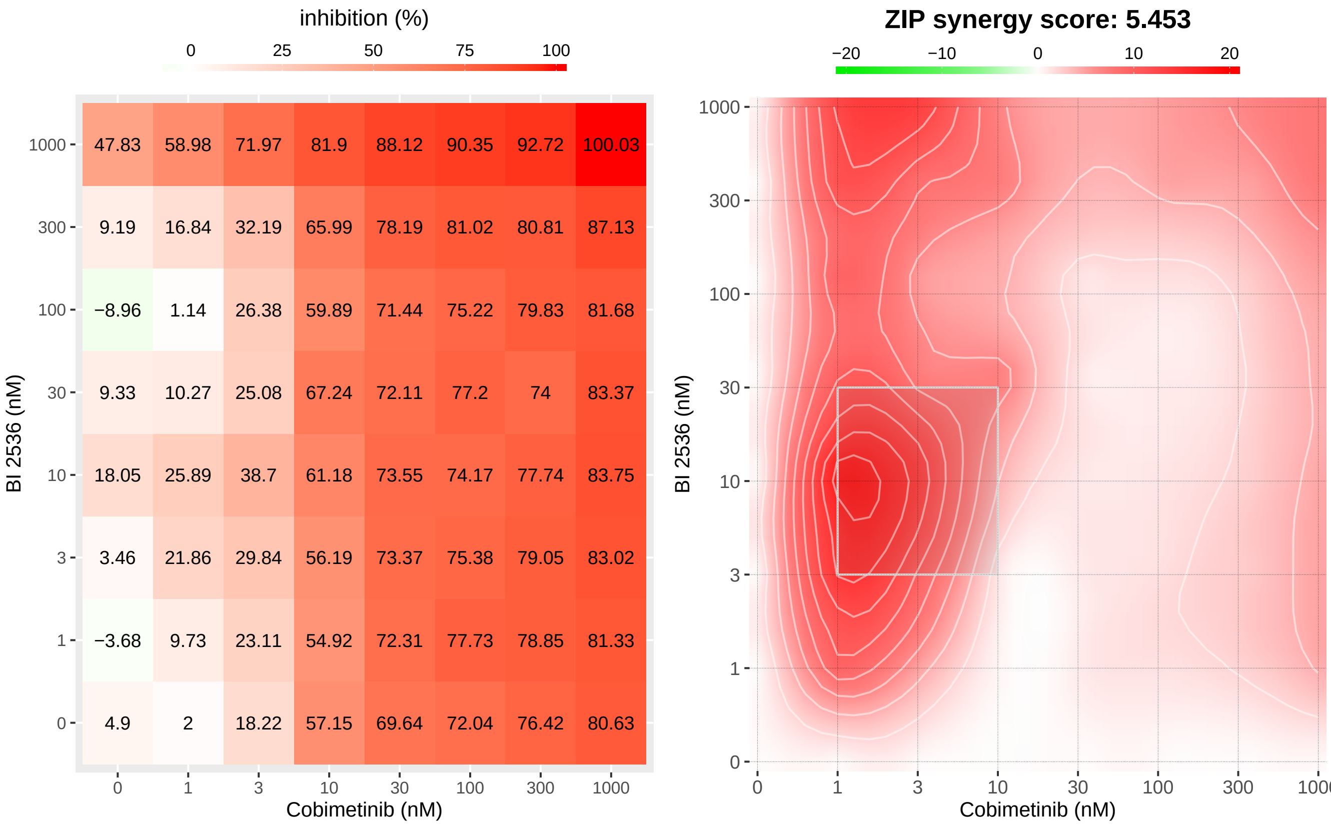

FM-MEL-2

Cobimetinib (nM) & Palbociclib (nM)

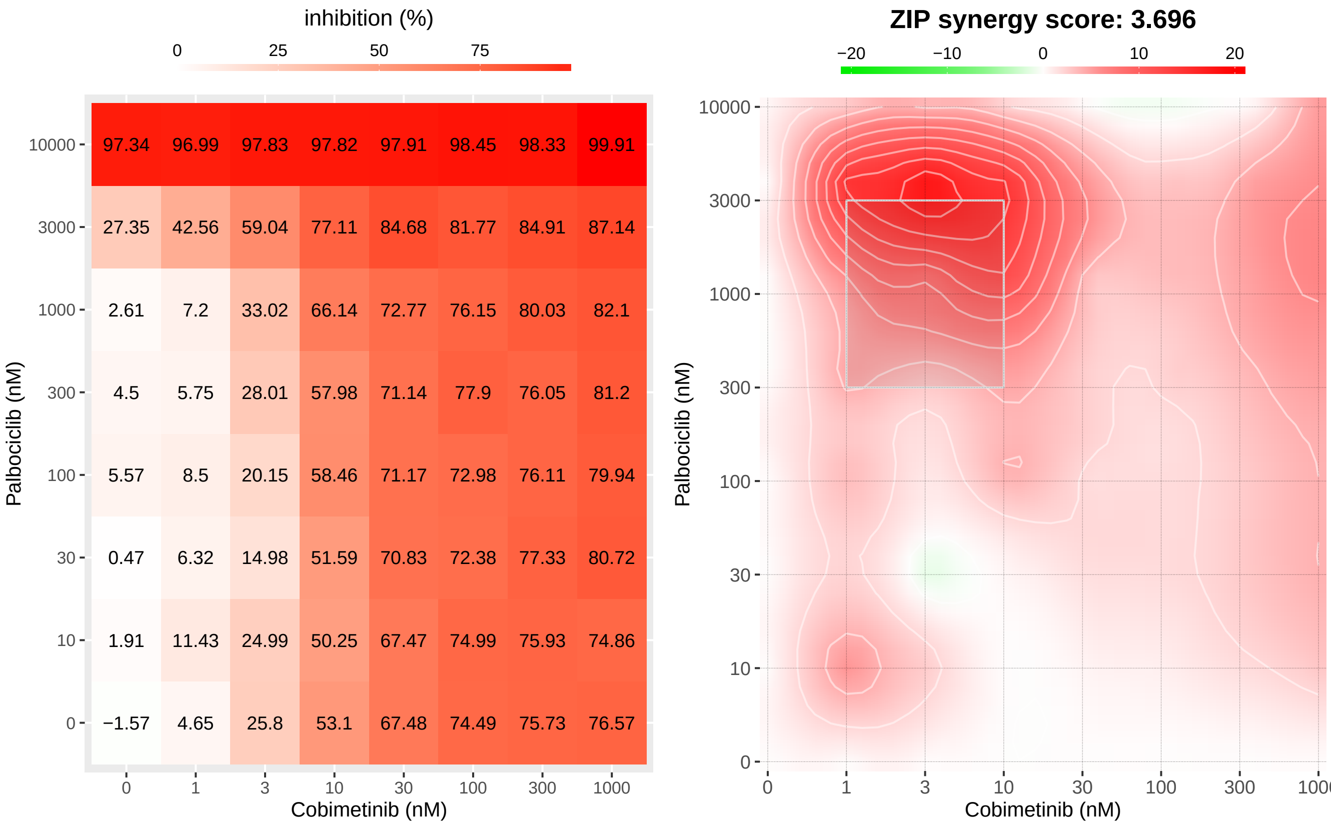

FM-MEL-3

Cobimetinib (nM) & Ponatinib (nM)

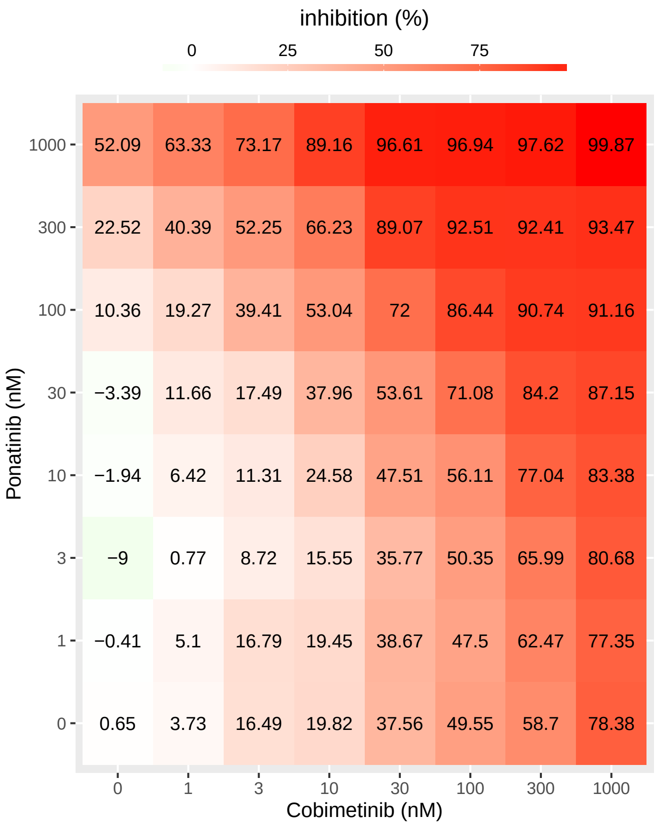

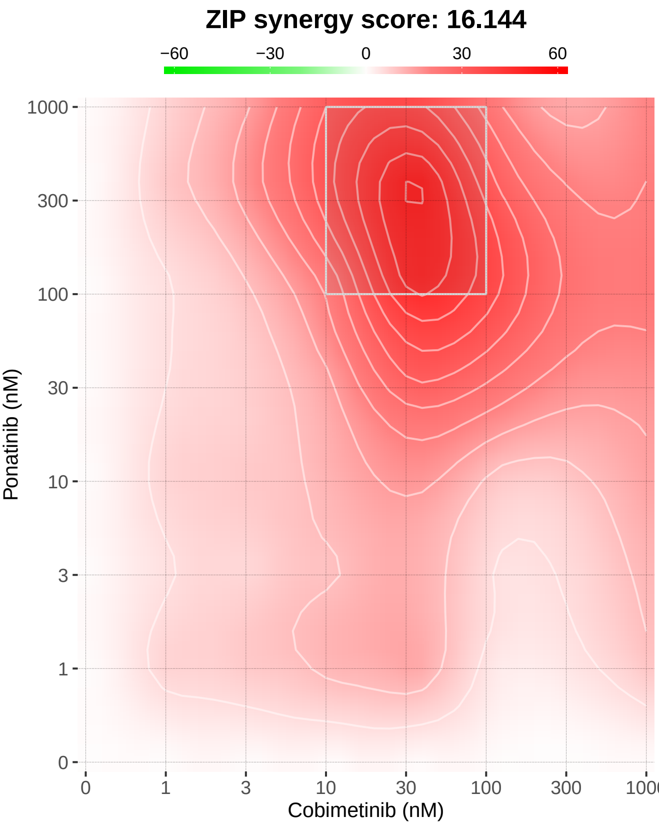

FM-MEL-3

Cobimetinib (nM) & Nilotinib (nM)

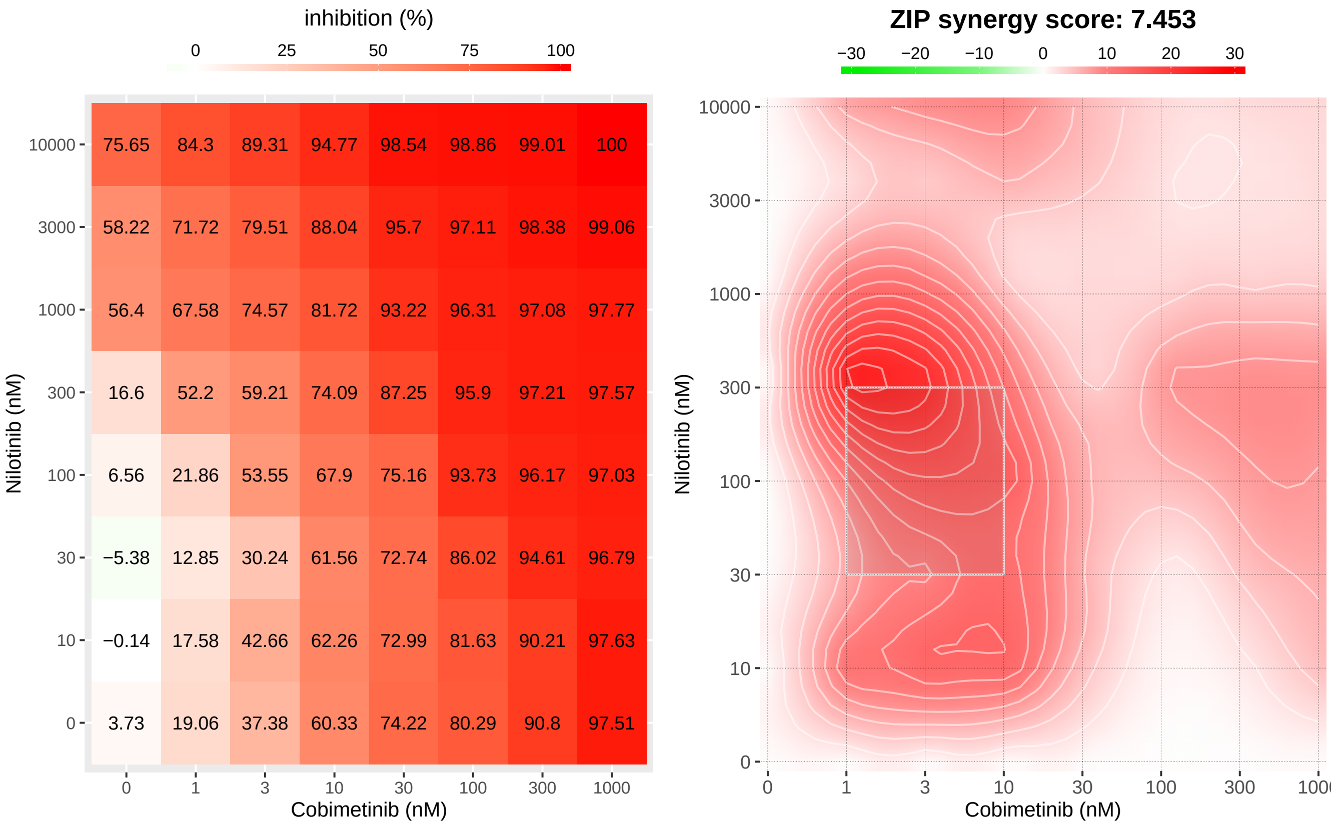

FM-MEL-3

Pictilisib (nM) & Cobimetinib (nM)

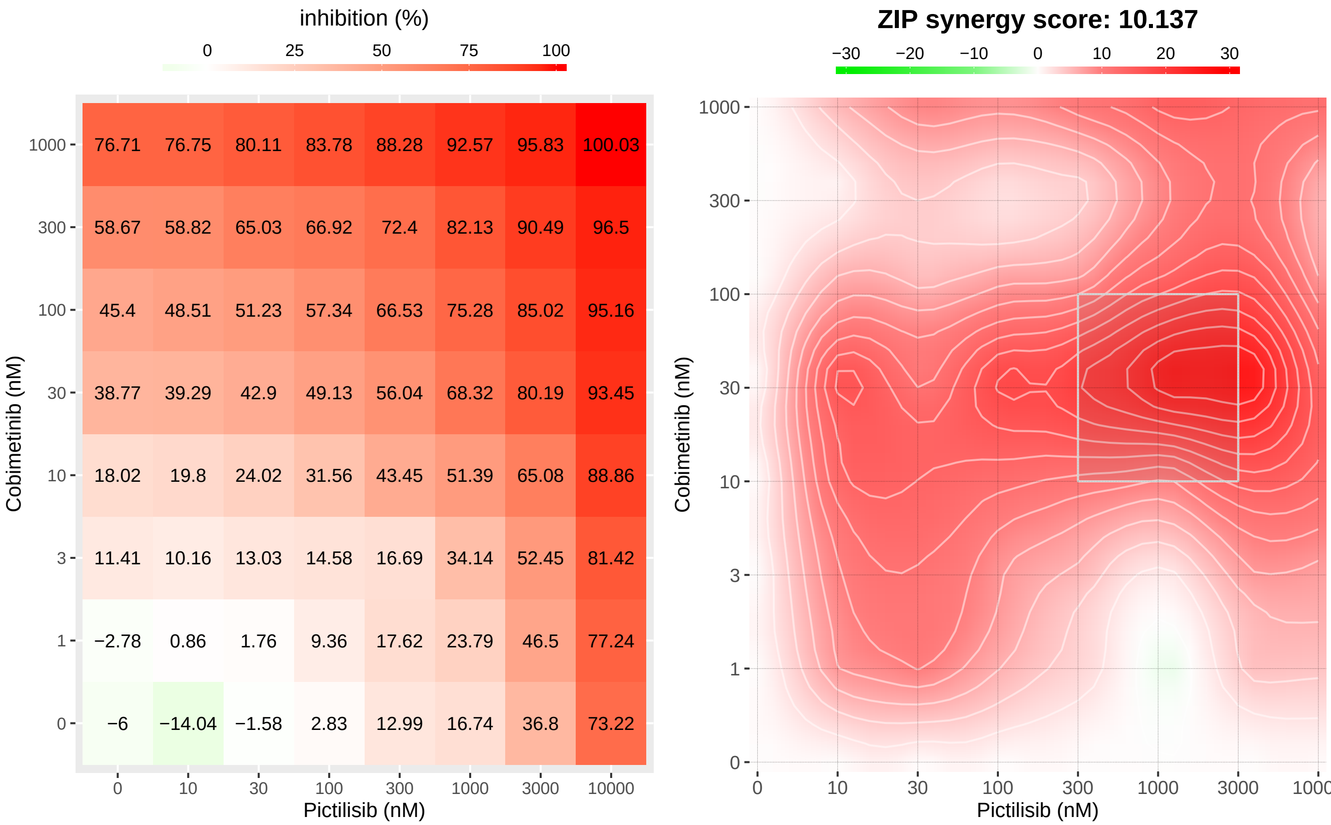

FM-MEL-3

Cobimetinib (nM) & Gedatolisib (nM)

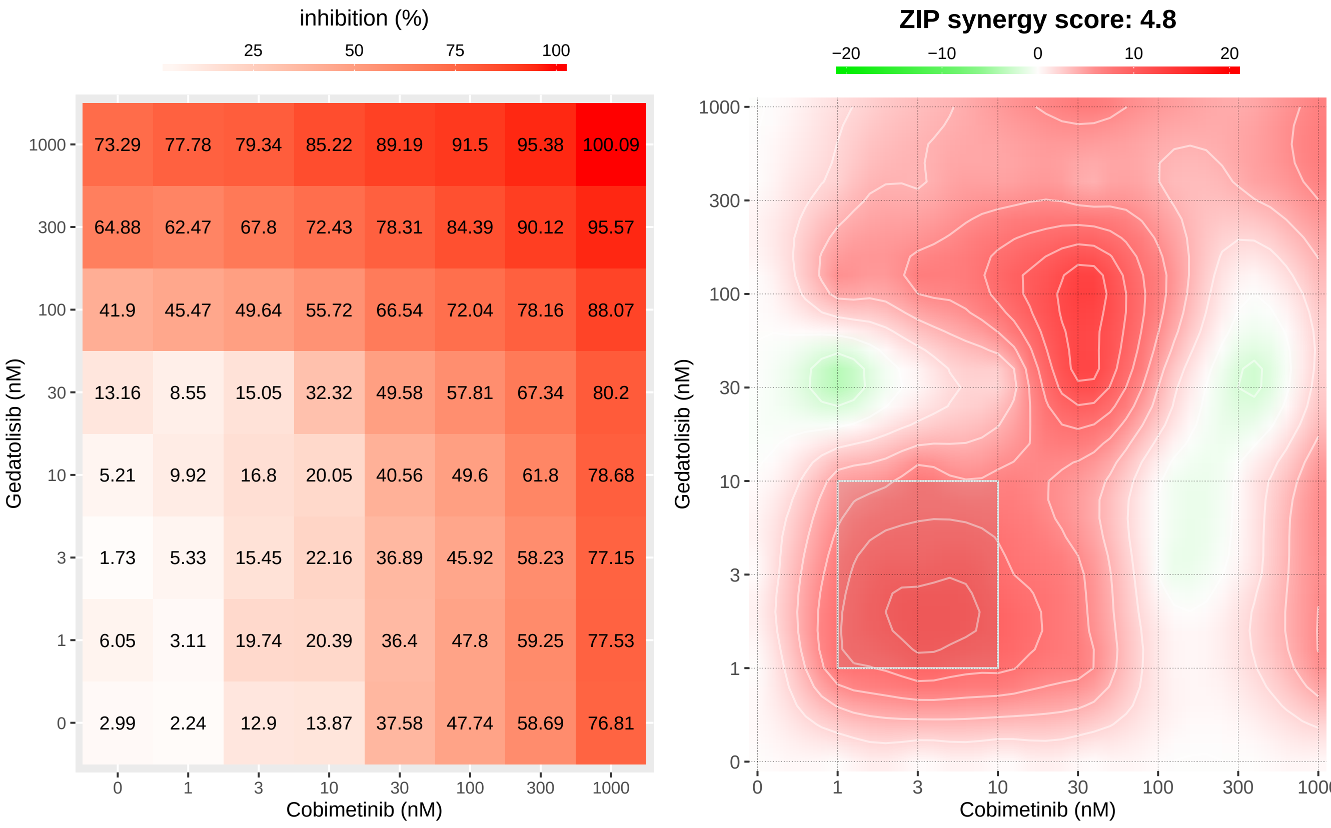

FM-MEL-3

Cobimetinib (nM) & Ganetespib (nM)

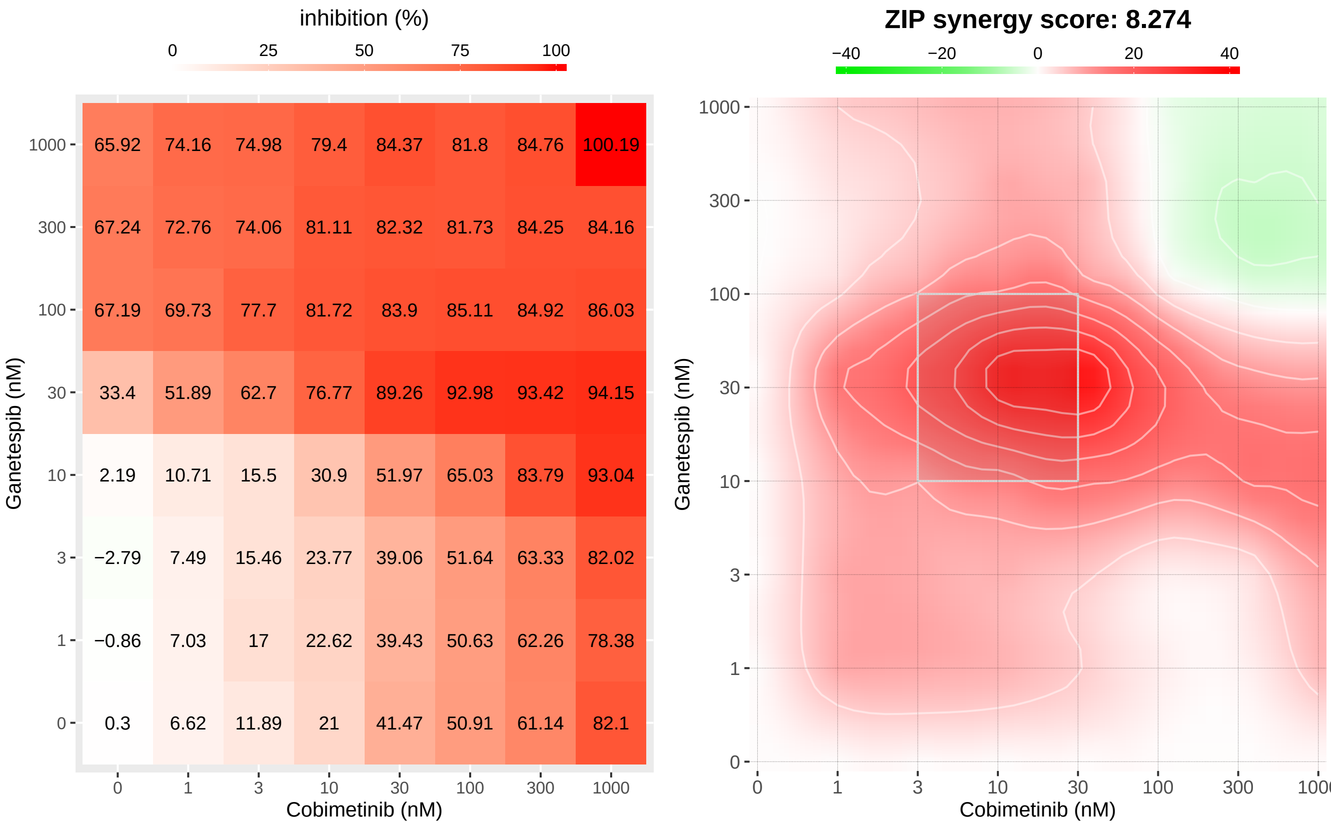

FM-MEL-3

Ulixertinib (nM) & Cobimetinib (nM)

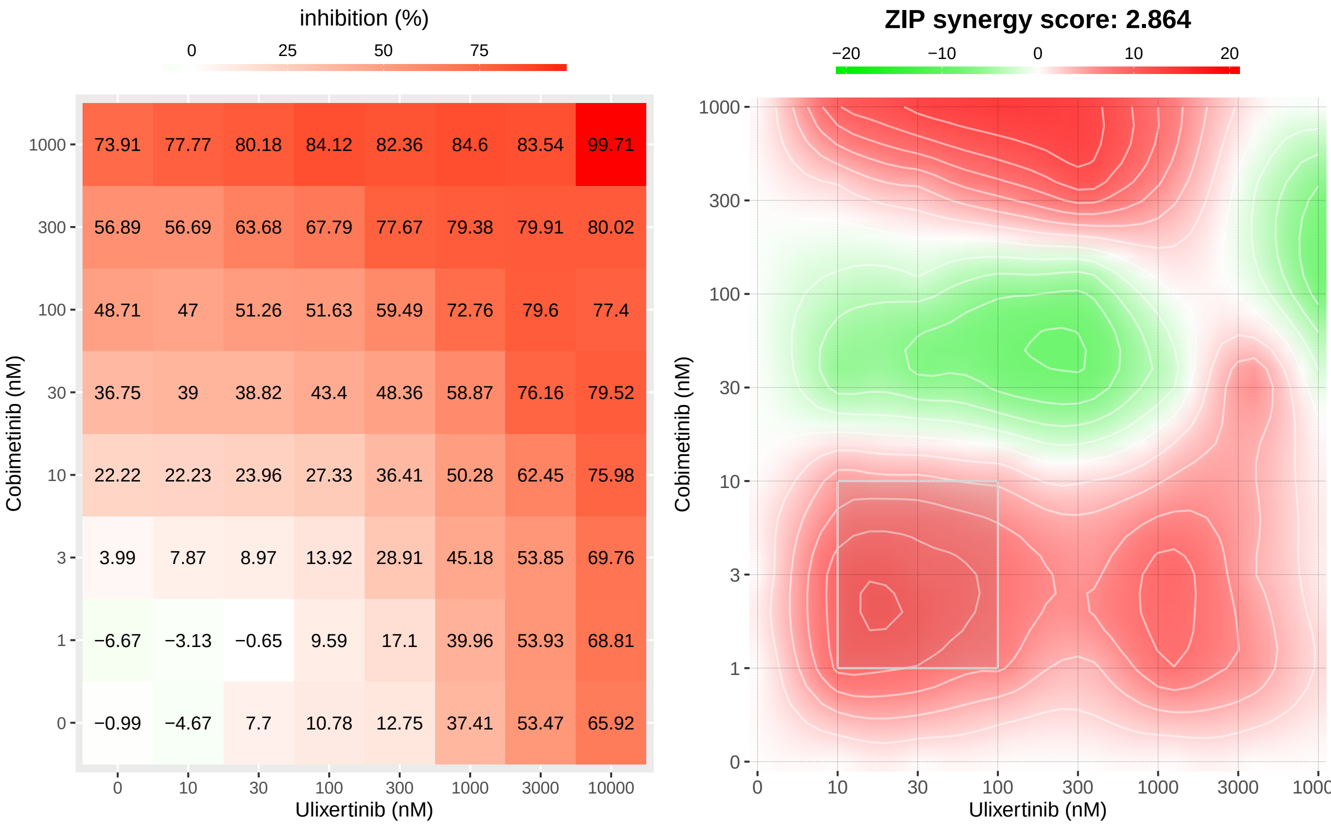

FM-MEL-3

Cobimetinib (nM) & SCH772984 (nM)

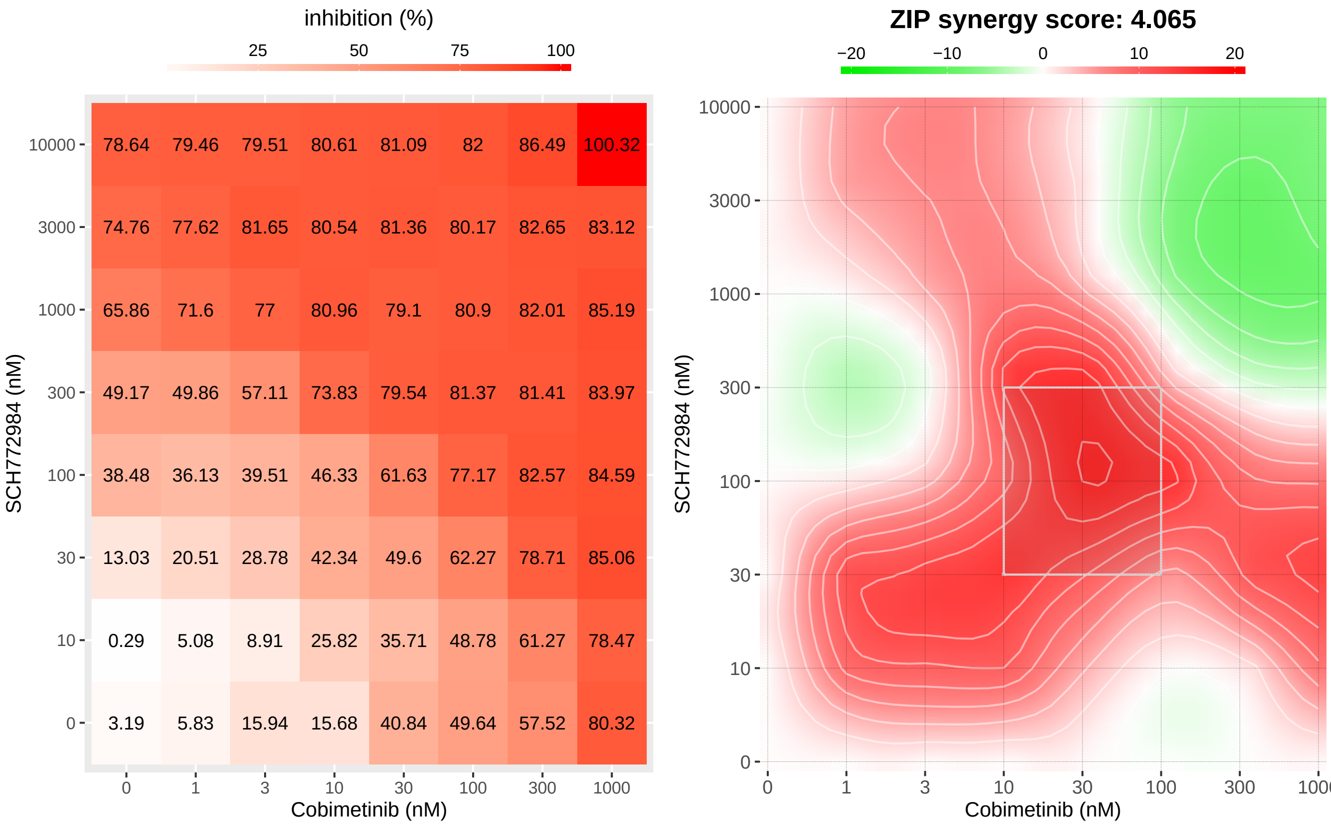

FM-MEL-3

LY3009120 (nM) & Cobimetinib (nM)

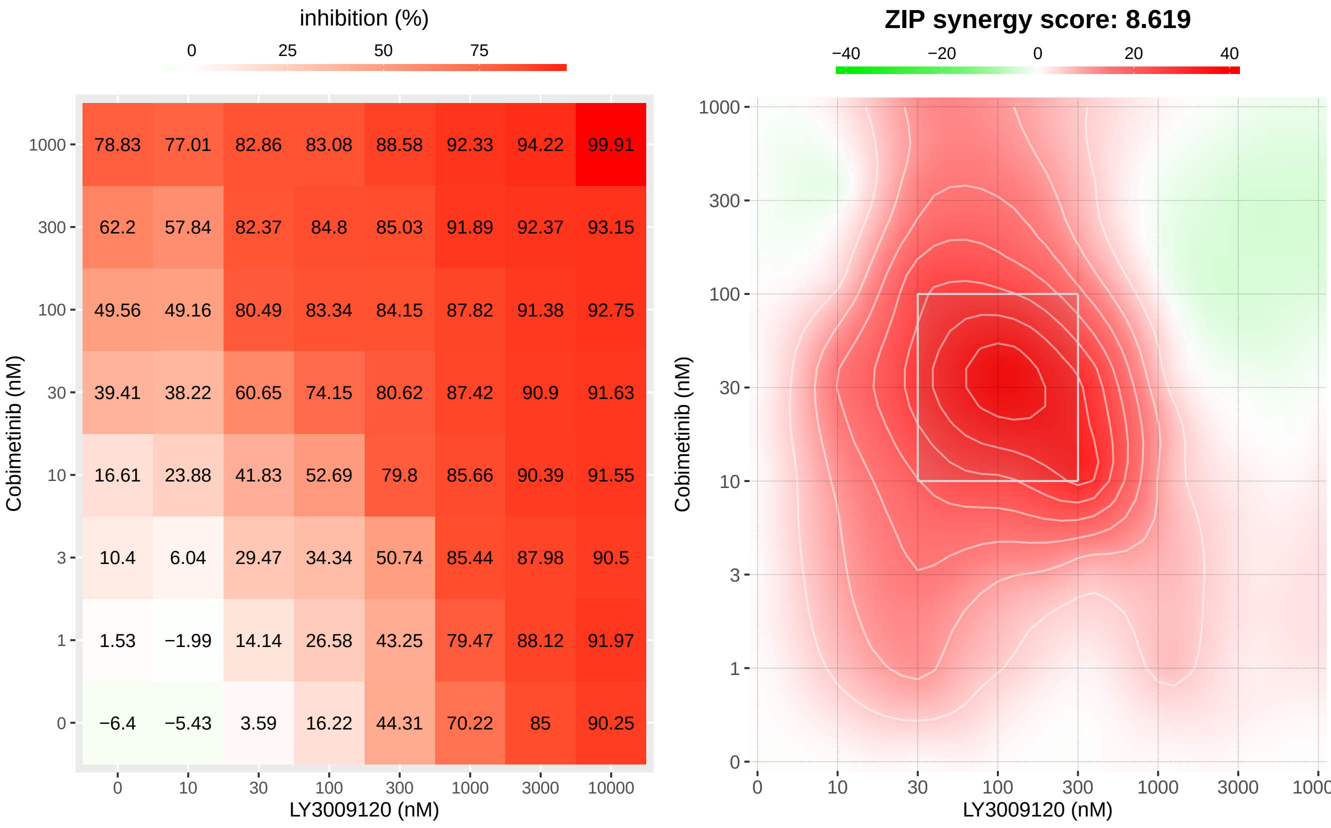

FM-MEL-3

Cobimetinib (nM) & BI 2536 (nM)

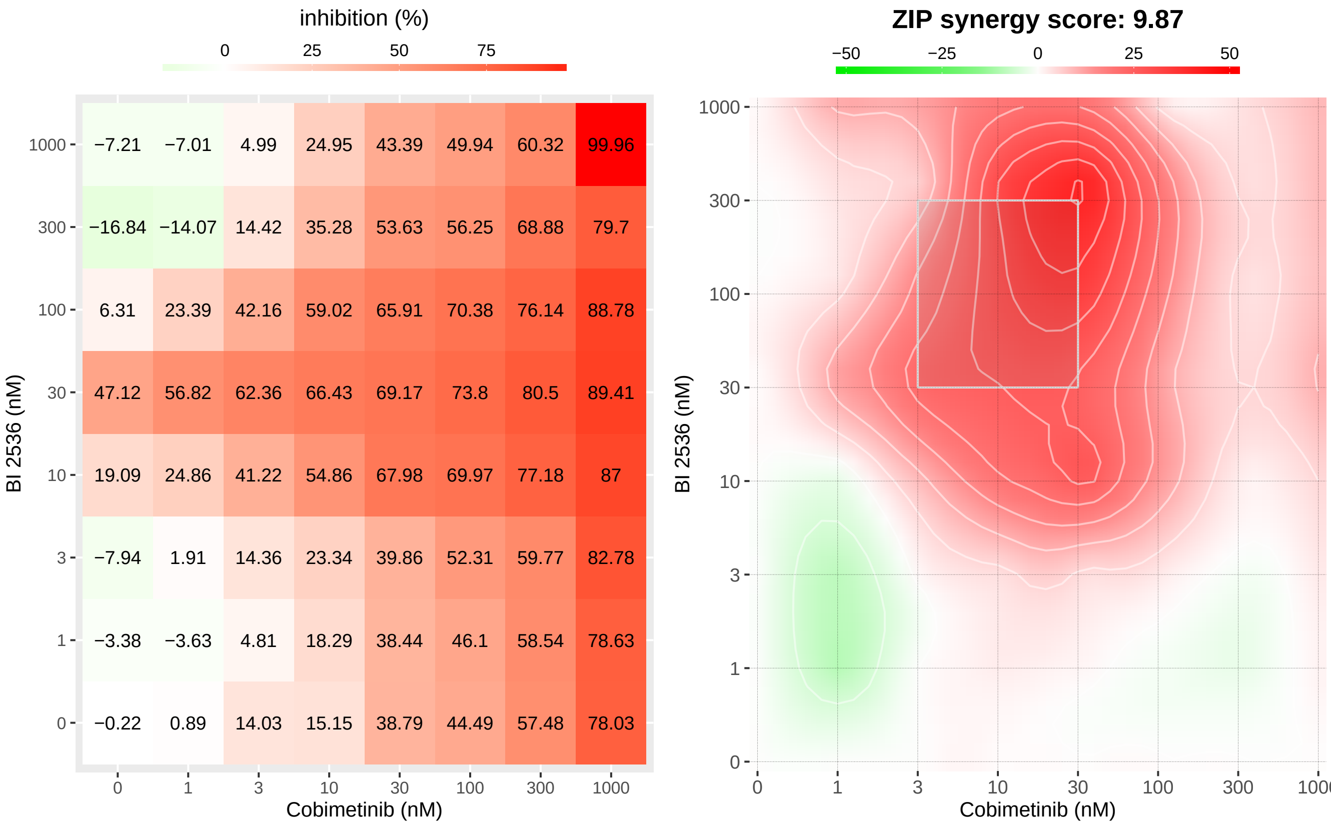

FM-MEL-3

Palbociclib (nM) & Cobimetinib (nM)

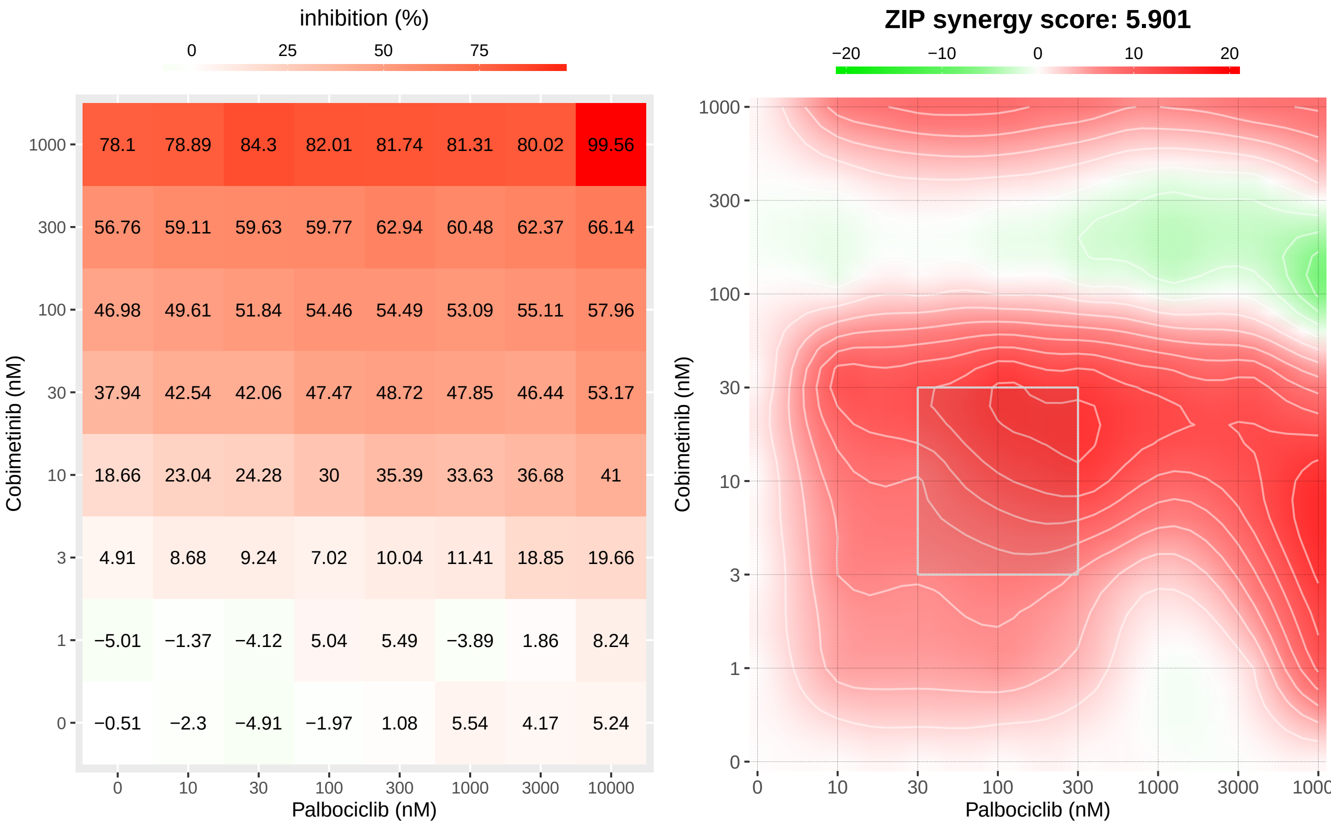

FM-MEL-6

Ponatinib (nM) & Cobimetinib (nM)

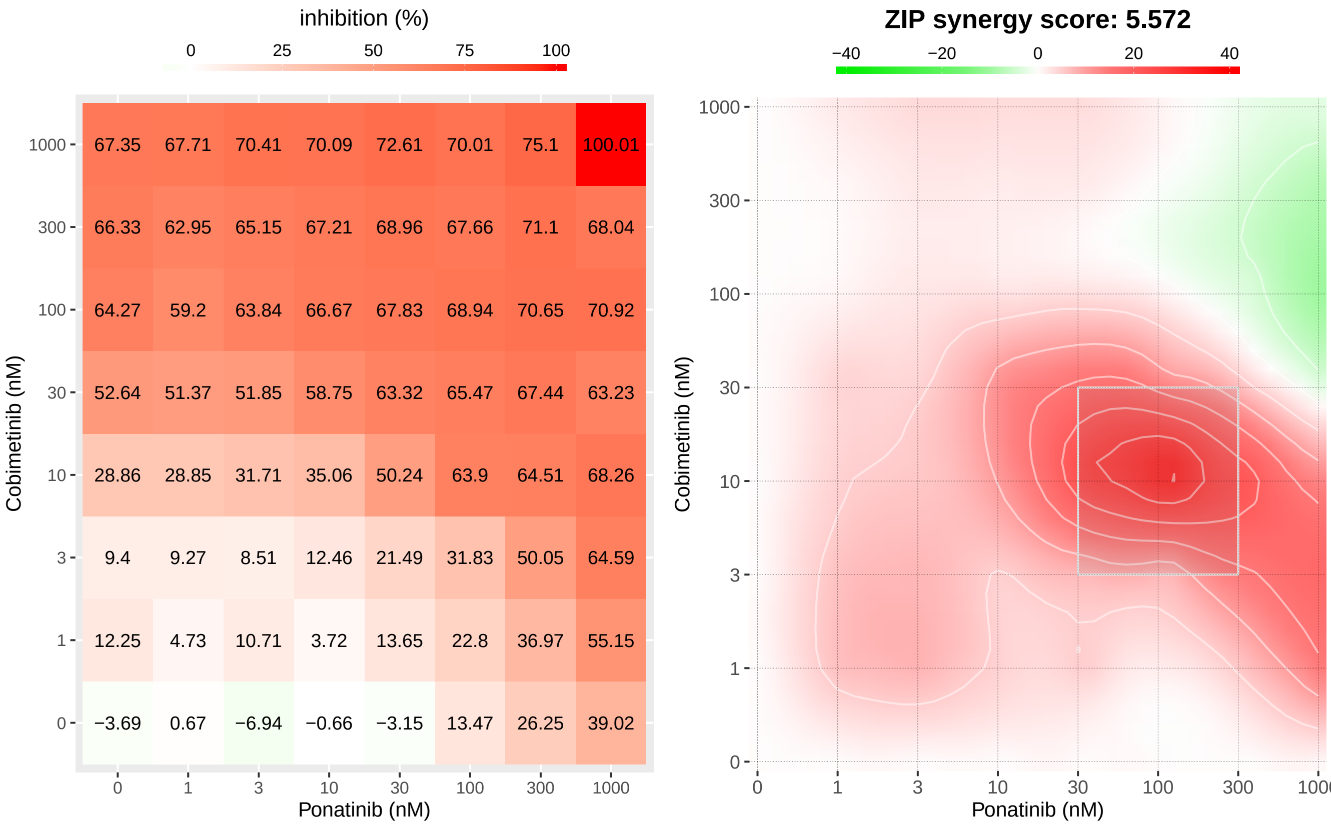

FM-MEL-6

Cobimetinib (nM) & Nilotinib (nM)

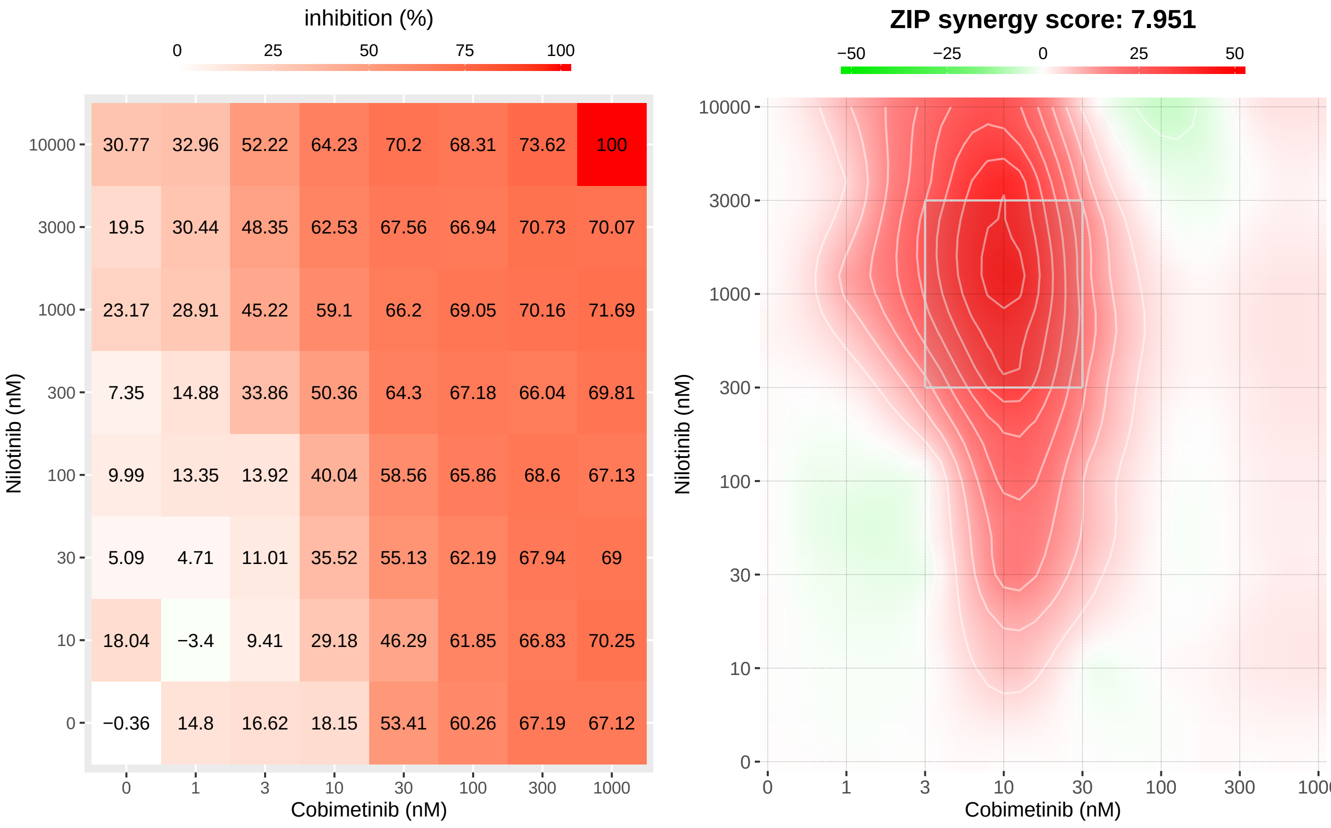

FM-MEL-6

Pictilisib (nM) & Cobimetinib (nM)

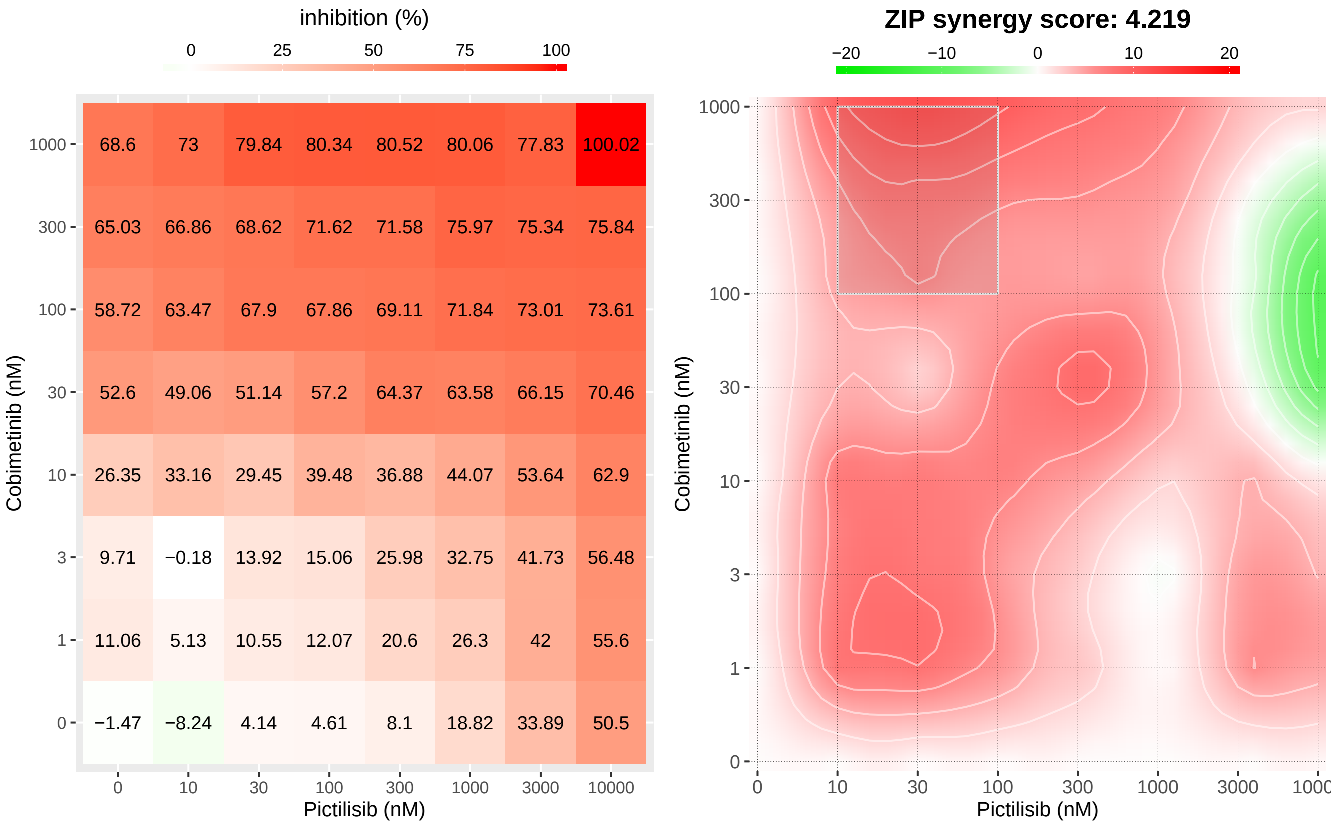

FM-MEL-6

Cobimetinib (nM) & Gedatolisib (nM)

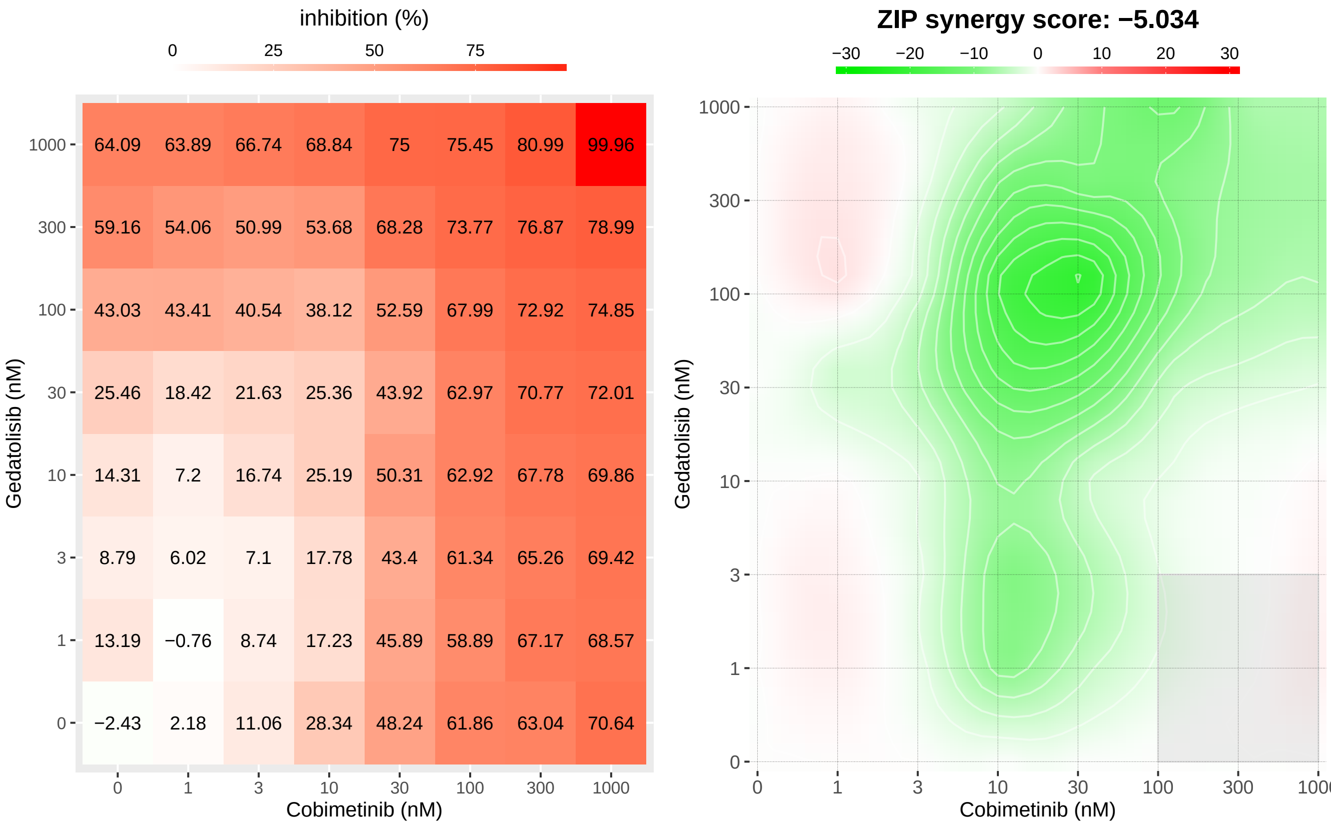

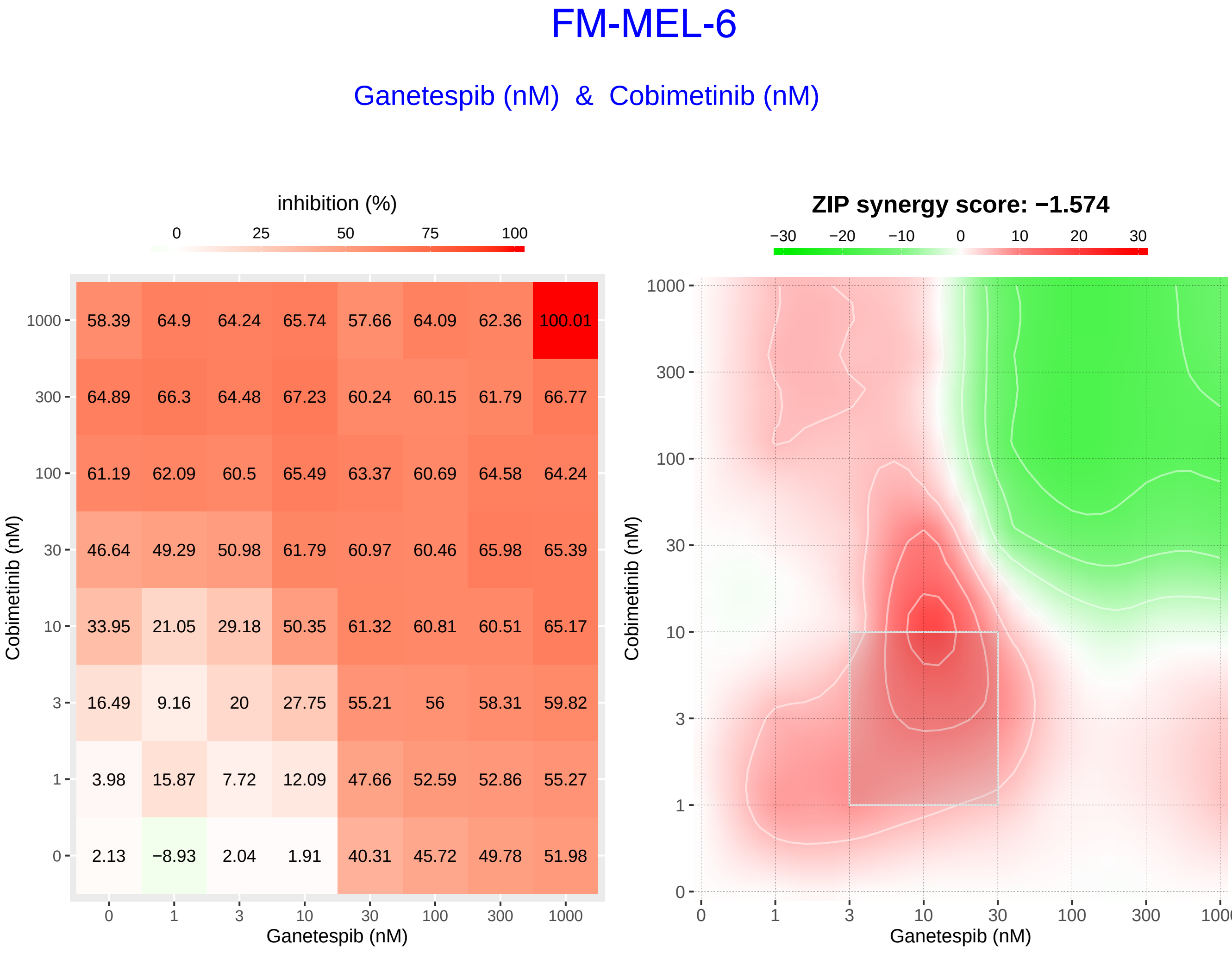
FM-MEL-6
Ganetespib (nM) & Cobimetinib (nM)
inhibition (%)
0 25 50 75 100
1000 58.39 64.9 64.24 65.74 57.66 64.09 62.36 100.01
300 64.89 66.3 64.48 67.23 60.24 60.15 61.79 66.77
100 61.19 62.09 60.5 65.49 63.37 60.69 64.58 64.24
30 46.64 49.29 50.98 61.79 60.97 60.46 65.98 65.39
10 33.95 21.05 29.18 50.35 61.32 60.81 60.51 65.17
3 16.49 9.16 20 27.75 55.21 56 58.31 59.82
1 3.98 15.87 7.72 12.09 47.66 52.59 52.86 55.27
0 2.13 −8.93 2.04 1.91 40.31 45.72 49.78 51.98
0 1 3 10 30 100 300 1000
Ganetespib (nM)
Cobimetinib (nM)
ZIP synergy score: −1.574
−30 −20 −10 0 10 20 30
1000
300
100
30
10
3
1
0
0 1 3 10 30 100 300
Ganetespib (nM)
Cobimetinib (nM)

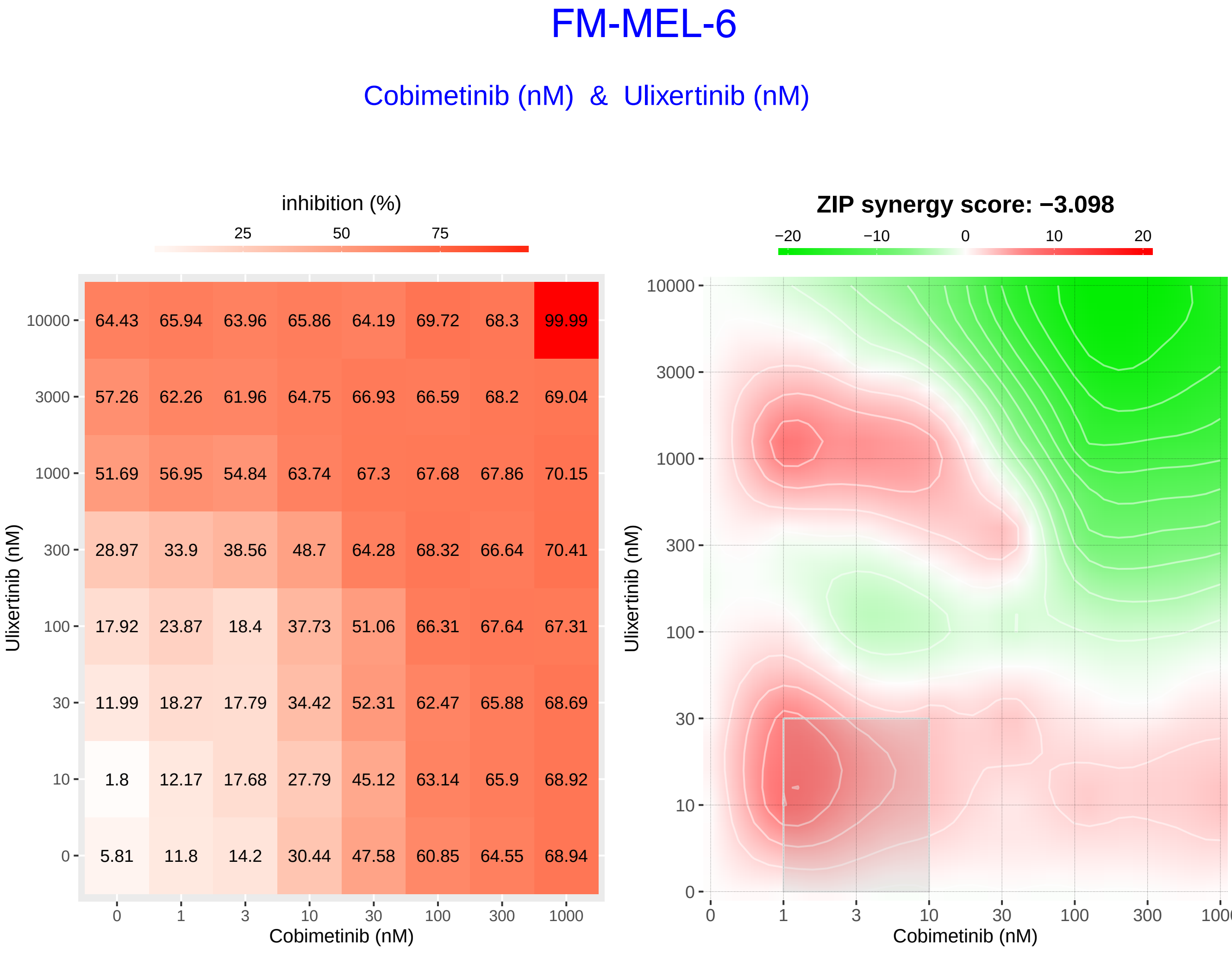
FM-MEL-6
Cobimetinib (nM) & Ulixertinib (nM)
inhibition (%)
25
50
75
ZIP synergy score: −3.098
−20
−10
0
10
20
Ulixertinib (nM)
Cobimetinib (nM)
10000
3000
1000
300
100
30
10
0
1
3
10
30
100
300
1000
64.43 65.94 63.96 65.86 64.19 69.72 68.3 99.99
57.26 62.26 61.96 64.75 66.93 66.59 68.2 69.04
51.69 56.95 54.84 63.74 67.3 67.68 67.86 70.15
28.97 33.9 38.56 48.7 64.28 68.32 66.64 70.41
17.92 23.87 18.4 37.73 51.06 66.31 67.64 67.31
11.99 18.27 17.79 34.42 52.31 62.47 65.88 68.69
1.8 12.17 17.68 27.79 45.12 63.14 65.9 68.92
5.81 11.8 14.2 30.44 47.58 60.85 64.55 68.94

FM-MEL-6

SCH772984 (nM) & Cobimetinib (nM)

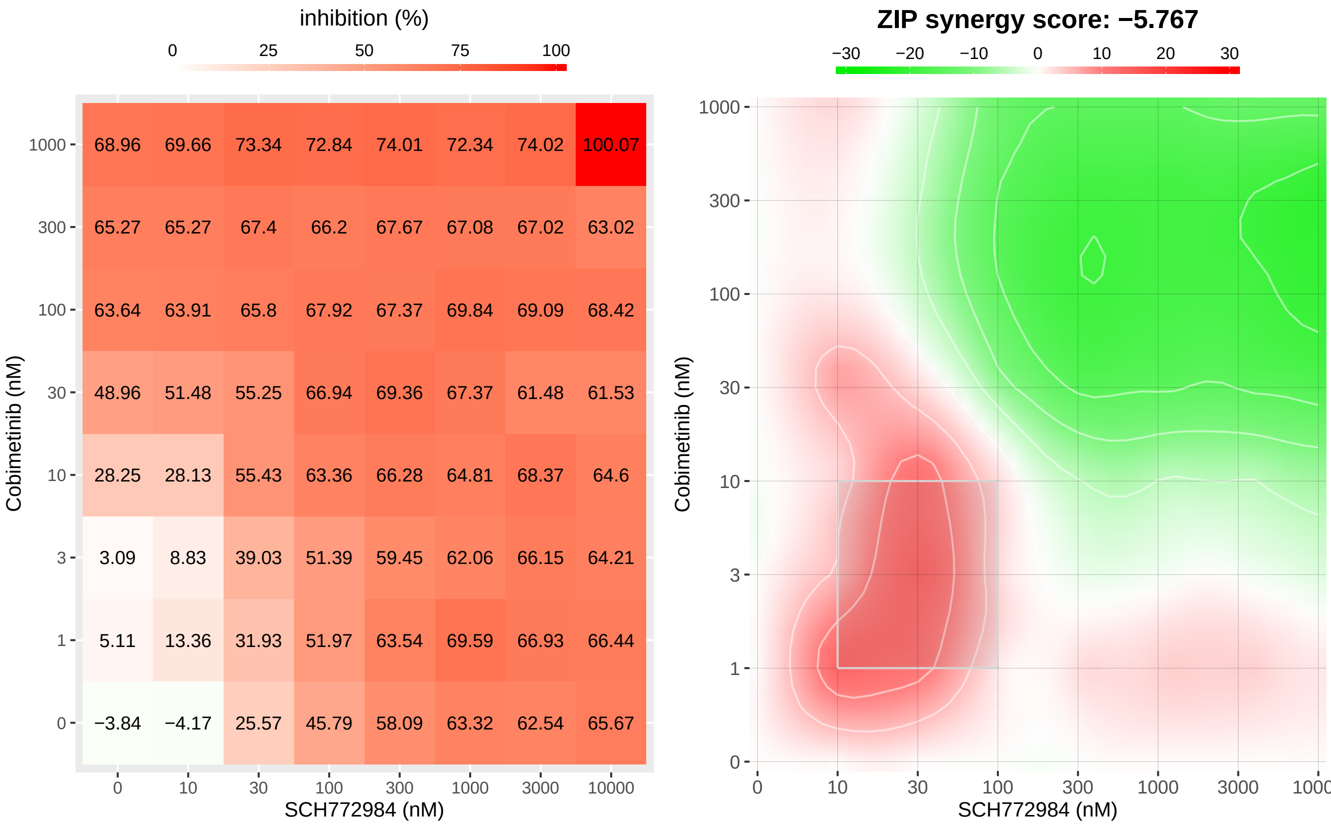

FM-MEL-6

Cobimetinib (nM) & LY3009120 (nM)

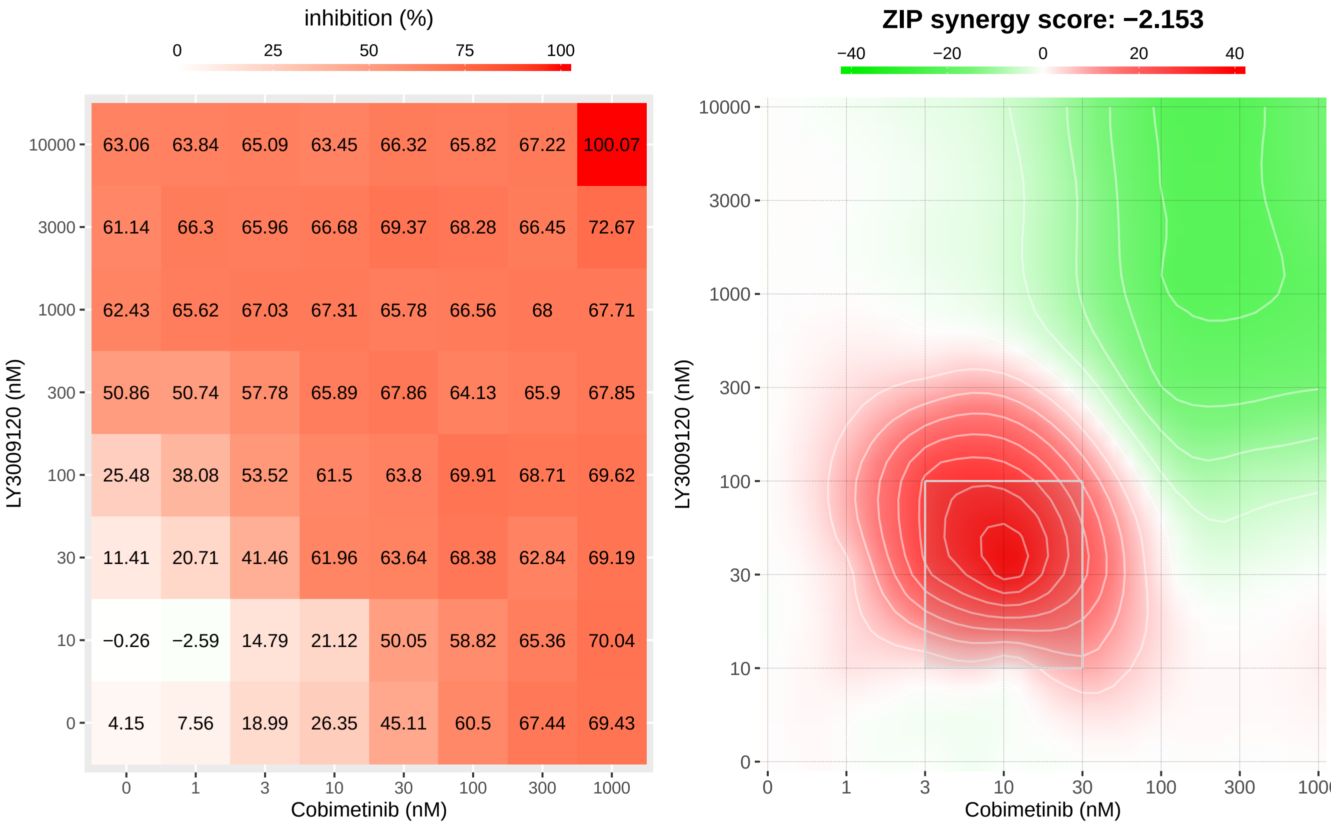

FM-MEL-6

Cobimetinib (nM) & BI 2536 (nM)

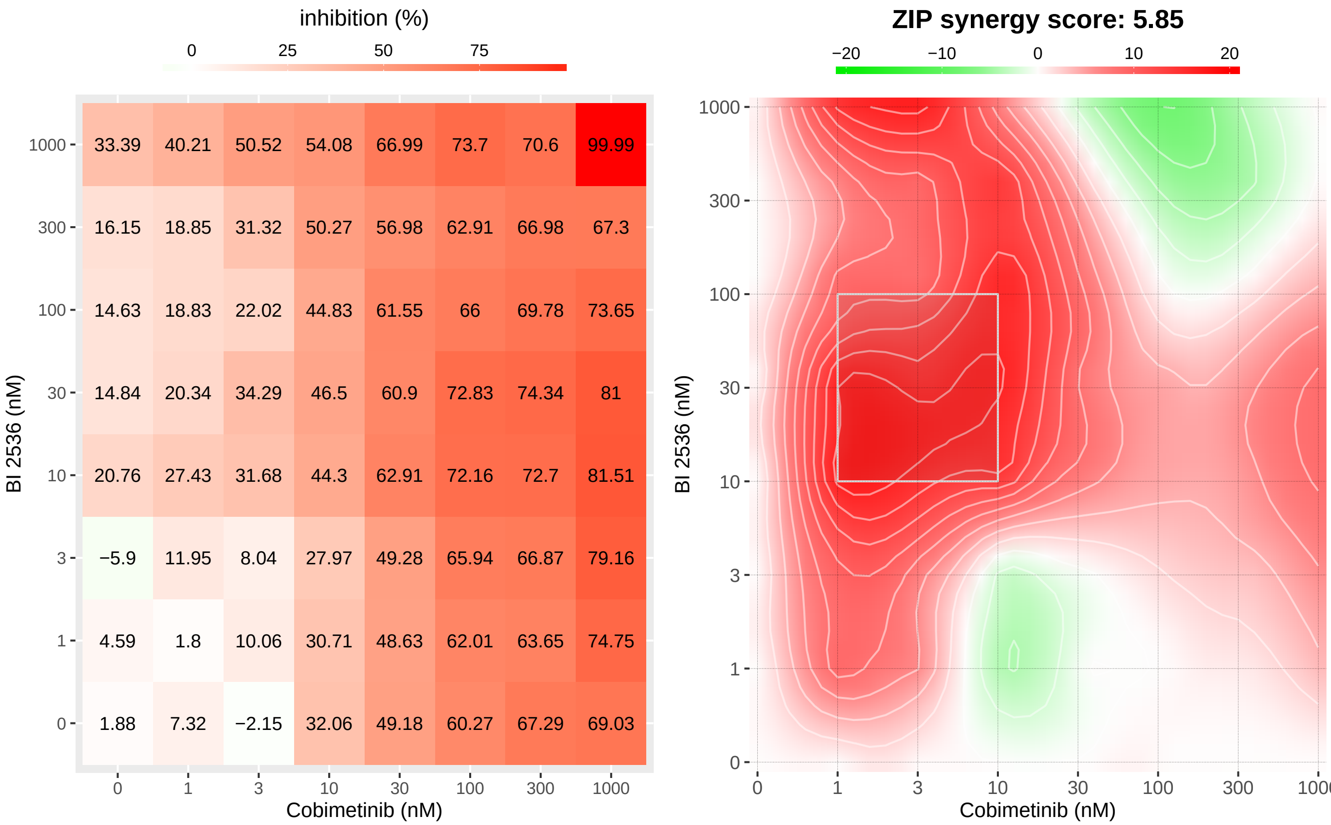

FM-MEL-6

Cobimetinib (nM) & Palbociclib (nM)

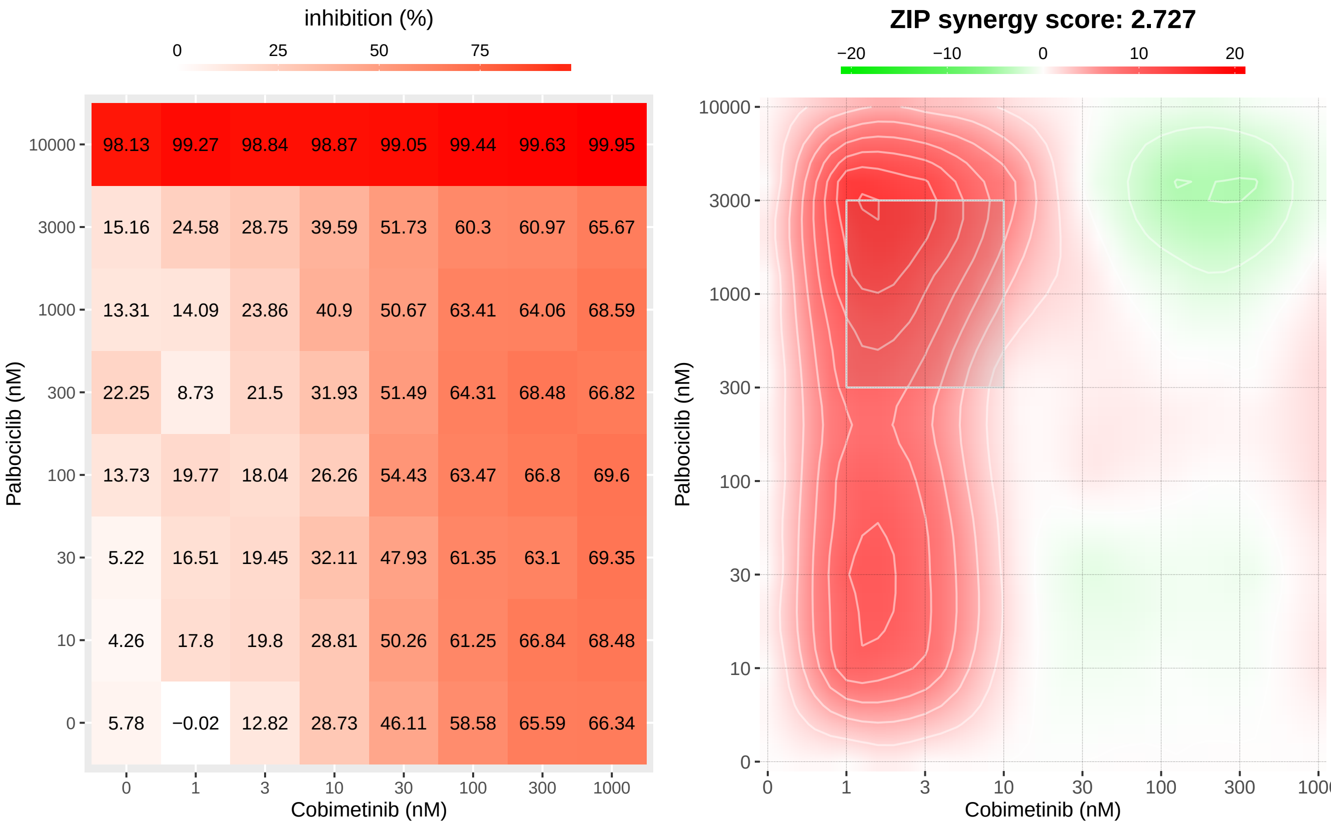

WM852

Ponatinib (nM) & Cobimetinib (nM)

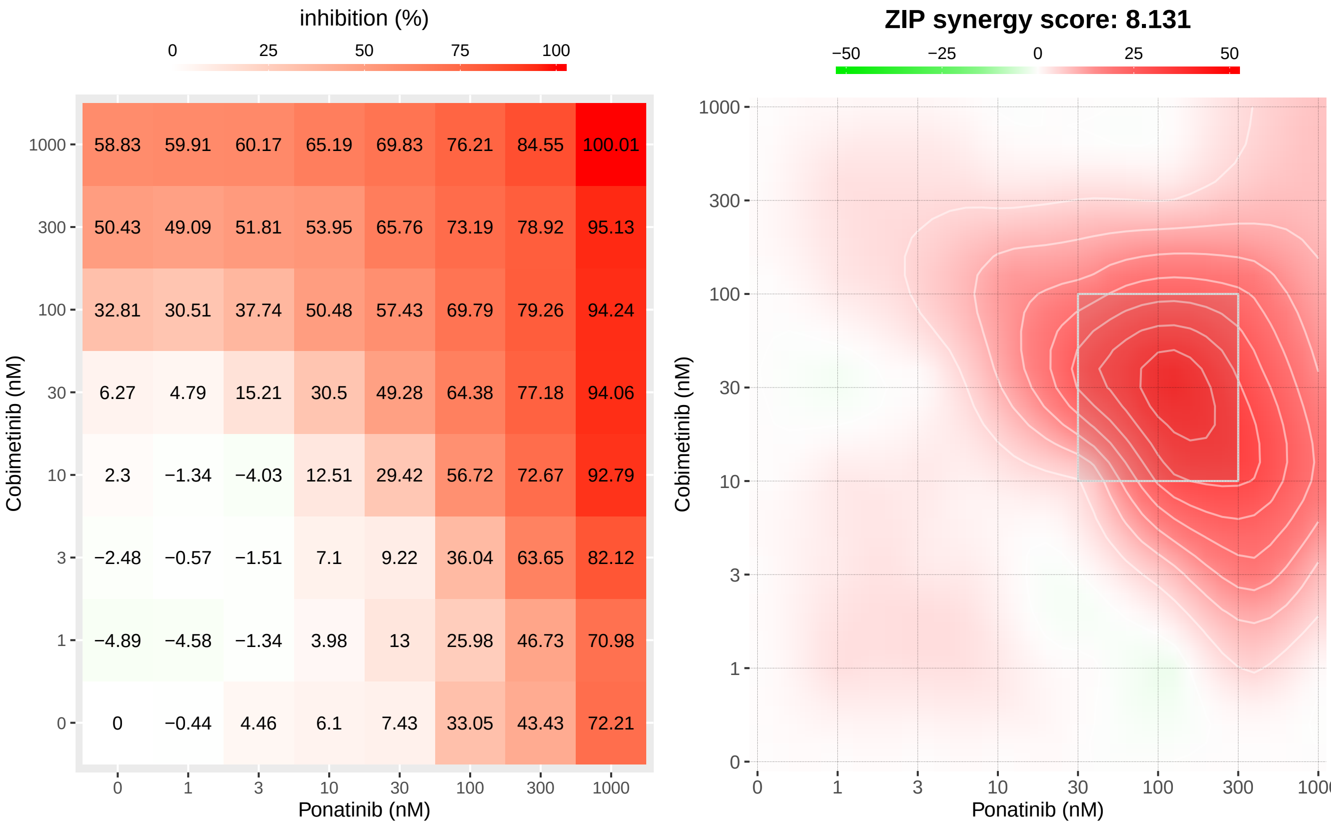

WM852

Cobimetinib (nM) & Nilotinib (nM)

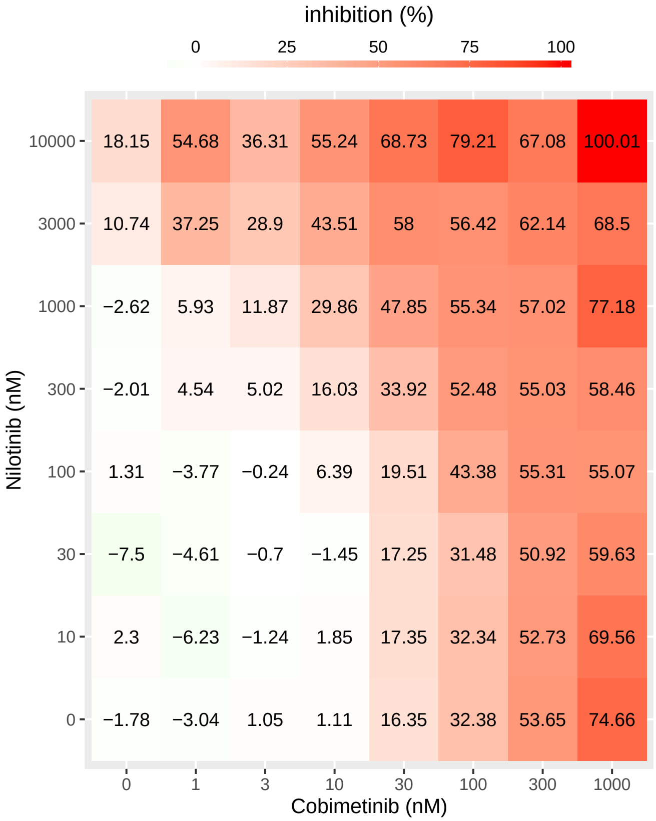

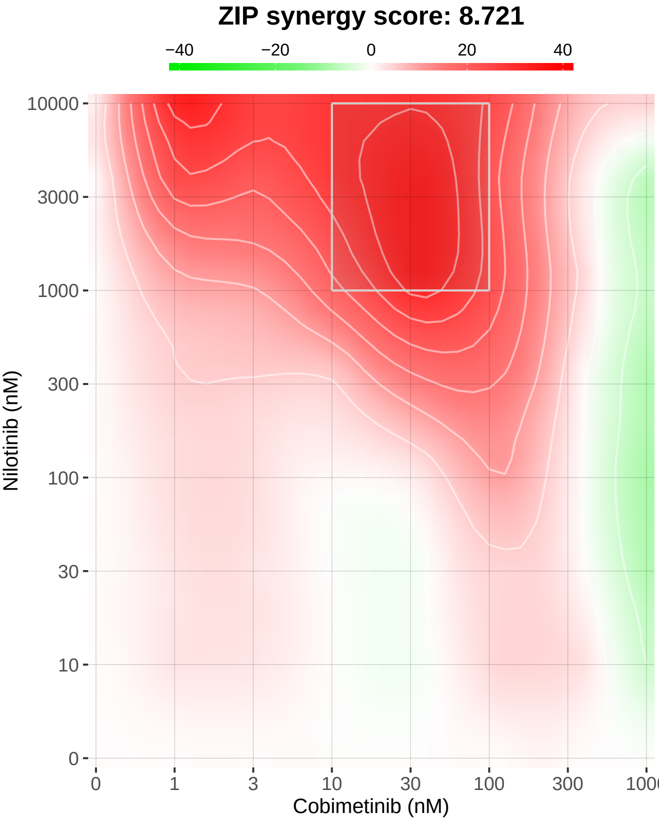

WM852

Pictilisib (nM) & Cobimetinib (nM)

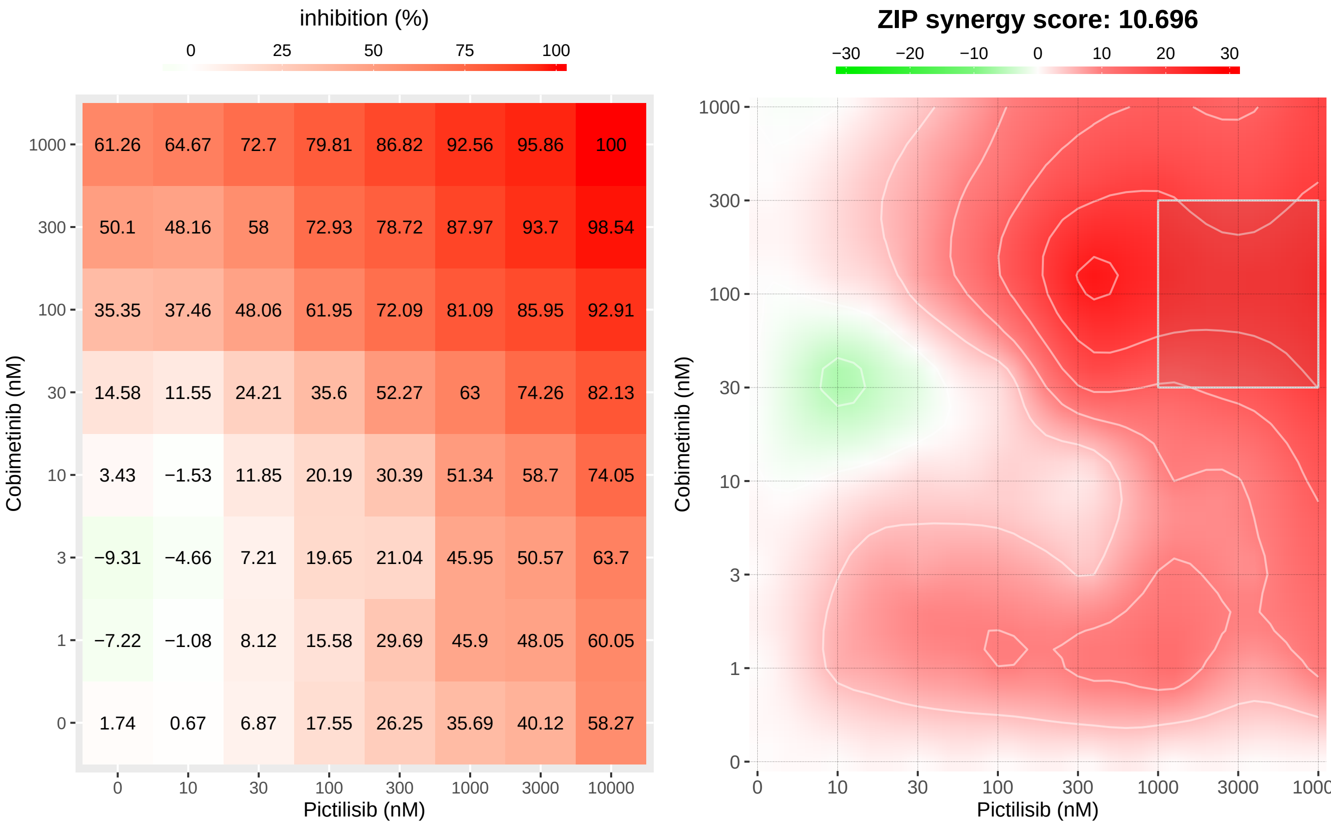

WM852

Cobimetinib (nM) & Gedatolisib (nM)

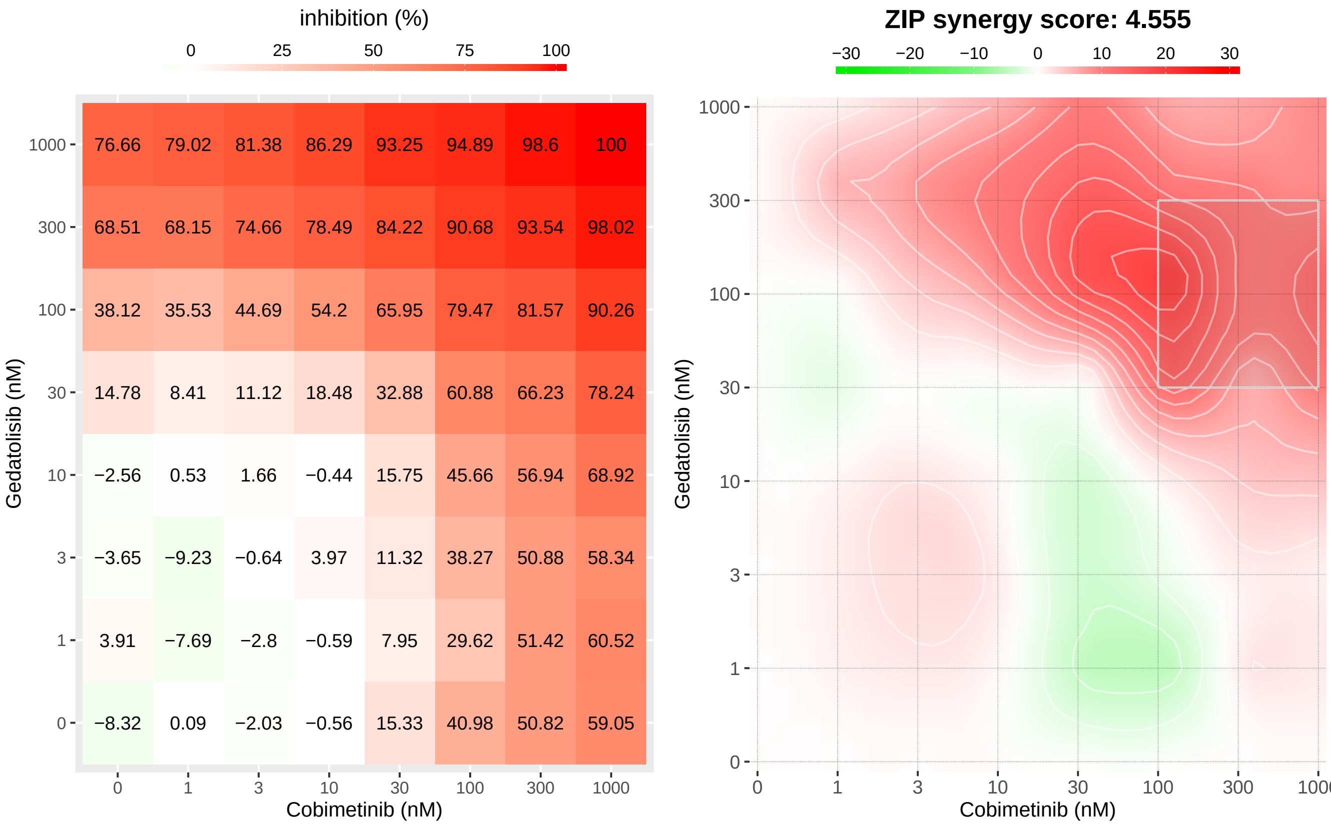

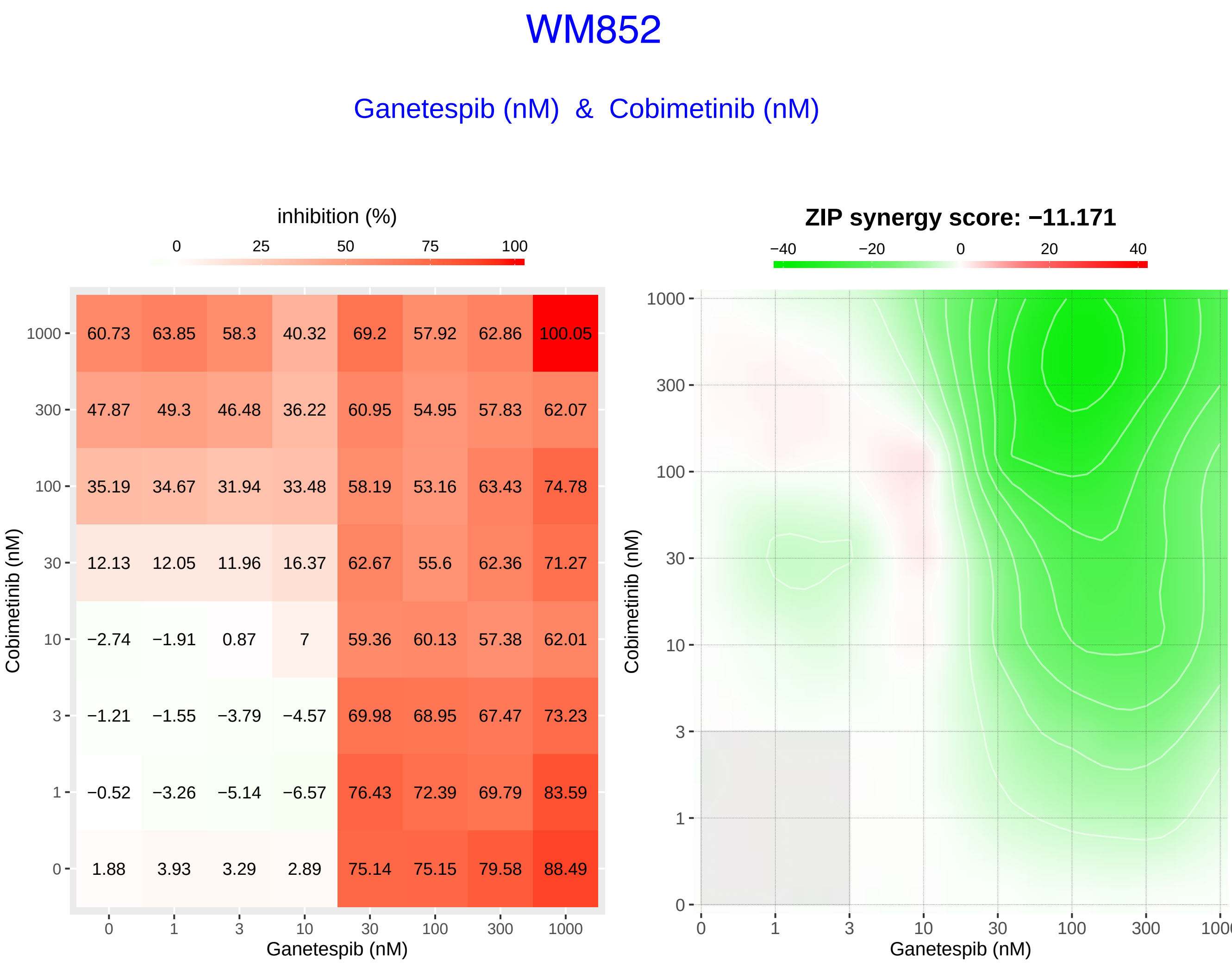
WM852
Ganetespib (nM) & Cobimetinib (nM)
inhibition (%)
0 25 50 75 100
Cobimetinib (nM)
1000 60.73 63.85 58.3 40.32 69.2 57.92 62.86 100.05
300 47.87 49.3 46.48 36.22 60.95 54.95 57.83 62.07
100 35.19 34.67 31.94 33.48 58.19 53.16 63.43 74.78
30 12.13 12.05 11.96 16.37 62.67 55.6 62.36 71.27
10 −2.74 −1.91 0.87 7 59.36 60.13 57.38 62.01
3 −1.21 −1.55 −3.79 −4.57 69.98 68.95 67.47 73.23
1 −0.52 −3.26 −5.14 −6.57 76.43 72.39 69.79 83.59
0 1.88 3.93 3.29 2.89 75.14 75.15 79.58 88.49
0 1 3 10 30 100 300 1000
Ganetespib (nM)
ZIP synergy score: −11.171
−40 −20 0 20 40
Cobimetinib (nM)
1000 300 100 30 10 3 1 0
0 1 3 10 30 100 300 1000
Ganetespib (nM)

WM852

Cobimetinib (nM) & Ulixertinib (nM)

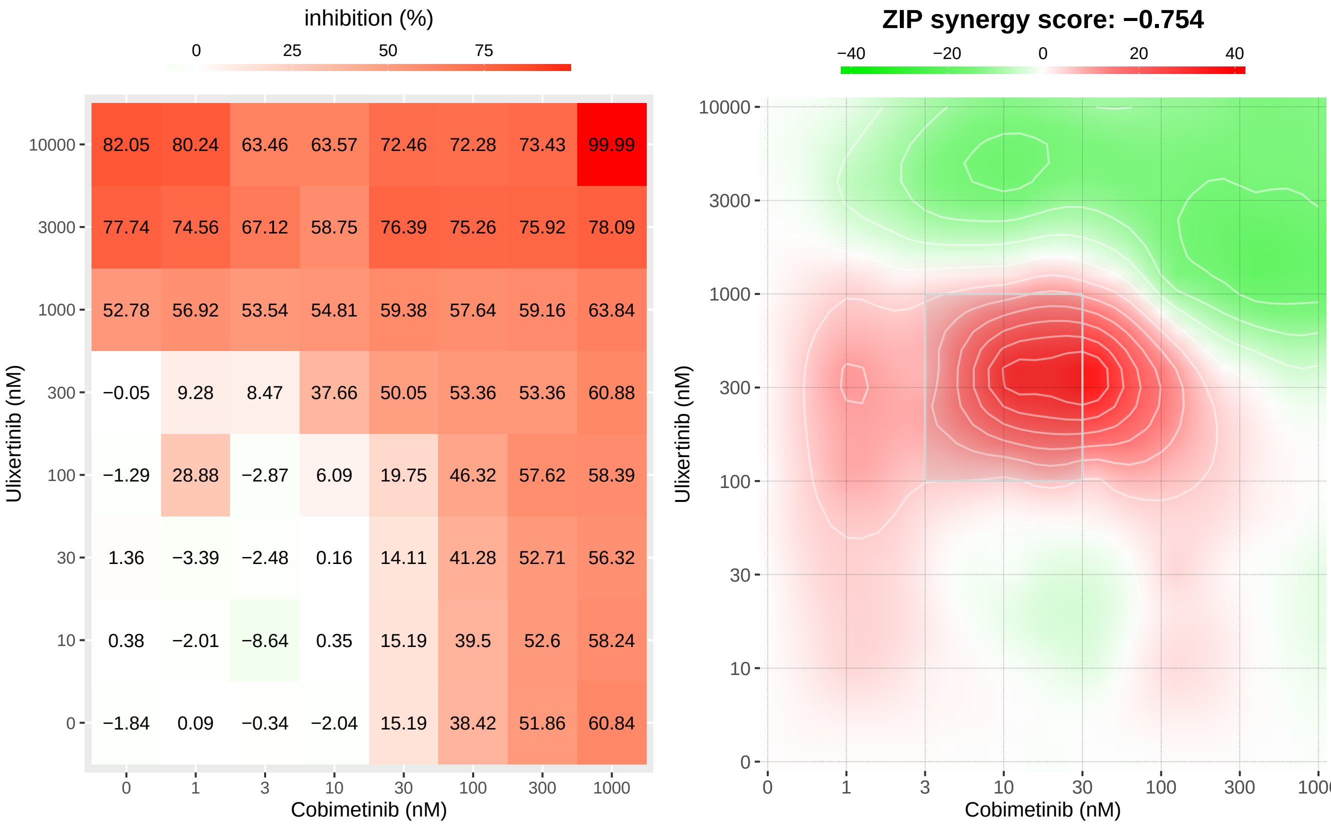

WM852

SCH772984 (nM) & Cobimetinib (nM)

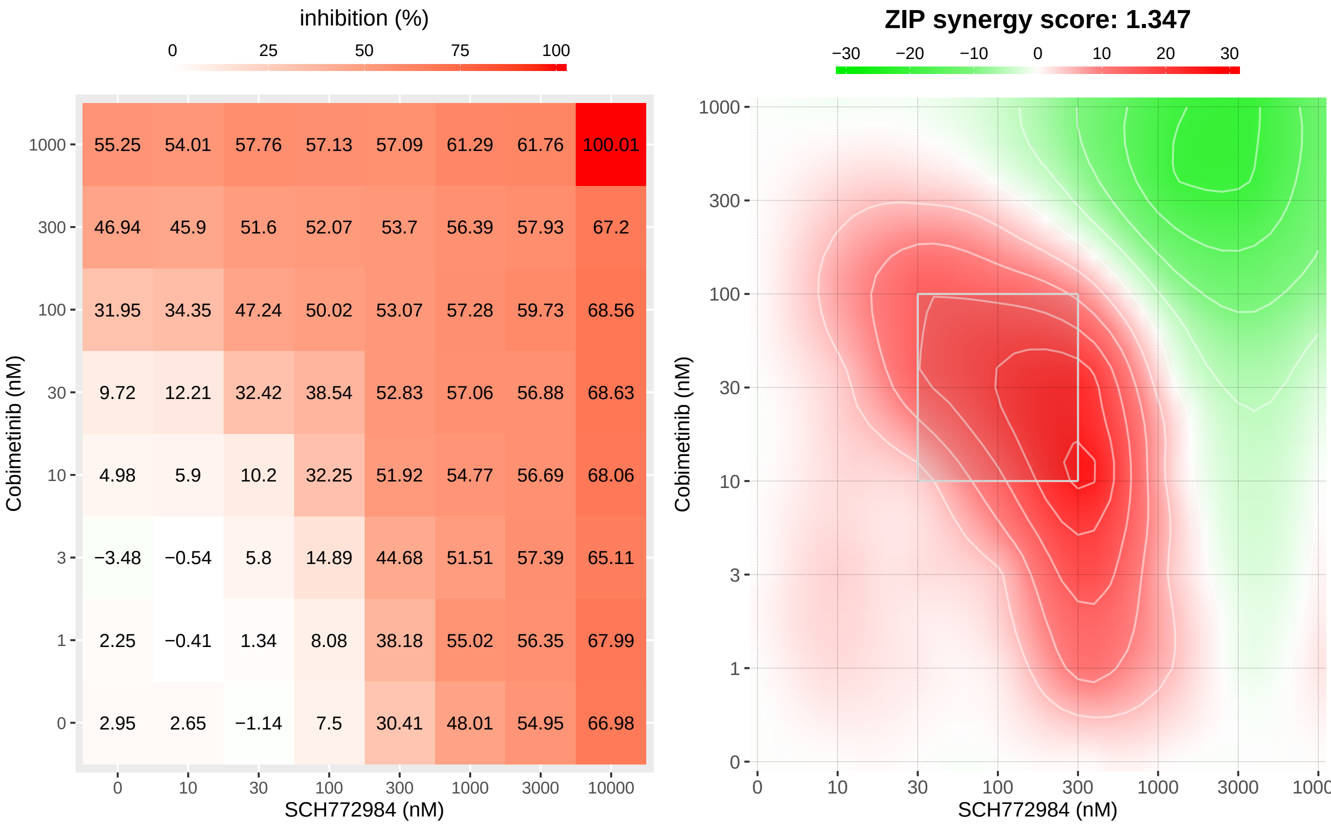

WM852

Cobimetinib (nM) & LY3009120 (nM)

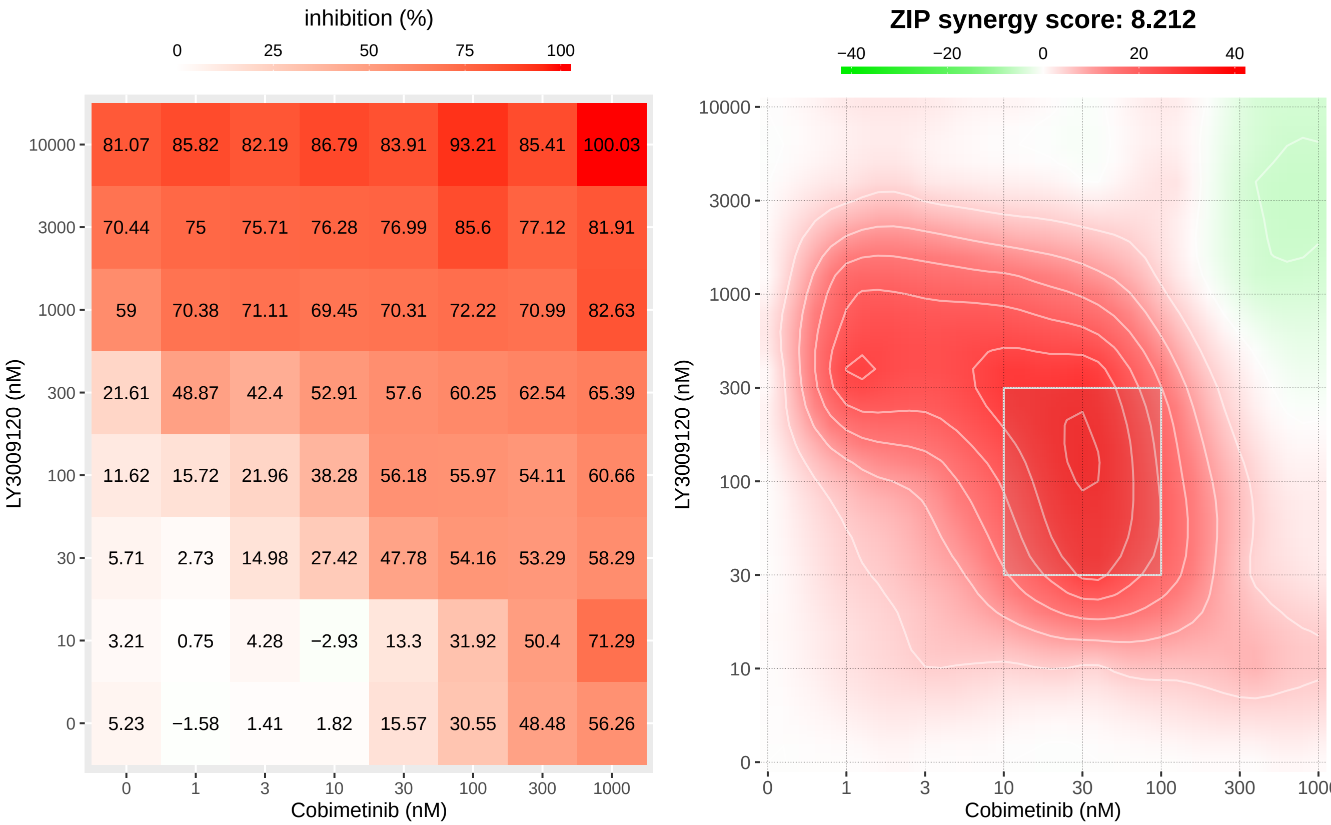

WM852

Cobimetinib (nM) & BI 2536 (nM)

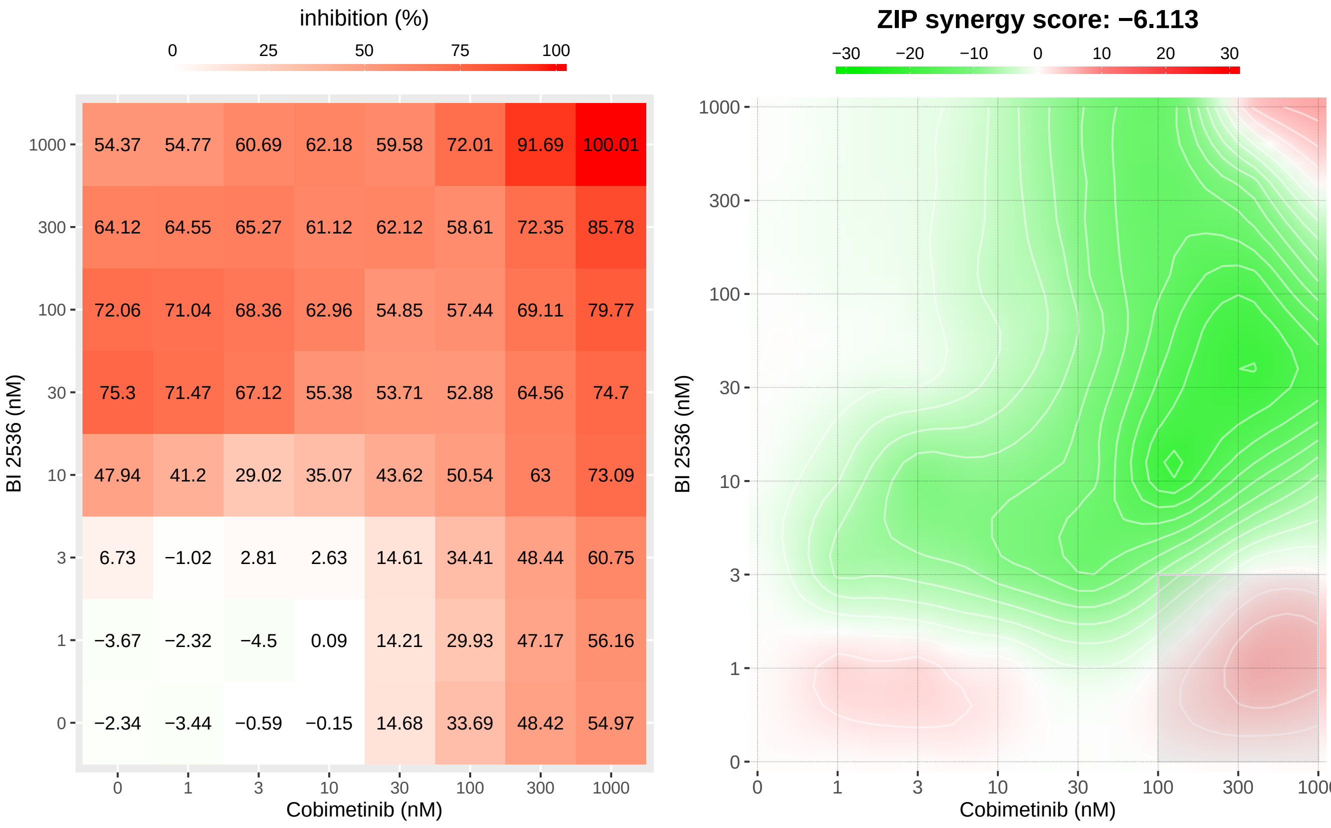

WM852

Cobimetinib (nM) & Palbociclib (nM)

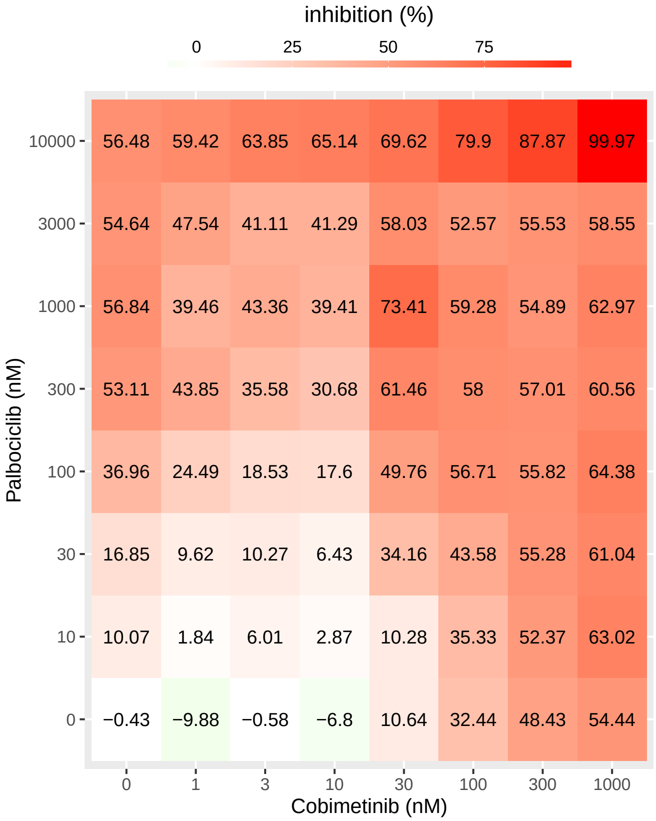

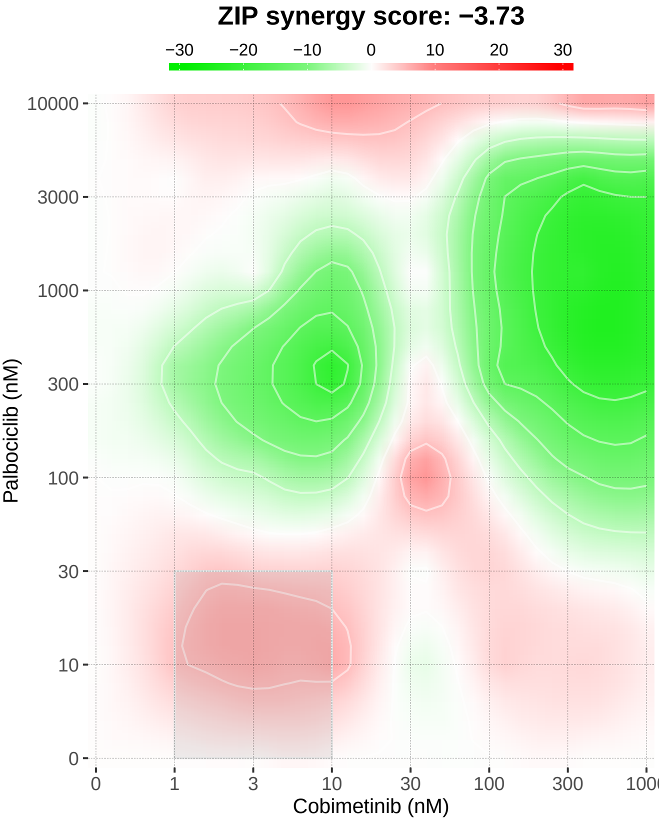

BOWES

Ponatinib (nM) & Cobimetinib (nM)

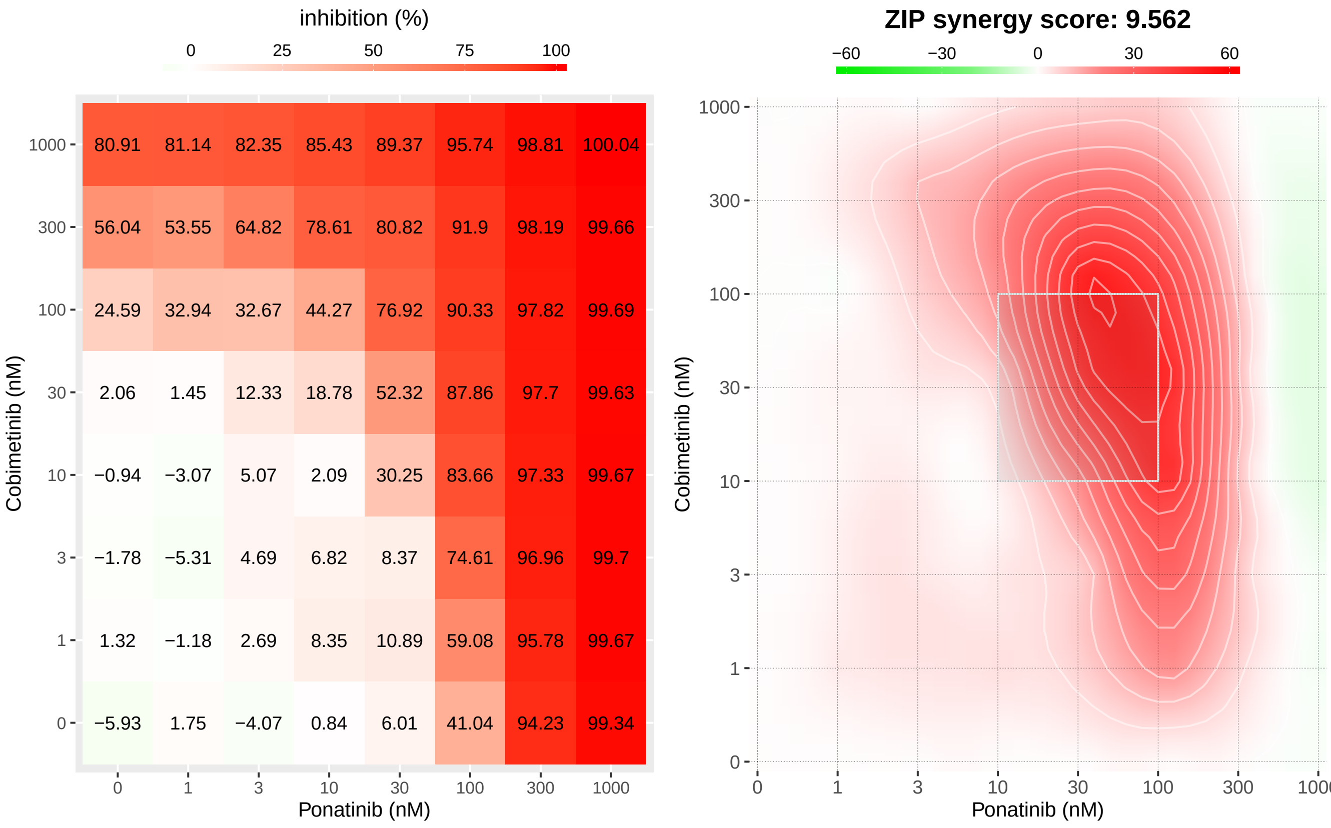

BOWES

Cobimetinib (nM) & Nilotinib (nM)

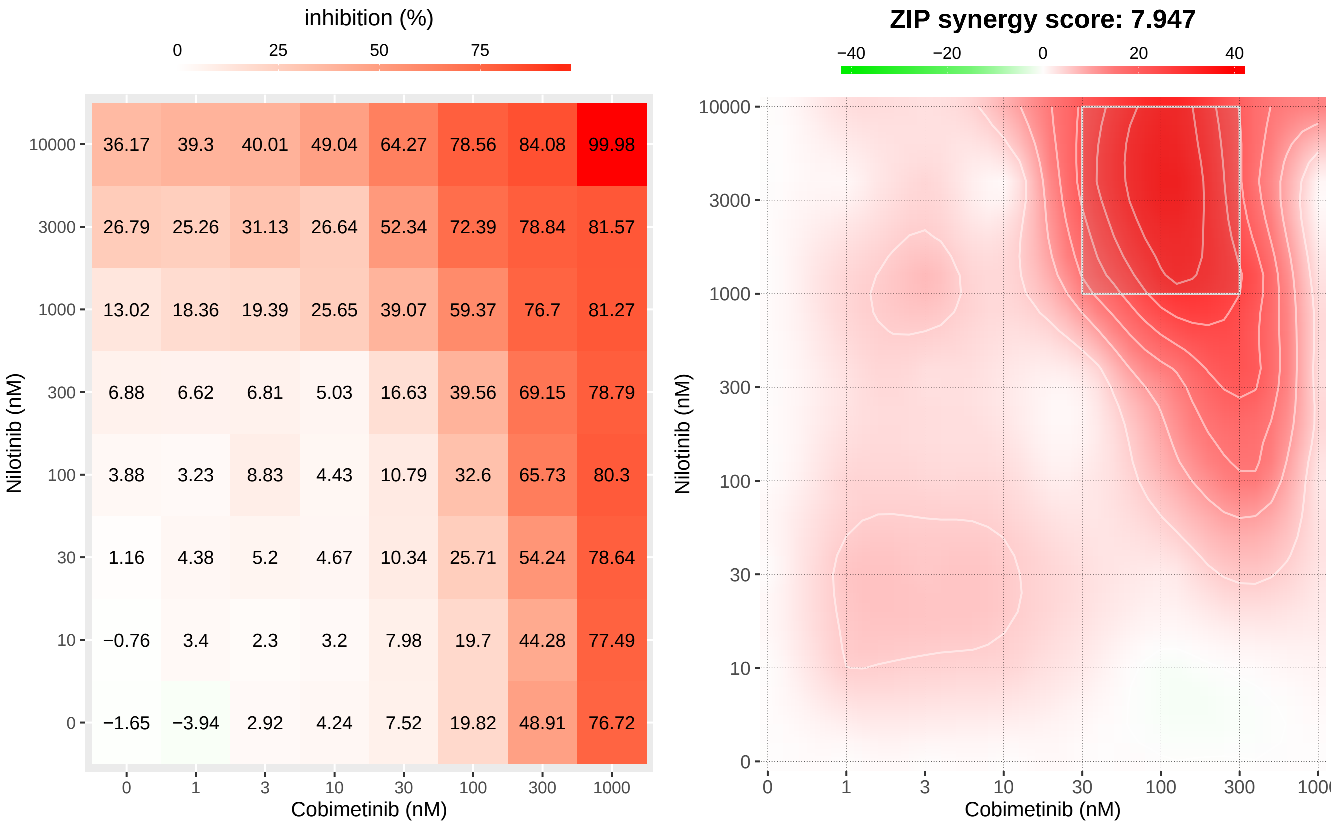

BOWES

Pictilisib (nM) & Cobimetinib (nM)

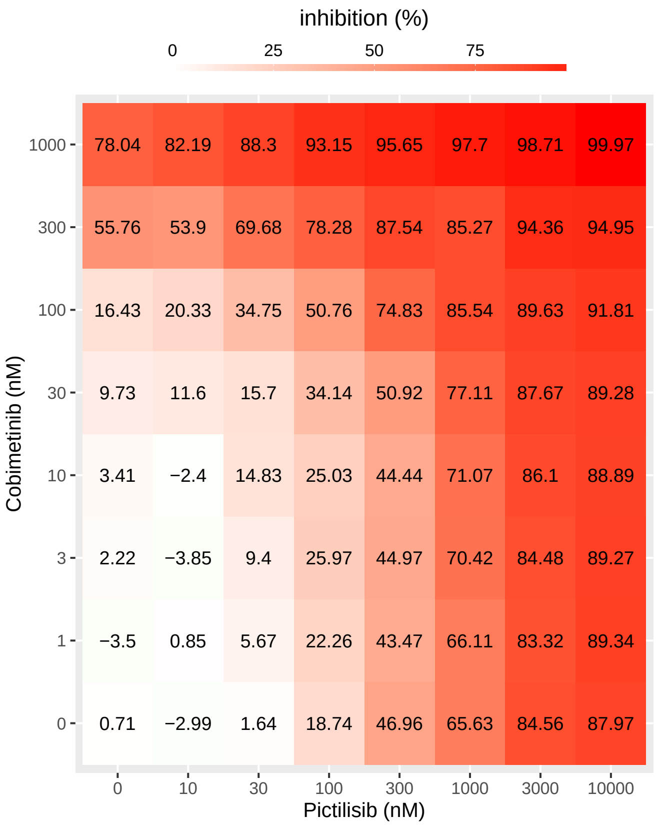

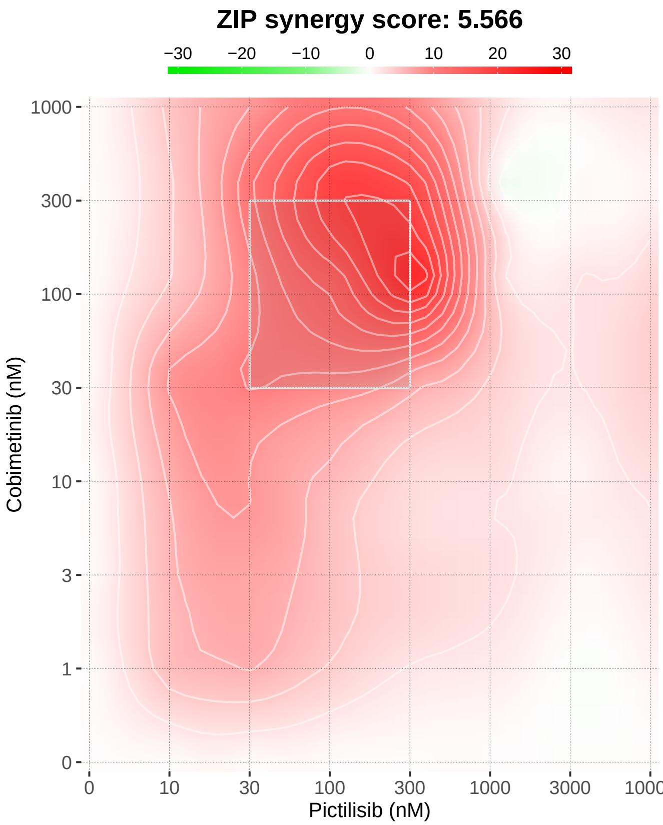

BOWES

Cobimetinib (nM) & Gedatolisib (nM)

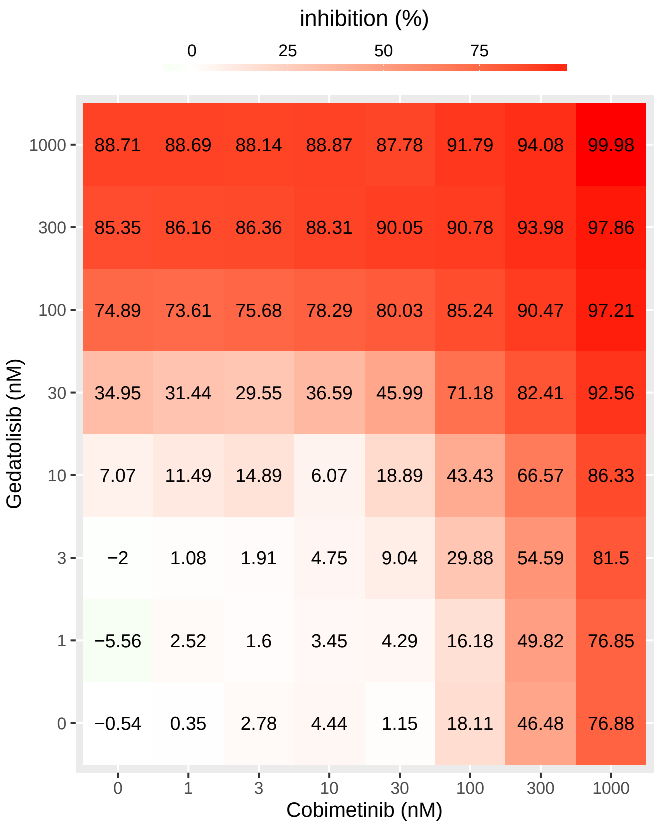

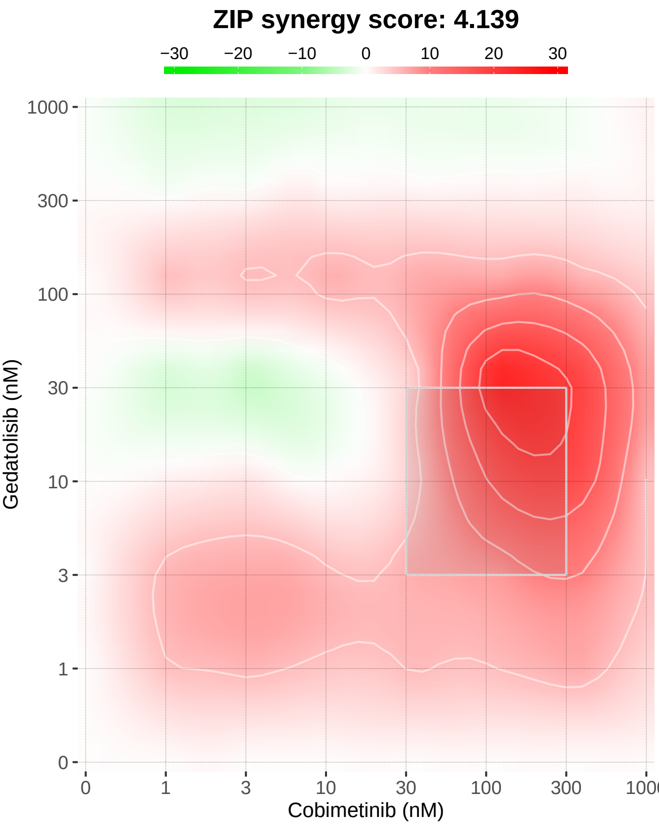

BOWES

Ganetespib (nM) & Cobimetinib (nM)

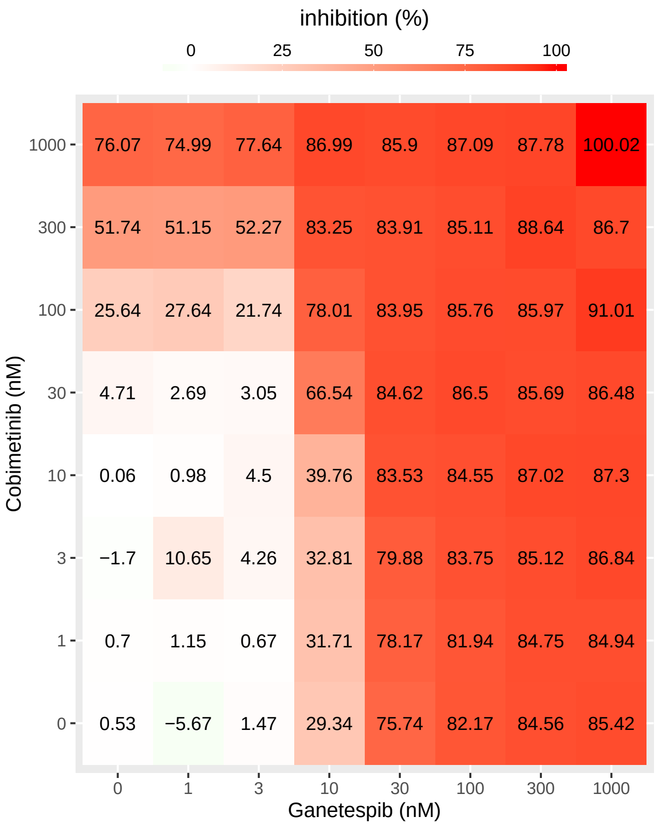

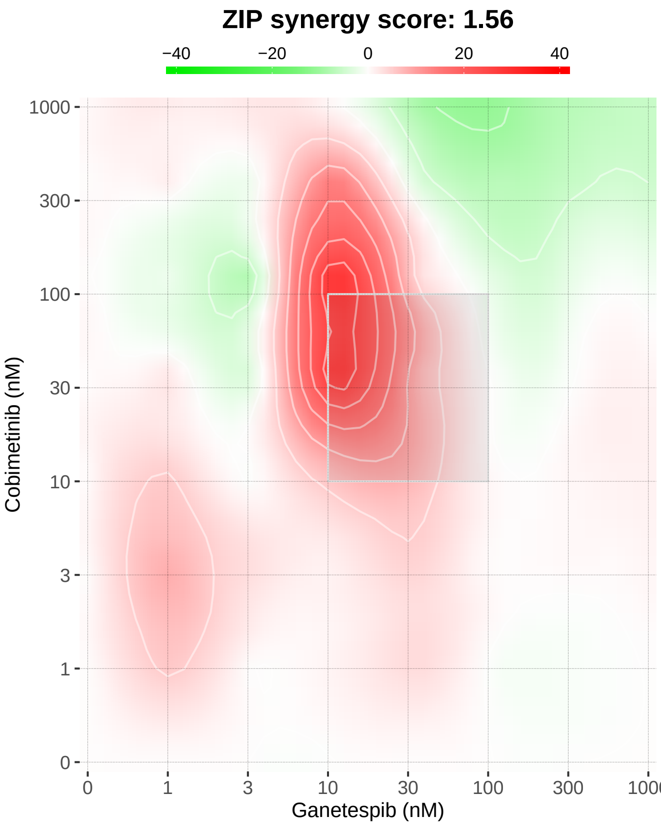

BOWES

Cobimetinib (nM) & Ulixertinib (nM)

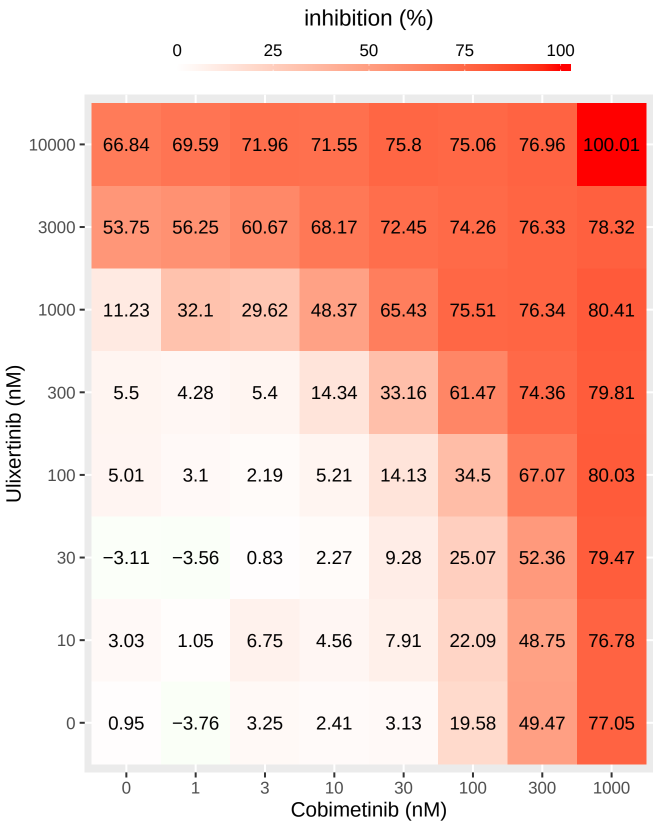

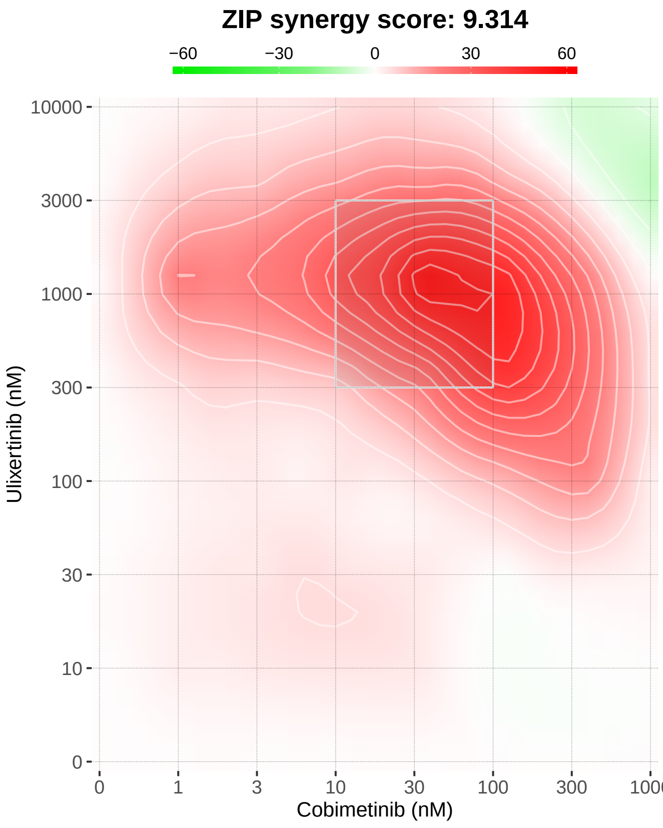

BOWES

SCH772984 (nM) & Cobimetinib (nM)

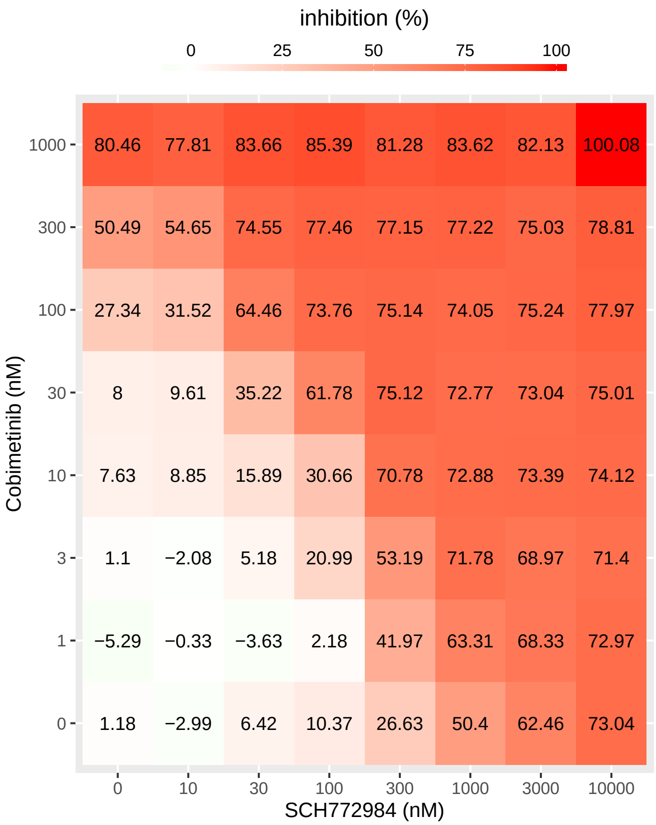

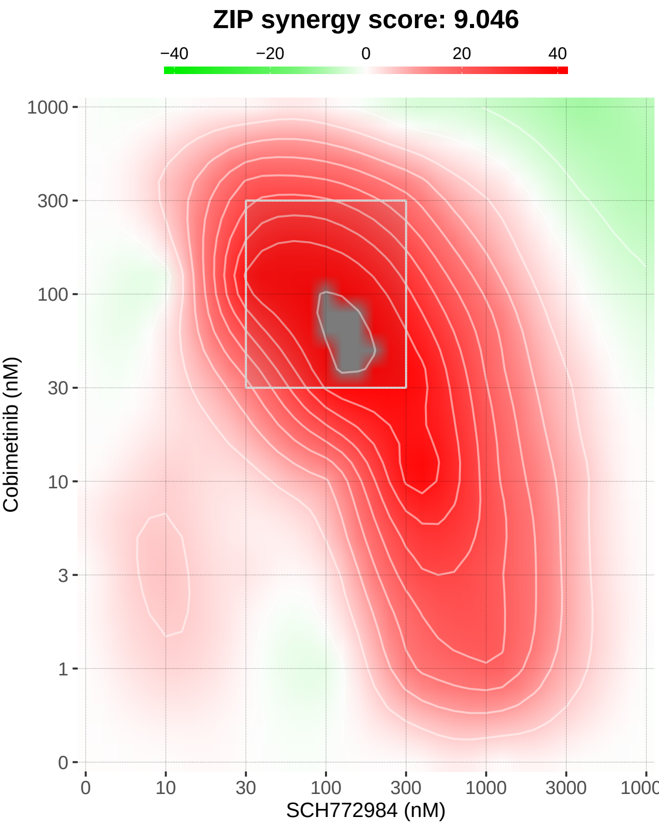

BOWES

Cobimetinib (nM) & LY3009120 (nM)

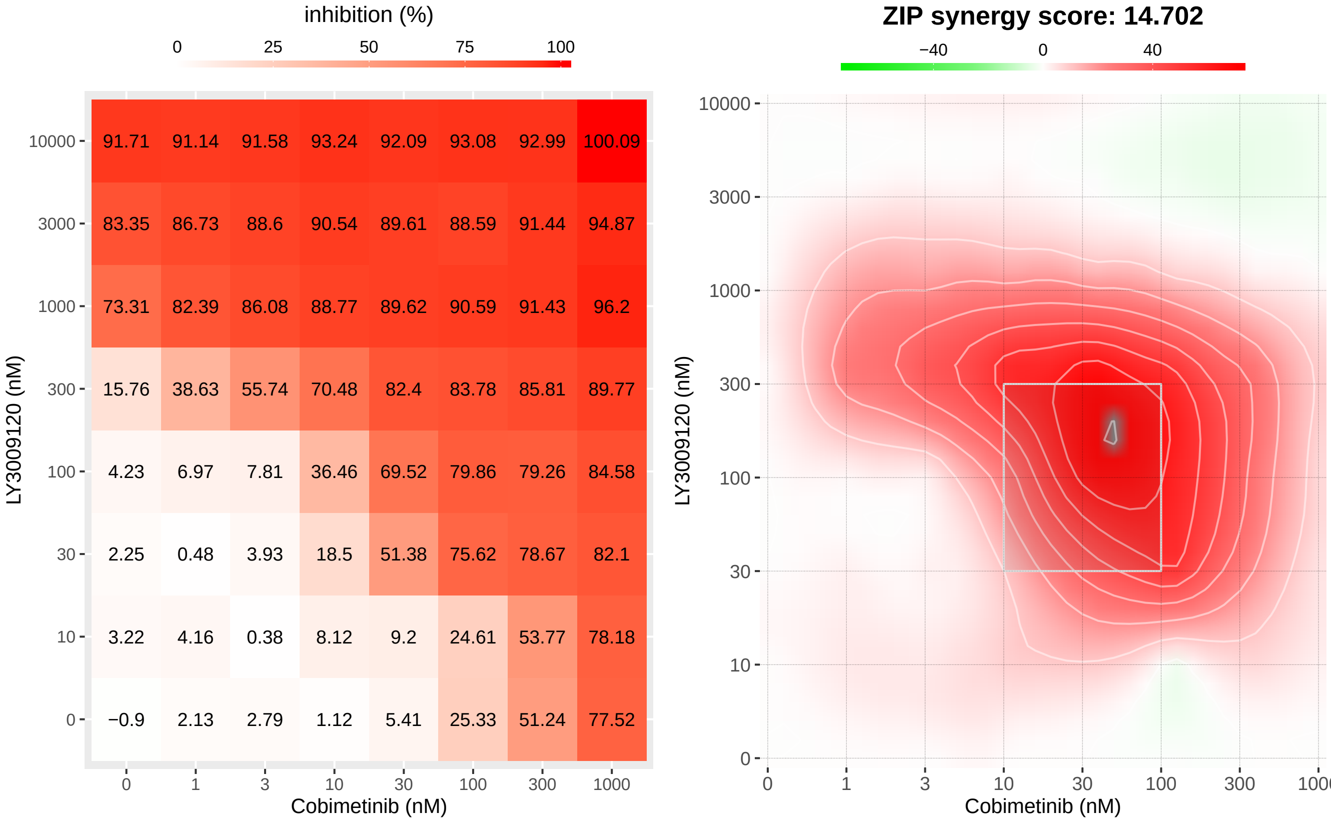

BOWES

Cobimetinib (nM) & BI 2536 (nM)

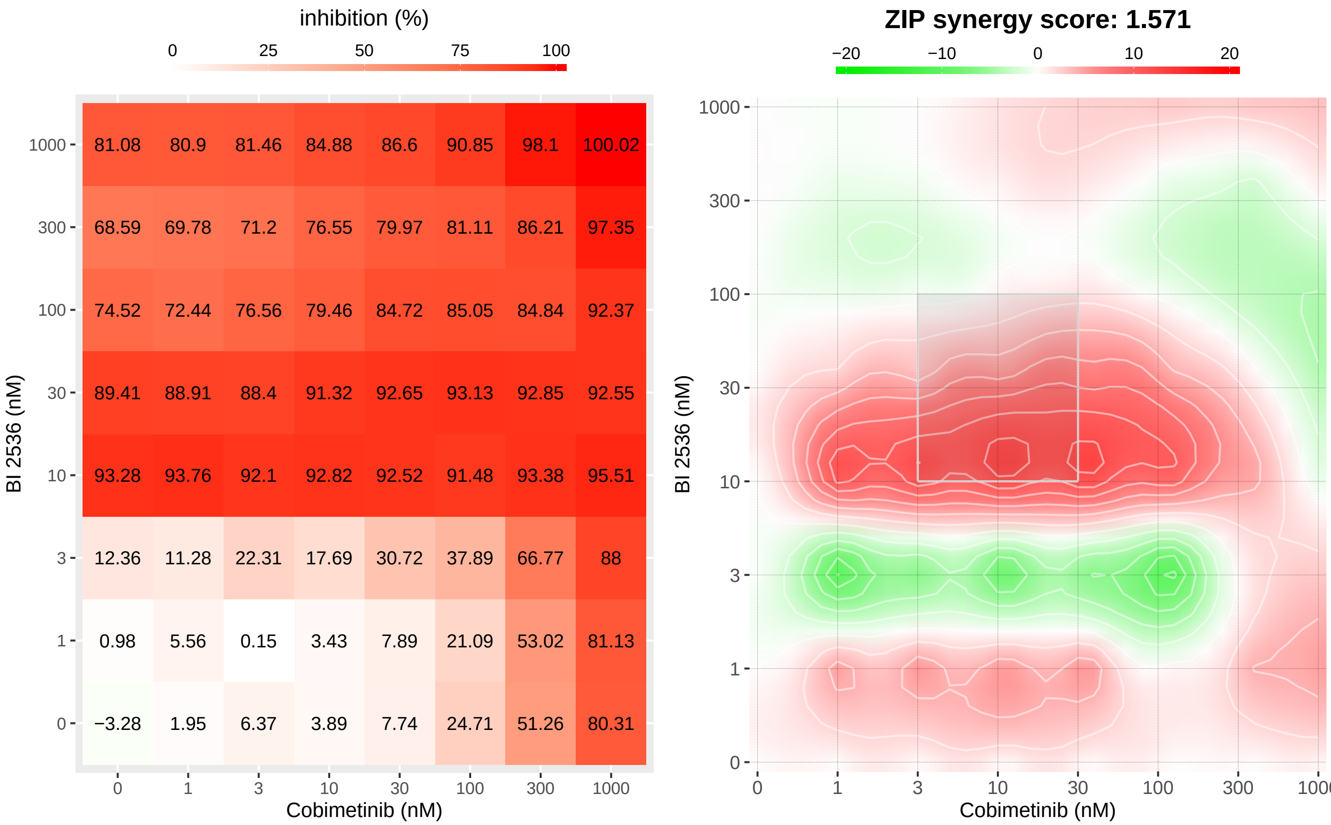

BOWES

Cobimetinib (nM) & Palbociclib (nM)

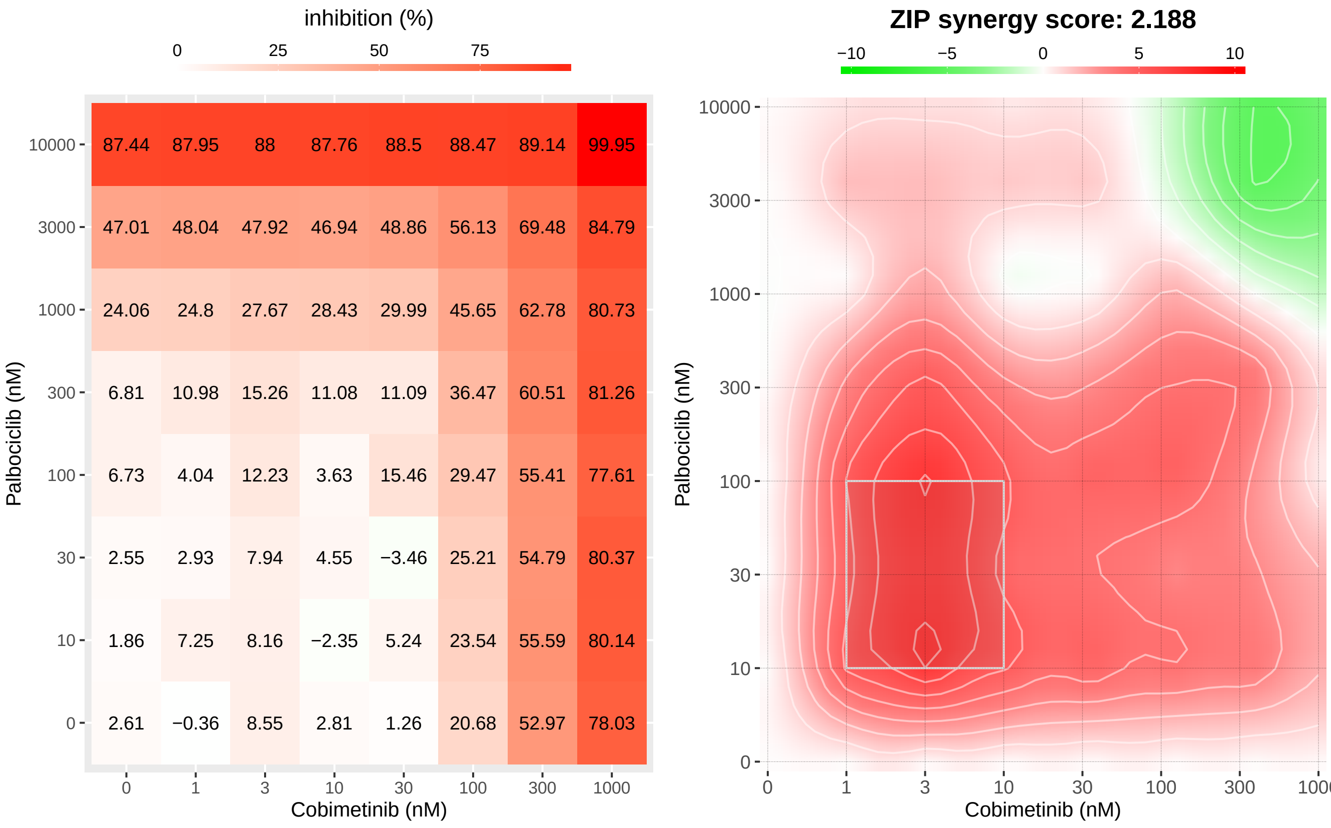

Supplement: Supplementary file 2 [file mmc2.pdf]
